# Supplementary material for: Feature Selection and Molecular Classification of Cancer Phenotypes: A Comparative Study
Source: Int J Mol Sci. 2022 Aug 13;23(16):9087. doi: 10.3390/ijms23169087 (PMC9408964; doi:10.3390/ijms23169087)
Supplement: Supplementary file 1 [file ijms-23-09087-s001.zip › Supplementary Figures.pdf]

Supplementary Figures

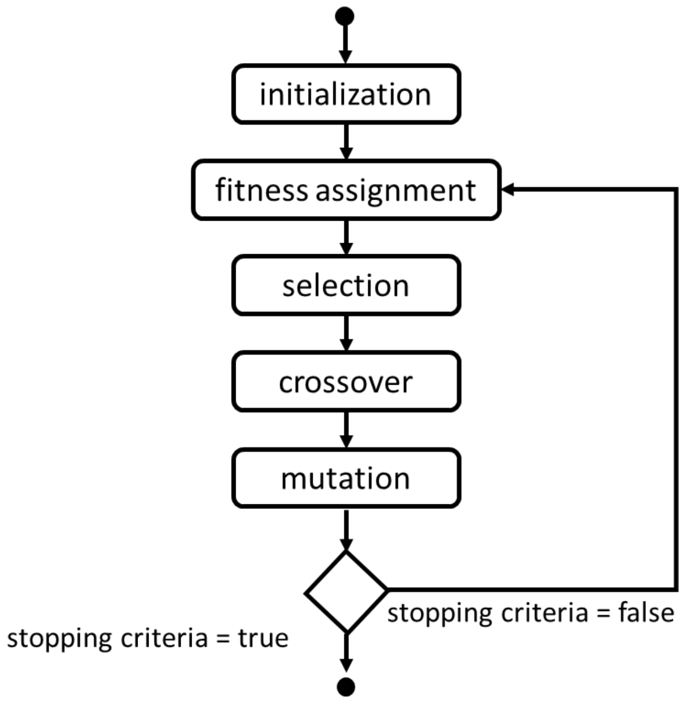

Supplementary Figure S1. Pipeline employed in Genetic Algorithms for feature selection.

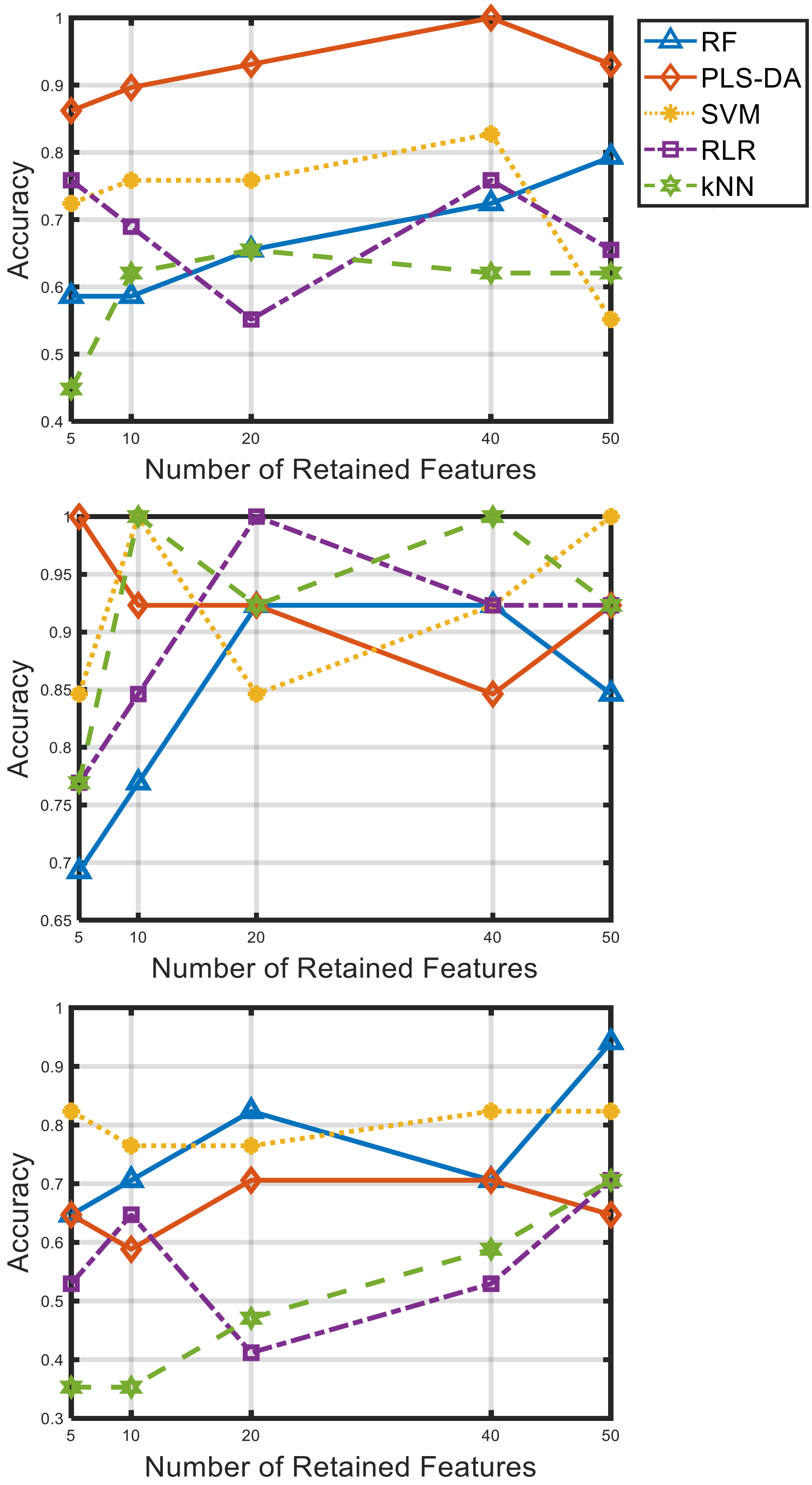

Supplementary Figure S2. Predictive accuracy of RF, PLS-DA, SVM, RLR/RMR and kNN vs Number of Retained Features (5, 10, 20, 40, 50) on: (top) SMK_CAN_187, (mid) GLI_85, (bottom) CLL_SUB_111 using GA-based wrappers for feature selection. Population Size = 200; maximum number of generations = 150. Each point corresponds to a single value for the predictive accuracy.

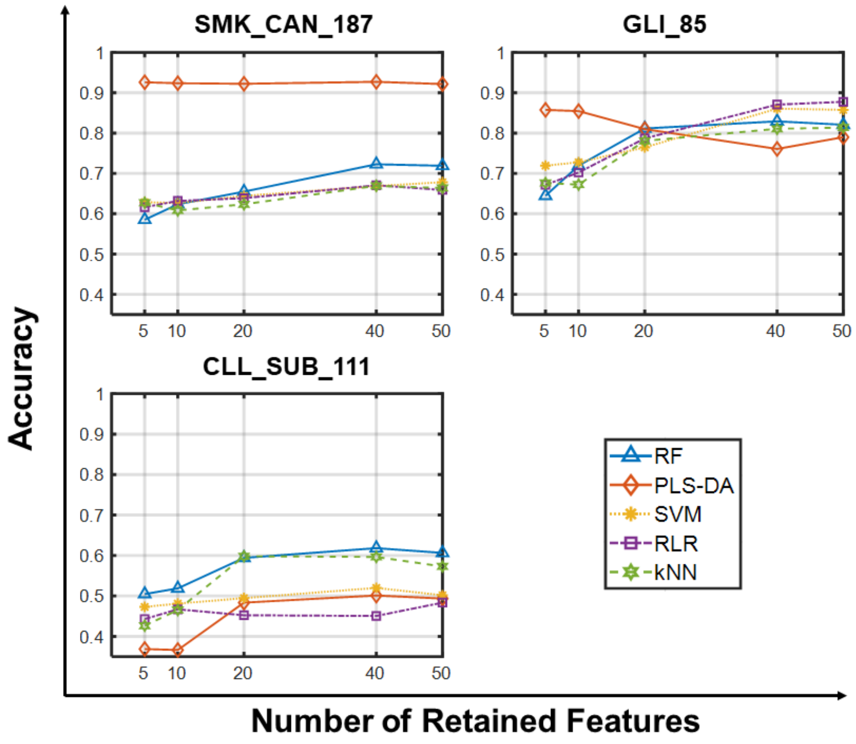

Supplementary Figure S3. Predictive accuracy of RF, PLS-DA, SVM, RLR/RMR and kNN vs Number of Retained Features on SMK_CAN_187, GLI_85, CLL_SUB_111 selecting 5, 10, 20, 40, 50 most variable genes. Each point is the average predictive accuracy computed over 100 resamplings.

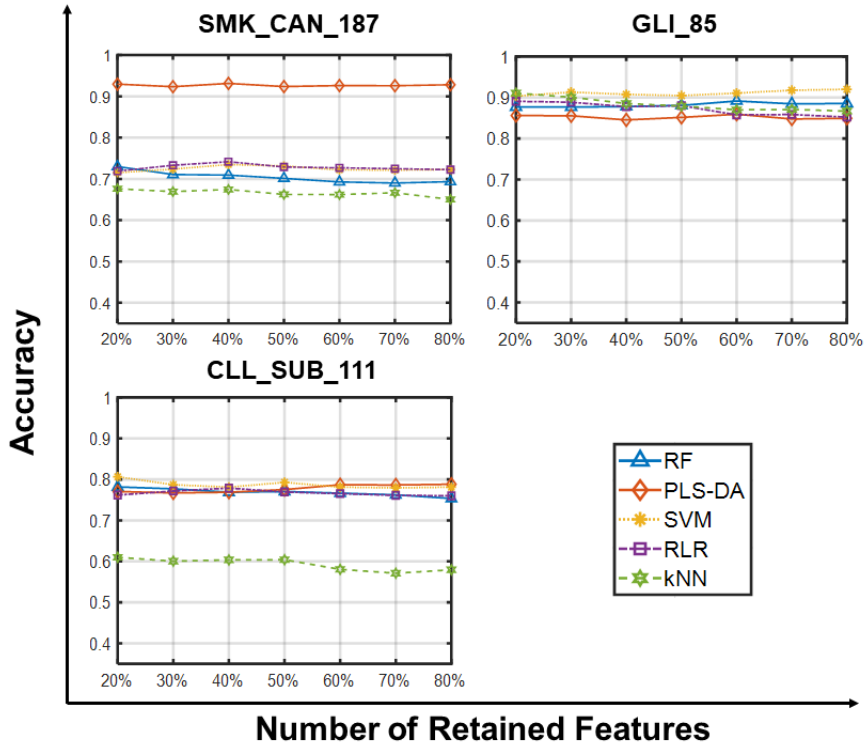

Supplementary Figure S4. Predictive accuracy of RF, PLS-DA, SVM, RLR/RMR and kNN vs Number of Retained Features on SMK_CAN_187, GLI_85, CLL_SUB_111 selecting the 20%, 30%, 40%, 50%, 60%, 70%, 80% of the genes sorted by variance. Each point is the average predictive accuracy computed over 100 resamplings.
