# Supplementary material for: Feature Selection and Molecular Classification of Cancer Phenotypes: A Comparative Study
Source: Int J Mol Sci. 2022 Aug 13;23(16):9087. doi: 10.3390/ijms23169087 (PMC9408964; doi:10.3390/ijms23169087)
Supplement: Supplementary file 1 [file ijms-23-09087-s001.zip › Supplementary Tables.pdf]

Rsp100_Biomarkers_SMK_CAN_187

mRMR

Accuracy	mean accuracy ± 95% CI				
**Classifier**	**5**	**10**	**20**	**40**	**50**
'RF'	'68.69% ± 1.65%'	'68.69% ± 1.76%'	'69.1% ± 1.67%'	'69.17% ± 1.64%'	'69.17% ± 1.49%'
'PLS-DA'	'92.21% ± 1.03%'	'92.21% ± 1.19%'	'92.07% ± 1.03%'	'93.14% ± 1.13%'	'92% ± 1.12%'
'SVM'	'69.31% ± 1.55%'	'69.66% ± 1.81%'	'68.86% ± 1.66%'	'69.83% ± 1.59%'	'69.76% ± 1.57%'
'RLR'	'69.24% ± 1.6%'	'68.24% ± 1.77%'	'67.62% ± 1.78%'	'69.1% ± 1.67%'	'68.45% ± 1.64%'
'kNN'	'68.83% ± 1.51%'	'67.72% ± 1.58%'	'67.93% ± 1.62%'	'68.86% ± 1.6%'	'68.48% ± 1.4%'

Recall	mean recall ± 95% CI				
**Classifier**	**5**	**10**	**20**	**40**	**50**
'RF'	'66.51% ± 2.51%'	'64.33% ± 2.73%'	'63.86% ± 2.82%'	'61.99% ± 2.63%'	'61.82% ± 2.63%'
'PLS-DA'	'93.71% ± 2%'	'92.6% ± 2.27%'	'90.25% ± 2.4%'	'91.46% ± 2.44%'	'89.59% ± 2.62%'
'SVM'	'64.42% ± 2.67%'	'61.8% ± 2.8%'	'61.19% ± 2.62%'	'62.03% ± 2.82%'	'61.61% ± 2.92%'
'RLR'	'66.95% ± 2.54%'	'66.09% ± 2.65%'	'64.77% ± 2.69%'	'66.34% ± 2.45%'	'65.47% ± 2.51%'
'kNN'	'65.34% ± 2.47%'	'62.57% ± 2.46%'	'62.39% ± 2.46%'	'58.68% ± 2.35%'	'57.13% ± 2.3%'

Precision	mean precision ± 95% CI				
**Classifier**	**5**	**10**	**20**	**40**	**50**
'RF'	'67.4% ± 2.15%'	'68.13% ± 2.31%'	'69.35% ± 2.37%'	'69.78% ± 2.21%'	'70.11% ± 2.2%'
'PLS-DA'	'91.58% ± 1.83%'	'92.65% ± 1.89%'	'94.05% ± 1.69%'	'94.86% ± 1.58%'	'94.46% ± 1.63%'
'SVM'	'68.82% ± 2.23%'	'71.02% ± 2.56%'	'70.07% ± 2.4%'	'71.02% ± 2.21%'	'70.81% ± 2.14%'
'RLR'	'67.71% ± 2.09%'	'66.82% ± 2.25%'	'66.19% ± 2.17%'	'67.91% ± 2.22%'	'67.21% ± 2.16%'
'kNN'	'67.83% ± 1.94%'	'67.22% ± 2.08%'	'67.49% ± 2.26%'	'70.56% ± 2.32%'	'70.29% ± 2.05%'

Specificity	mean specificity ± 95% CI				
**Classifier**	**5**	**10**	**20**	**40**	**50**
'RF'	'70.59% ± 2.58%'	'72.46% ± 2.54%'	'73.75% ± 2.67%'	'75.4% ± 2.47%'	'75.51% ± 2.4%'
'PLS-DA'	'90.87% ± 2.14%'	'91.8% ± 2.29%'	'93.72% ± 1.84%'	'94.63% ± 1.75%'	'94.03% ± 1.85%'
'SVM'	'73.51% ± 2.26%'	'76.47% ± 2.67%'	'75.57% ± 2.54%'	'76.62% ± 2.32%'	'76.8% ± 2.31%'
'RLR'	'71.19% ± 2.36%'	'70.05% ± 2.68%'	'70.09% ± 2.5%'	'71.47% ± 2.45%'	'71.01% ± 2.47%'
'kNN'	'71.76% ± 2.51%'	'72.16% ± 2.42%'	'72.72% ± 2.37%'	'77.67% ± 2.29%'	'78.3% ± 1.9%'

NPV	mean NPV ± 95% CI				
**Classifier**	**5**	**10**	**20**	**40**	**50**
'RF'	'71.17% ± 1.66%'	'70.53% ± 1.79%'	'70.71% ± 1.7%'	'69.9% ± 1.57%'	'69.9% ± 1.5%'
'PLS-DA'	'95.4% ± 1.41%'	'94.67% ± 1.55%'	'92.96% ± 1.66%'	'93.9% ± 1.62%'	'92.67% ± 1.76%'
'SVM'	'71.03% ± 1.59%'	'70.12% ± 1.7%'	'69.52% ± 1.66%'	'70.44% ± 1.6%'	'70.37% ± 1.59%'
'RLR'	'71.82% ± 1.66%'	'70.83% ± 1.8%'	'70.11% ± 1.87%'	'71.32% ± 1.61%'	'70.69% ± 1.64%'
'kNN'	'70.85% ± 1.5%'	'69.22% ± 1.59%'	'69.31% ± 1.55%'	'68.53% ± 1.42%'	'68.03% ± 1.27%'

F1	mean F1 ± 95% CI				
**Classifier**	**5**	**10**	**20**	**40**	**50**

'RF'	'66.13% ± 1.89%'	'65.28% ± 2.07%'	'65.33% ± 2.02%'	'64.77% ± 2.04%'	'64.62% ± 1.92%'
'PLS-DA'	'91.79% ± 1.1%'	'91.64% ± 1.32%'	'91.13% ± 1.24%'	'92.22% ± 1.43%'	'90.9% ± 1.39%'
'SVM'	'65.67% ± 1.98%'	'65.04% ± 2.21%'	'64.28% ± 1.96%'	'65.08% ± 2.14%'	'64.78% ± 2.2%'
'RLR'	'66.63% ± 1.88%'	'65.66% ± 2.01%'	'64.73% ± 2.01%'	'66.43% ± 1.91%'	'65.63% ± 1.92%'
'kNN'	'65.79% ± 1.78%'	'64.02% ± 1.84%'	'64.11% ± 1.94%'	'63.38% ± 2.01%'	'62.4% ± 1.88%'

Relief-F

Accuracy — mean accuracy ± 95% CI

Classifier	5	10	20	40	50
'RF'	'63.55% ± 1.61%'	'67.17% ± 1.37%'	'67.93% ± 1.58%'	'66.9% ± 1.46%'	'67.45% ± 1.59%'
'PLS-DA'	'92.14% ± 1.16%'	'92.66% ± 1.13%'	'92.97% ± 1.05%'	'93.59% ± 1.07%'	'93.69% ± 1.08%'
'SVM'	'67.34% ± 1.72%'	'67.41% ± 1.66%'	'68.41% ± 1.61%'	'67.55% ± 1.64%'	'67.83% ± 1.66%'
'RLR'	'67.66% ± 1.66%'	'67.83% ± 1.62%'	'68.38% ± 1.59%'	'68.52% ± 1.58%'	'69.34% ± 1.7%'
'kNN'	'65.48% ± 1.79%'	'67.31% ± 1.57%'	'68.9% ± 1.57%'	'66.86% ± 1.59%'	'67.55% ± 1.62%'

Recall — mean recall ± 95% CI

Classifier	5	10	20	40	50
'RF'	'60.42% ± 2.42%'	'61.45% ± 2.38%'	'61.24% ± 2.58%'	'57.82% ± 2.49%'	'58.63% ± 2.65%'
'PLS-DA'	'94.44% ± 1.92%'	'93.01% ± 2.32%'	'93.53% ± 2.02%'	'93.08% ± 1.91%'	'94.23% ± 1.87%'
'SVM'	'62.74% ± 2.6%'	'59.77% ± 2.65%'	'60.14% ± 2.56%'	'56.81% ± 2.82%'	'58.07% ± 2.72%'
'RLR'	'65.65% ± 2.65%'	'64.65% ± 2.62%'	'65.73% ± 2.56%'	'65.29% ± 2.63%'	'65.73% ± 2.44%'
'kNN'	'57.64% ± 2.81%'	'57.93% ± 2.33%'	'60.07% ± 2.31%'	'55.77% ± 2.37%'	'57.25% ± 2.27%'

Precision — mean precision ± 95% CI

Classifier	5	10	20	40	50
'RF'	'61.53% ± 2.05%'	'66.43% ± 1.84%'	'67.6% ± 2.01%'	'67.37% ± 2.06%'	'67.74% ± 2.17%'
'PLS-DA'	'91.06% ± 2%'	'92.84% ± 1.75%'	'92.97% ± 1.73%'	'94.53% ± 1.68%'	'93.76% ± 1.72%'
'SVM'	'66.27% ± 2.1%'	'67.59% ± 2.25%'	'69.37% ± 2.32%'	'69.38% ± 2.36%'	'69.18% ± 2.4%'
'RLR'	'65.48% ± 1.92%'	'66.42% ± 2.05%'	'66.83% ± 2.01%'	'66.97% ± 1.92%'	'68.17% ± 2.2%'
'kNN'	'65.12% ± 2.3%'	'68.2% ± 2.21%'	'70.03% ± 2.17%'	'68.55% ± 2.23%'	'68.91% ± 2.34%'

Specificity — mean specificity ± 95% CI

Classifier	5	10	20	40	50
'RF'	'66.21% ± 2.29%'	'72.14% ± 2.11%'	'73.72% ± 2.09%'	'74.75% ± 2.06%'	'75.03% ± 2.09%'
'PLS-DA'	'90.12% ± 2.37%'	'92.3% ± 2%'	'92.43% ± 2.02%'	'94.06% ± 1.98%'	'93.19% ± 2.03%'
'SVM'	'71.34% ± 2.32%'	'74.06% ± 2.24%'	'75.6% ± 2.41%'	'76.9% ± 2.47%'	'76.34% ± 2.39%'
'RLR'	'69.34% ± 2.22%'	'70.52% ± 2.28%'	'70.63% ± 2.26%'	'71.3% ± 2.13%'	'72.47% ± 2.21%'
'kNN'	'72.23% ± 2.33%'	'75.5% ± 2.17%'	'76.55% ± 2.17%'	'76.58% ± 2.31%'	'76.58% ± 2.16%'

NPV — mean NPV ± 95% CI

Classifier	5	10	20	40	50
'RF'	'66.06% ± 1.57%'	'68.72% ± 1.4%'	'69.08% ± 1.62%'	'67.48% ± 1.45%'	'68.07% ± 1.57%'
'PLS-DA'	'95.93% ± 1.35%'	'95.04% ± 1.54%'	'95.29% ± 1.4%'	'94.8% ± 1.36%'	'95.73% ± 1.32%'
'SVM'	'69.15% ± 1.76%'	'68.35% ± 1.64%'	'68.9% ± 1.51%'	'67.67% ± 1.59%'	'68.04% ± 1.54%'
'RLR'	'70.5% ± 1.79%'	'70.27% ± 1.74%'	'70.96% ± 1.78%'	'70.92% ± 1.76%'	'71.29% ± 1.72%'

'kNN'	'66.71% ± 1.75%'	'67.55% ± 1.39%'	'69.04% ± 1.49%'	'66.71% ± 1.46%'	'67.45% ± 1.46%'

F1 mean F1 ± 95% CI

Classifier	5	10	20	40	50
'RF'	'60.38% ± 1.89%'	'63.16% ± 1.72%'	'63.55% ± 1.99%'	'61.45% ± 1.91%'	'62.14% ± 2.13%'
'PLS-DA'	'91.86% ± 1.18%'	'91.99% ± 1.35%'	'92.46% ± 1.15%'	'93.1% ± 1.13%'	'93.31% ± 1.15%'
'SVM'	'63.78% ± 2.03%'	'62.61% ± 2.09%'	'63.52% ± 2.04%'	'61.35% ± 2.21%'	'62.11% ± 2.25%'
'RLR'	'64.99% ± 1.99%'	'64.76% ± 1.93%'	'65.59% ± 1.85%'	'65.46% ± 1.91%'	'66.37% ± 1.98%'
'kNN'	'60.34% ± 2.23%'	'61.92% ± 2%'	'64% ± 1.85%'	'60.71% ± 1.95%'	'61.93% ± 1.96%'

Chi-Squared

Accuracy mean accuracy ± 95% CI

Classifier	5	10	20	40	50
'RF'	'63.83% ± 1.71%'	'65.45% ± 1.66%'	'66.93% ± 1.45%'	'67.45% ± 1.45%'	'67.59% ± 1.38%'
'PLS-DA'	'92.03% ± 1.13%'	'92.72% ± 1.1%'	'92.79% ± 1.04%'	'93.24% ± 1.09%'	'92.24% ± 1.17%'
'SVM'	'64.41% ± 1.56%'	'65.07% ± 1.68%'	'67.07% ± 1.57%'	'67.76% ± 1.46%'	'68.41% ± 1.57%'
'RLR'	'64.28% ± 1.59%'	'65.83% ± 1.72%'	'66.38% ± 1.48%'	'67.31% ± 1.54%'	'68.03% ± 1.72%'
'kNN'	'64.79% ± 1.6%'	'65.59% ± 1.52%'	'66.66% ± 1.32%'	'67.31% ± 1.44%'	'67.21% ± 1.63%'

Recall mean recall ± 95% CI

Classifier	5	10	20	40	50
'RF'	'60.99% ± 2.4%'	'60.66% ± 2.51%'	'61% ± 2.54%'	'60.59% ± 2.42%'	'61.57% ± 2.37%'
'PLS-DA'	'93.92% ± 2.02%'	'95.47% ± 1.58%'	'94.24% ± 1.87%'	'92.98% ± 2.2%'	'91.84% ± 2.07%'
'SVM'	'59.53% ± 2.83%'	'60.05% ± 2.36%'	'60.93% ± 2.59%'	'59.39% ± 2.22%'	'60.7% ± 2.45%'
'RLR'	'60.05% ± 2.86%'	'63.9% ± 2.34%'	'63.36% ± 2.39%'	'63.44% ± 2.19%'	'64.96% ± 2.34%'
'kNN'	'63.02% ± 2.69%'	'61.63% ± 2.1%'	'62.25% ± 2.38%'	'62.1% ± 2.15%'	'61.82% ± 2.22%'

Precision mean precision ± 95% CI

Classifier	5	10	20	40	50
'RF'	'61.93% ± 2.09%'	'63.99% ± 1.99%'	'66.04% ± 1.85%'	'67.33% ± 1.86%'	'67.19% ± 1.84%'
'PLS-DA'	'91.25% ± 1.97%'	'91.21% ± 1.95%'	'92.19% ± 1.81%'	'93.88% ± 1.66%'	'93.41% ± 1.97%'
'SVM'	'62.73% ± 1.94%'	'64.19% ± 2.17%'	'66.65% ± 2.03%'	'68.63% ± 2.09%'	'68.61% ± 2.07%'
'RLR'	'62.35% ± 1.99%'	'64.09% ± 2.1%'	'64.65% ± 1.78%'	'66% ± 1.91%'	'66.74% ± 2.13%'
'kNN'	'62.31% ± 1.82%'	'64.14% ± 1.98%'	'65.39% ± 1.72%'	'66.69% ± 1.93%'	'66.34% ± 2.09%'

Specificity mean specificity ± 95% CI

Classifier	5	10	20	40	50
'RF'	'66.25% ± 2.5%'	'69.63% ± 2.28%'	'72.05% ± 2.05%'	'73.41% ± 2.27%'	'72.86% ± 2.17%'
'PLS-DA'	'90.39% ± 2.32%'	'90.27% ± 2.33%'	'91.52% ± 2.1%'	'93.49% ± 1.89%'	'92.58% ± 2.35%'
'SVM'	'68.64% ± 2.22%'	'69.32% ± 2.61%'	'72.33% ± 2.35%'	'75.03% ± 2.38%'	'75.09% ± 2.14%'
'RLR'	'67.95% ± 2.3%'	'67.44% ± 2.59%'	'68.93% ± 2.24%'	'70.64% ± 2.21%'	'70.7% ± 2.45%'
'kNN'	'66.31% ± 2.18%'	'68.95% ± 2.31%'	'70.42% ± 2.09%'	'71.75% ± 2.23%'	'71.85% ± 2.15%'

NPV mean NPV ± 95% CI

Classifier	5	10	20	40	50

'RF'	'66.25% ± 2.5%'	'69.63% ± 2.28%'	'72.05% ± 2.05%'	'73.41% ± 2.27%'	'72.86% ± 2.17%'
'PLS-DA'	'90.39% ± 2.32%'	'90.27% ± 2.33%'	'91.52% ± 2.1%'	'93.49% ± 1.89%'	'92.58% ± 2.35%'
'SVM'	'68.64% ± 2.22%'	'69.32% ± 2.61%'	'72.33% ± 2.35%'	'75.03% ± 2.38%'	'75.09% ± 2.14%'
'RLR'	'67.95% ± 2.3%'	'67.44% ± 2.59%'	'68.93% ± 2.24%'	'70.64% ± 2.21%'	'70.7% ± 2.45%'
'kNN'	'66.31% ± 2.18%'	'68.95% ± 2.31%'	'70.42% ± 2.09%'	'71.75% ± 2.23%'	'71.85% ± 2.15%'

F1 mean F1 ± 95% CI

Classifier	5	10	20	40	50
'RF'	'61.89% ± 2.78%'	'62.53% ± 2.79%'	'62.8% ± 2.79%'	'63.84% ± 2.92%'	'63.37% ± 2.57%'
'PLS-DA'	'90.59% ± 1.83%'	'91.08% ± 1.65%'	'90.94% ± 1.88%'	'92.49% ± 1.96%'	'91.38% ± 1.56%'
'SVM'	'60.99% ± 3.2%'	'62.28% ± 2.88%'	'63.95% ± 2.44%'	'62.61% ± 2.74%'	'64.25% ± 2.7%'
'RLR'	'63.06% ± 2.87%'	'64.14% ± 3.09%'	'63.81% ± 2.73%'	'64.75% ± 2.23%'	'65.77% ± 2.43%'
'kNN'	'62.89% ± 2.81%'	'62.52% ± 2.66%'	'64.44% ± 2.47%'	'63.39% ± 2.4%'	'63.23% ± 2.59%'

Variance

Accuracy mean accuracy ± 95% CI

Classifier	5	10	20	40	50
'RF'	'58.52% ± 2.33%'	'62.34% ± 2.12%'	'65.48% ± 2.36%'	'72.28% ± 2.02%'	'71.9% ± 1.81%'
'PLS-DA'	'92.62% ± 1.71%'	'92.38% ± 1.77%'	'92.24% ± 1.47%'	'92.72% ± 1.5%'	'92.17% ± 1.55%'
'SVM'	'62.86% ± 2.29%'	'62.66% ± 2.26%'	'64.41% ± 2.39%'	'66.86% ± 2.5%'	'67.83% ± 2.39%'
'RLR'	'61.62% ± 2.35%'	'63.17% ± 2.31%'	'63.86% ± 2.39%'	'67.07% ± 2.68%'	'65.9% ± 2.47%'
'kNN'	'62.72% ± 2.32%'	'60.83% ± 2.12%'	'62.34% ± 2.34%'	'66.93% ± 2.41%'	'66.38% ± 2.19%'

Recall mean recall ± 95% CI

Classifier	5	10	20	40	50
'RF'	'55.34% ± 3.65%'	'57.34% ± 3.14%'	'57.19% ± 3.66%'	'59.08% ± 3.33%'	'59.02% ± 3.37%'
'PLS-DA'	'89.59% ± 3.76%'	'88.71% ± 3.96%'	'89.82% ± 3.63%'	'89.23% ± 3.56%'	'89.09% ± 3.59%'
'SVM'	'47.57% ± 3.75%'	'45.9% ± 3.67%'	'48.51% ± 3.61%'	'52.92% ± 3.65%'	'54.64% ± 3.75%'
'RLR'	'51.4% ± 3.88%'	'52.59% ± 3.69%'	'54.8% ± 3.95%'	'59.84% ± 4.09%'	'58.69% ± 3.76%'
'kNN'	'54.21% ± 4.2%'	'49.5% ± 3.74%'	'49.58% ± 3.48%'	'51.24% ± 3.86%'	'51.04% ± 3.44%'

Precision mean precision ± 95% CI

Classifier	5	10	20	40	50
'RF'	'55.61% ± 2.63%'	'60.8% ± 2.63%'	'64.99% ± 3%'	'77.44% ± 3.18%'	'76.66% ± 2.93%'
'PLS-DA'	'95.56% ± 2.13%'	'95.87% ± 2.07%'	'94.58% ± 2.11%'	'95.94% ± 1.97%'	'95.16% ± 2.2%'
'SVM'	'64.04% ± 3.34%'	'64.18% ± 3.45%'	'66.74% ± 3.61%'	'70.09% ± 3.64%'	'70.77% ± 3.48%'
'RLR'	'60.62% ± 3.05%'	'62.83% ± 3.13%'	'63.29% ± 3.17%'	'66.95% ± 3.5%'	'65.48% ± 3.23%'
'kNN'	'61.22% ± 2.89%'	'59.8% ± 3.03%'	'62.87% ± 3.47%'	'71.07% ± 3.69%'	'70.09% ± 3.4%'

Specificity mean specificity ± 95% CI

Classifier	5	10	20	40	50
'RF'	'61.25% ± 3.15%'	'66.66% ± 3.47%'	'72.64% ± 3.01%'	'83.8% ± 2.86%'	'83.16% ± 2.69%'
'PLS-DA'	'95.2% ± 2.46%'	'95.51% ± 2.43%'	'94.28% ± 2.35%'	'95.72% ± 2.14%'	'94.84% ± 2.49%'
'SVM'	'76.12% ± 2.92%'	'77.16% ± 2.72%'	'78.2% ± 2.93%'	'78.95% ± 3.33%'	'79.29% ± 3.24%'
'RLR'	'70.5% ± 3.08%'	'72.36% ± 2.92%'	'71.69% ± 3.18%'	'73.29% ± 3.43%'	'72.16% ± 3.25%'

'kNN'	'70.02% ± 3.04%'	'70.61% ± 2.95%'	'73.33% ± 3.36%'	'80.64% ± 3.16%'	'79.72% ± 3.17%'

NPV	mean NPV ± 95% CI				
**Classifier**	**5**	**10**	**20**	**40**	**50**
'RF'	'61.47% ± 2.34%'	'64.29% ± 2.04%'	'66.45% ± 2.25%'	'70.48% ± 1.96%'	'70.37% ± 1.85%'
'PLS-DA'	'92.68% ± 2.46%'	'92.1% ± 2.54%'	'92.8% ± 2.46%'	'92.3% ± 2.37%'	'92.23% ± 2.42%'
'SVM'	'62.84% ± 2.06%'	'62.38% ± 1.95%'	'63.82% ± 2.1%'	'66.03% ± 2.29%'	'67.04% ± 2.13%'
'RLR'	'62.86% ± 2.21%'	'64.02% ± 2.16%'	'65% ± 2.33%'	'68.17% ± 2.64%'	'67.1% ± 2.42%'
'kNN'	'64.42% ± 2.45%'	'62.02% ± 1.91%'	'62.67% ± 1.97%'	'65.82% ± 2.22%'	'65.31% ± 1.96%'

F1	mean F1 ± 95% CI				
**Classifier**	**5**	**10**	**20**	**40**	**50**
'RF'	'54.96% ± 2.83%'	'58.39% ± 2.39%'	'60.23% ± 3.01%'	'66.11% ± 2.6%'	'65.69% ± 2.48%'
'PLS-DA'	'91.46% ± 2.19%'	'91.06% ± 2.36%'	'91.15% ± 1.88%'	'91.57% ± 1.97%'	'91.03% ± 1.93%'
'SVM'	'53.72% ± 3.23%'	'52.71% ± 3.26%'	'55.42% ± 3.22%'	'59.39% ± 3.1%'	'60.76% ± 3.16%'
'RLR'	'54.87% ± 3.19%'	'56.54% ± 3.05%'	'57.93% ± 3.15%'	'62.89% ± 3.11%'	'61.16% ± 3.03%'
'kNN'	'56.71% ± 3.29%'	'53.4% ± 3.02%'	'54.61% ± 3.07%'	'58.46% ± 3.22%'	'58.09% ± 2.91%'

Genetic Algorithm: PopSize = 50, Max NumGen= 35

Accuracy	accuracy				
**Classifier**	**5**	**10**	**20**	**40**	**50**
'RF'	'55.17%'	'65.52%'	'75.86%'	'62.07%'	'75.86%'
'PLS-DA'	'100%'	'89.66%'	'96.55%'	'89.66%'	'96.55%'
'SVM'	'62.07%'	'75.86%'	'68.97%'	'72.41%'	'62.07%'
'RMR'	'79.31%'	'75.86%'	'72.41%'	'68.97%'	'75.86%'
'kNN'	'65.52%'	'55.17%'	'65.52%'	'79.31%'	'51.72%'

Recall	recall				
**Classifier**	**5**	**10**	**20**	**40**	**50**
'RF'	'35.71%'	'42.86%'	'71.43%'	'50%'	'57.14%'
'PLS-DA'	'100%'	'100%'	'100%'	'78.57%'	'92.86%'
'SVM'	'35.71%'	'57.14%'	'42.86%'	'64.29%'	'28.57%'
'RMR'	'71.43%'	'71.43%'	'57.14%'	'71.43%'	'57.14%'
'kNN'	'64.29%'	'35.71%'	'64.29%'	'64.29%'	'57.14%'

Precision	precision				
**Classifier**	**5**	**10**	**20**	**40**	**50**
'RF'	'55.56%'	'75%'	'76.92%'	'63.64%'	'88.89%'
'PLS-DA'	'100%'	'82.35%'	'93.33%'	'100%'	'100%'
'SVM'	'71.43%'	'88.89%'	'85.71%'	'75%'	'80%'
'RMR'	'83.33%'	'76.92%'	'80%'	'66.67%'	'88.89%'
'kNN'	'64.29%'	'55.56%'	'64.29%'	'90%'	'50%'

Specificity	specificity				
**Classifier**	**5**	**10**	**20**	**40**	**50**

'RF'	'73.33%'	'86.67%'	'80%'	'73.33%'	'93.33%'
'PLS-DA'	'100%'	'80%'	'93.33%'	'100%'	'100%'
'SVM'	'86.67%'	'93.33%'	'93.33%'	'80%'	'93.33%'
'RMR'	'86.67%'	'80%'	'86.67%'	'66.67%'	'93.33%'
'kNN'	'66.67%'	'73.33%'	'66.67%'	'93.33%'	'46.67%'

NPV	NPV				
**Classifier**	**5**	**10**	**20**	**40**	**50**
'RF'	'55%'	'61.9%'	'75%'	'61.11%'	'70%'
'PLS-DA'	'100%'	'100%'	'100%'	'83.33%'	'93.75%'
'SVM'	'59.09%'	'70%'	'63.64%'	'70.59%'	'58.33%'
'RMR'	'76.47%'	'75%'	'68.42%'	'71.43%'	'70%'
'kNN'	'66.67%'	'55%'	'66.67%'	'73.68%'	'53.85%'

F1	F1				
**Classifier**	**5**	**10**	**20**	**40**	**50**
'RF'	'43.48%'	'54.55%'	'74.07%'	'56%'	'69.57%'
'PLS-DA'	'100%'	'90.32%'	'96.55%'	'88%'	'96.3%'
'SVM'	'47.62%'	'69.57%'	'57.14%'	'69.23%'	'42.11%'
'RMR'	'76.92%'	'74.07%'	'66.67%'	'68.97%'	'69.57%'
'kNN'	'64.29%'	'43.48%'	'64.29%'	'75%'	'53.33%'

Number of Generations					
**Classifier**	**5**	**10**	**20**	**40**	**50**
'RF'	35	35	35	35	35
'PLS-DA'	35	35	35	35	35
'SVM'	35	35	35	35	35
'RMR'	35	35	35	35	35
'kNN'	35	35	35	35	35

This means that in all cases the algorithm stopped after reaching the max number of generations

Genetic Algorithm: PopSize = 200, Max Num Gen = 150

Accuracy	accuracy				
**Classifier**	**5**	**10**	**20**	**40**	**50**
'RF'	'58.62%'	'58.62%'	'65.52%'	'72.41%'	'79.31%'
'PLS-DA'	'86.21%'	'89.66%'	'93.1%'	'100%'	'93.1%'
'SVM'	'72.41%'	'75.86%'	'75.86%'	'82.76%'	'55.17%'
'RMR'	'75.86%'	'68.97%'	'55.17%'	'75.86%'	'65.52%'
'kNN'	'44.83%'	'62.07%'	'65.52%'	'62.07%'	'62.07%'

Recall	recall				
**Classifier**	**5**	**10**	**20**	**40**	**50**
'RF'	'64.29%'	'35.71%'	'64.29%'	'50%'	'64.29%'
'PLS-DA'	'71.43%'	'78.57%'	'85.71%'	'100%'	'85.71%'
'SVM'	'57.14%'	'64.29%'	'64.29%'	'64.29%'	'35.71%'

'RMR'	'71.43%'	'50%'	'42.86%'	'78.57%'	'42.86%'
'kNN'	'64.29%'	'42.86%'	'57.14%'	'64.29%'	'42.86%'

Precision	precision				
**Classifier**	**5**	**10**	**20**	**40**	**50**
'RF'	'56.25%'	'62.5%'	'64.29%'	'87.5%'	'90%'
'PLS-DA'	'100%'	'100%'	'100%'	'100%'	'100%'
'SVM'	'80%'	'81.82%'	'81.82%'	'100%'	'55.56%'
'RMR'	'76.92%'	'77.78%'	'54.55%'	'73.33%'	'75%'
'kNN'	'45%'	'66.67%'	'66.67%'	'60%'	'66.67%'

Specificity	specificity				
**Classifier**	**5**	**10**	**20**	**40**	**50**
'RF'	'53.33%'	'80%'	'66.67%'	'93.33%'	'93.33%'
'PLS-DA'	'100%'	'100%'	'100%'	'100%'	'100%'
'SVM'	'86.67%'	'86.67%'	'86.67%'	'100%'	'73.33%'
'RMR'	'80%'	'86.67%'	'66.67%'	'73.33%'	'86.67%'
'kNN'	'26.67%'	'80%'	'73.33%'	'60%'	'80%'

NPV	NPV				
**Classifier**	**5**	**10**	**20**	**40**	**50**
'RF'	'61.54%'	'57.14%'	'66.67%'	'66.67%'	'73.68%'
'PLS-DA'	'78.95%'	'83.33%'	'88.24%'	'100%'	'88.24%'
'SVM'	'68.42%'	'72.22%'	'72.22%'	'75%'	'55%'
'RMR'	'75%'	'65%'	'55.56%'	'78.57%'	'61.9%'
'kNN'	'44.44%'	'60%'	'64.71%'	'64.29%'	'60%'

F1	F1				
**Classifier**	**5**	**10**	**20**	**40**	**50**
'RF'	'60%'	'45.45%'	'64.29%'	'63.64%'	'75%'
'PLS-DA'	'83.33%'	'88%'	'92.31%'	'100%'	'92.31%'
'SVM'	'66.67%'	'72%'	'72%'	'78.26%'	'43.48%'
'RMR'	'74.07%'	'60.87%'	'48%'	'75.86%'	'54.55%'
'kNN'	'52.94%'	'52.17%'	'61.54%'	'62.07%'	'52.17%'

Number of Generations					
**Classifier**	**5**	**10**	**20**	**40**	**50**
'RF'	66	69	84	108	90
'PLS-DA'	62	52	59	52	62
'SVM'	77	77	79	99	82
'RMR'	150	150	150	150	150
'kNN'	51	51	51	51	51

Rsp100_FeaturesSets_SMK_CAN_187

mRMR

Accuracy — mean accuracy ± 95% CI

Classifier	20%	30%	40%	50%	60%	70%	80%
'RF'	'68.86% ± 1.42%'	'68.69% ± 1.38%'	'68.83% ± 1.41%'	'67.9% ± 1.46%'	'68.9% ± 1.3%'	'68.34% ± 1.5%'	'68.97% ± 1.39%'
'PLS-DA'	'92.17% ± 1.22%'	'93.28% ± 1.05%'	'92.17% ± 1.09%'	'92.83% ± 1.12%'	'92.66% ± 0.99%'	'91.97% ± 1.08%'	'91.97% ± 1.06%'
'SVM'	'71.28% ± 1.41%'	'71% ± 1.33%'	'71.79% ± 1.25%'	'71.9% ± 1.27%'	'72.59% ± 1.29%'	'71.76% ± 1.28%'	'70.93% ± 1.24%'
'RLR'	'75.52% ± 1.25%'	'74.59% ± 1.26%'	'74.59% ± 1.31%'	'74.48% ± 1.42%'	'73.69% ± 1.42%'	'73.62% ± 1.4%'	'74.03% ± 1.35%'
'kNN'	'67.76% ± 1.5%'	'66.24% ± 1.42%'	'66.03% ± 1.38%'	'65.83% ± 1.37%'	'65.69% ± 1.48%'	'65.52% ± 1.53%'	'66.72% ± 1.41%'

Recall — mean recall ± 95% CI

Classifier	20%	30%	40%	50%	60%	70%	80%
'RF'	'59.79% ± 2.27%'	'59.18% ± 2.4%'	'59.1% ± 2.49%'	'58.38% ± 2.38%'	'59.92% ± 2.38%'	'59.11% ± 2.56%'	'58.45% ± 2.39%'
'PLS-DA'	'89.45% ± 2.71%'	'91.21% ± 2.17%'	'89.07% ± 2.5%'	'90.34% ± 2.4%'	'89.76% ± 2.11%'	'88.46% ± 2.7%'	'89.03% ± 2.5%'
'SVM'	'63.1% ± 2.54%'	'61.89% ± 2.37%'	'62.37% ± 2.3%'	'62.77% ± 2.56%'	'64.42% ± 2.32%'	'62.82% ± 2.53%'	'60.86% ± 2.56%'
'RLR'	'69.59% ± 2.14%'	'68.75% ± 2.24%'	'68.82% ± 2.06%'	'68.36% ± 2.16%'	'68.09% ± 2.21%'	'68.31% ± 2.21%'	'68.27% ± 2.11%'
'kNN'	'59.29% ± 2.3%'	'58.2% ± 2.47%'	'58.87% ± 2.33%'	'58.97% ± 2.37%'	'58.9% ± 2.33%'	'58.35% ± 2.32%'	'59.63% ± 2.12%'

Precision — mean precision ± 95% CI

Classifier	20%	30%	40%	50%	60%	70%	80%
'RF'	'70.21% ± 2.03%'	'69.93% ± 1.89%'	'70.56% ± 2.18%'	'69.56% ± 2.34%'	'70.22% ± 1.96%'	'69.62% ± 2.19%'	'71.31% ± 2.18%'
'PLS-DA'	'94.98% ± 1.68%'	'95.41% ± 1.56%'	'95.24% ± 1.59%'	'95.4% ± 1.59%'	'95.6% ± 1.62%'	'95.27% ± 1.45%'	'94.91% ± 1.61%'
'SVM'	'73.43% ± 2.16%'	'73.26% ± 2.08%'	'74.29% ± 1.93%'	'74.46% ± 1.96%'	'74.8% ± 2.02%'	'74.05% ± 2%'	'73.54% ± 1.85%'
'RLR'	'76.78% ± 1.8%'	'75.83% ± 1.81%'	'75.8% ± 1.93%'	'75.71% ± 2.01%'	'74.57% ± 2.08%'	'74.19% ± 1.97%'	'74.87% ± 1.93%'
'kNN'	'68.47% ± 2.09%'	'66.16% ± 1.89%'	'65.59% ± 1.78%'	'65.13% ± 1.75%'	'65.18% ± 1.95%'	'65.14% ± 1.96%'	'66.73% ± 1.86%'

Specificity — mean specificity ± 95% CI

Classifier	20%	30%	40%	50%	60%	70%	80%
'RF'	'76.84% ± 2.06%'	'77.07% ± 1.91%'	'77.37% ± 2.1%'	'76.26% ± 2.28%'	'76.82% ± 2.02%'	'76.42% ± 2.07%'	'78.22% ± 2.11%'
'PLS-DA'	'94.49% ± 1.92%'	'95.12% ± 1.76%'	'94.9% ± 1.77%'	'95.02% ± 1.82%'	'95.25% ± 1.81%'	'94.99% ± 1.6%'	'94.6% ± 1.76%'
'SVM'	'78.46% ± 2.27%'	'78.93% ± 2.08%'	'79.98% ± 1.96%'	'79.83% ± 2.09%'	'79.73% ± 2.12%'	'79.53% ± 2.04%'	'79.72% ± 2%'
'RLR'	'80.63% ± 1.92%'	'79.68% ± 2.07%'	'79.62% ± 2.05%'	'79.79% ± 2.09%'	'78.55% ± 2.12%'	'78.21% ± 2.09%'	'79.02% ± 2.02%'
'kNN'	'75.13% ± 2.17%'	'73.33% ± 2.02%'	'72.35% ± 2.06%'	'71.87% ± 2.03%'	'71.74% ± 2.15%'	'71.83% ± 2.28%'	'73.03% ± 2.21%'

NPV	mean NPV ± 95% CI						
**Classifier**	**20%**	**30%**	**40%**	**50%**	**60%**	**70%**	**80%**
'RF'	'68.85% ± 1.37%'	'68.71% ± 1.33%'	'68.82% ± 1.39%'	'67.97% ± 1.37%'	'69.11% ± 1.34%'	'68.56% ± 1.49%'	'68.6% ± 1.35%'
'PLS-DA'	'92.57% ± 1.76%'	'93.53% ± 1.52%'	'92.13% ± 1.69%'	'92.97% ± 1.63%'	'92.37% ± 1.49%'	'91.82% ± 1.82%'	'92.05% ± 1.69%'
'SVM'	'71.34% ± 1.43%'	'70.76% ± 1.35%'	'71.29% ± 1.27%'	'71.66% ± 1.36%'	'72.41% ± 1.31%'	'71.59% ± 1.34%'	'70.57% ± 1.35%'
'RLR'	'75.61% ± 1.28%'	'74.93% ± 1.36%'	'74.82% ± 1.31%'	'74.56% ± 1.39%'	'74.13% ± 1.42%'	'74.21% ± 1.4%'	'74.36% ± 1.32%'
'kNN'	'68.07% ± 1.4%'	'67.12% ± 1.4%'	'67.1% ± 1.36%'	'67.06% ± 1.35%'	'66.89% ± 1.43%'	'66.52% ± 1.46%'	'67.53% ± 1.3%'

F1	mean F1 ± 95% CI						
**Classifier**	**20%**	**30%**	**40%**	**50%**	**60%**	**70%**	**80%**
'RF'	'63.88% ± 1.76%'	'63.37% ± 1.86%'	'63.44% ± 1.87%'	'62.57% ± 1.84%'	'63.84% ± 1.71%'	'63.08% ± 1.96%'	'63.33% ± 1.81%'
'PLS-DA'	'91.02% ± 1.57%'	'92.5% ± 1.21%'	'91.07% ± 1.37%'	'91.91% ± 1.35%'	'91.8% ± 1.13%'	'90.7% ± 1.41%'	'90.87% ± 1.34%'
'SVM'	'66.8% ± 1.79%'	'66.22% ± 1.71%'	'66.99% ± 1.64%'	'67.1% ± 1.73%'	'68.37% ± 1.65%'	'67.01% ± 1.75%'	'65.62% ± 1.72%'
'RLR'	'72.38% ± 1.53%'	'71.37% ± 1.51%'	'71.48% ± 1.51%'	'71.25% ± 1.68%'	'70.53% ± 1.69%'	'70.54% ± 1.67%'	'70.85% ± 1.61%'
'kNN'	'62.87% ± 1.85%'	'61.2% ± 1.87%'	'61.4% ± 1.77%'	'61.28% ± 1.79%'	'61.23% ± 1.81%'	'60.92% ± 1.82%'	'62.33% ± 1.65%'

Relief-F

Accuracy	mean accuracy ± 95% CI						
**Classifier**	**20%**	**30%**	**40%**	**50%**	**60%**	**70%**	**80%**
'RF'	'63.88% ± 1.76%'	'63.37% ± 1.86%'	'63.44% ± 1.87%'	'62.57% ± 1.84%'	'63.84% ± 1.71%'	'63.08% ± 1.96%'	'63.33% ± 1.81%'
'PLS-DA'	'91.02% ± 1.57%'	'92.5% ± 1.21%'	'91.07% ± 1.37%'	'91.91% ± 1.35%'	'91.8% ± 1.13%'	'90.7% ± 1.41%'	'90.87% ± 1.34%'
'SVM'	'66.8% ± 1.79%'	'66.22% ± 1.71%'	'66.99% ± 1.64%'	'67.1% ± 1.73%'	'68.37% ± 1.65%'	'67.01% ± 1.75%'	'65.62% ± 1.72%'
'RLR'	'72.38% ± 1.53%'	'71.37% ± 1.51%'	'71.48% ± 1.51%'	'71.25% ± 1.68%'	'70.53% ± 1.69%'	'70.54% ± 1.67%'	'70.85% ± 1.61%'
'kNN'	'62.87% ± 1.85%'	'61.2% ± 1.87%'	'61.4% ± 1.77%'	'61.28% ± 1.79%'	'61.23% ± 1.81%'	'60.92% ± 1.82%'	'62.33% ± 1.65%'

Recall	mean recall ± 95% CI						
**Classifier**	**20%**	**30%**	**40%**	**50%**	**60%**	**70%**	**80%**
'RF'	'59.65% ± 2.4%'	'59.8% ± 2.16%'	'58.47% ± 2.4%'	'59.64% ± 2.21%'	'59.31% ± 2.4%'	'59.99% ± 2.19%'	'58.99% ± 2.3%'
'PLS-DA'	'90.08% ± 2.34%'	'90.4% ± 2.3%'	'88.48% ± 2.46%'	'89.49% ± 2.29%'	'88.17% ± 2.31%'	'87.16% ± 2.51%'	'88.79% ± 2.53%'
'SVM'	'68.69% ± 2.26%'	'66.49% ± 2.22%'	'66.1% ± 2.33%'	'64.55% ± 2.35%'	'63.49% ± 2.4%'	'63.64% ± 2.42%'	'62.39% ± 2.39%'
'RLR'	'69.46% ± 2.06%'	'68.46% ± 2.14%'	'67.58% ± 2.14%'	'67.63% ± 2.03%'	'67.76% ± 2.21%'	'67.79% ± 2.12%'	'67.92% ± 2.17%'
'kNN'	'62.73% ± 2.39%'	'62.66% ± 2.32%'	'61.97% ± 2.42%'	'63.24% ± 2.32%'	'62.14% ± 2.29%'	'60.78% ± 2.29%'	'60.94% ± 2.45%'

Precision	mean precision ± 95% CI

Classifier	20%	30%	40%	50%	60%	70%	80%
'RF'	'72.24% ± 2.33%'	'72.72% ± 1.92%'	'71.36% ± 2.11%'	'71.04% ± 2.12%'	'71.04% ± 2.06%'	'70.11% ± 1.81%'	'70.44% ± 2.19%'
'PLS-DA'	'95.3% ± 1.49%'	'95.65% ± 1.58%'	'96.46% ± 1.42%'	'96.16% ± 1.49%'	'96.4% ± 1.53%'	'96.35% ± 1.47%'	'95.2% ± 1.64%'
'SVM'	'76.75% ± 1.99%'	'76.73% ± 1.87%'	'76.29% ± 1.84%'	'75.74% ± 1.94%'	'74.93% ± 1.94%'	'74.85% ± 1.96%'	'74.55% ± 1.96%'
'RLR'	'76.02% ± 2.09%'	'75.05% ± 2.07%'	'74.64% ± 2.07%'	'74.17% ± 1.98%'	'73.91% ± 2%'	'74.55% ± 2.04%'	'73.86% ± 1.95%'
'kNN'	'69.36% ± 1.99%'	'68.19% ± 1.91%'	'67.51% ± 1.74%'	'68.44% ± 1.95%'	'66.56% ± 1.78%'	'65.71% ± 1.85%'	'64.48% ± 1.6%'

Specificity	mean specificity ± 95% CI						
**Classifier**	**20%**	**30%**	**40%**	**50%**	**60%**	**70%**	**80%**
RF'	'78.59% ± 2.18%'	'79.42% ± 1.89%'	'78.67% ± 1.97%'	'77.45% ± 2.18%'	'77.69% ± 2.14%'	'76.78% ± 1.92%'	'77.15% ± 2.17%'
'PLS-DA'	'95.01% ± 1.65%'	'95.31% ± 1.77%'	'96.19% ± 1.61%'	'95.88% ± 1.67%'	'95.97% ± 1.79%'	'96.07% ± 1.65%'	'94.76% ± 1.87%'
'SVM'	'80.57% ± 2.07%'	'81.41% ± 1.92%'	'81.07% ± 1.93%'	'80.63% ± 2.06%'	'80.26% ± 2.05%'	'79.99% ± 2.13%'	'80.04% ± 2.1%'
'RLR'	'79.25% ± 2.34%'	'78.82% ± 2.28%'	'78.69% ± 2.25%'	'78.37% ± 2.1%'	'78.08% ± 2.14%'	'78.85% ± 2.07%'	'78.15% ± 1.96%'
'kNN'	'74.67% ± 2.26%'	'73.4% ± 2.15%'	'73.02% ± 2.16%'	'73.4% ± 2.29%'	'71.99% ± 2.1%'	'71.21% ± 2.21%'	'70.03% ± 2.06%'

NPV	mean NPV ± 95% CI						
**Classifier**	**20%**	**30%**	**40%**	**50%**	**60%**	**70%**	**80%**
'RF'	'69.38% ± 1.46%'	'69.56% ± 1.29%'	'68.75% ± 1.39%'	'68.93% ± 1.3%'	'68.93% ± 1.35%'	'68.98% ± 1.24%'	'68.52% ± 1.38%'
'PLS-DA'	'92.8% ± 1.59%'	'92.98% ± 1.56%'	'91.72% ± 1.64%'	'92.31% ± 1.56%'	'91.36% ± 1.58%'	'90.69% ± 1.65%'	'91.91% ± 1.72%'
'SVM'	'75.04% ± 1.38%'	'73.89% ± 1.38%'	'73.68% ± 1.41%'	'72.68% ± 1.33%'	'72.06% ± 1.33%'	'72.03% ± 1.33%'	'71.32% ± 1.31%'
'RLR'	'75.07% ± 1.42%'	'74.37% ± 1.34%'	'73.81% ± 1.3%'	'73.72% ± 1.28%'	'73.88% ± 1.38%'	'73.99% ± 1.35%'	'74% ± 1.36%'
'kNN'	'69.95% ± 1.46%'	'69.55% ± 1.45%'	'69.12% ± 1.45%'	'69.81% ± 1.49%'	'68.78% ± 1.49%'	'67.83% ± 1.41%'	'67.67% ± 1.44%'

F1	mean F1 ± 95% CI						
**Classifier**	**20%**	**30%**	**40%**	**50%**	**60%**	**70%**	**80%**
'RF'	'64.45% ± 1.8%'	'64.91% ± 1.65%'	'63.52% ± 1.89%'	'64.02% ± 1.66%'	'63.77% ± 1.82%'	'63.99% ± 1.62%'	'63.38% ± 1.77%'
'PLS-DA'	'91.76% ± 1.26%'	'92.11% ± 1.34%'	'91.43% ± 1.38%'	'91.91% ± 1.28%'	'91.27% ± 1.29%'	'90.58% ± 1.42%'	'90.87% ± 1.38%'
'SVM'	'71.71% ± 1.69%'	'70.58% ± 1.64%'	'70.11% ± 1.68%'	'68.81% ± 1.62%'	'67.86% ± 1.67%'	'67.84% ± 1.66%'	'66.99% ± 1.65%'
'RLR'	'71.86% ± 1.48%'	'70.92% ± 1.63%'	'70.27% ± 1.66%'	'70.2% ± 1.59%'	'70.08% ± 1.66%'	'70.46% ± 1.68%'	'70.2% ± 1.64%'
'kNN'	'65.14% ± 1.81%'	'64.61% ± 1.7%'	'63.89% ± 1.71%'	'65.05% ± 1.75%'	'63.71% ± 1.74%'	'62.44% ± 1.65%'	'62.01% ± 1.73%'

Chi-Squared

Accuracy	mean accuracy ± 95% CI						
**Classifier**	**20%**	**30%**	**40%**	**50%**	**60%**	**70%**	**80%**
'RF'	'70.34% ± 1.48%'	'70.21% ± 1.25%'	'69.97% ± 1.29%'	'69.48% ± 1.26%'	'68.69% ± 1.36%'	'69.38% ± 1.38%'	'68.48% ± 1.36%'
'PLS-DA'	'92.52% ± 1.01%'	'91.97% ± 0.99%'	'92.38% ± 1.01%'	'92.55% ± 1.02%'	'92.86% ± 1.06%'	'92.07% ± 1.11%'	'92.41% ± 1.11%'
'SVM'	'74.97% ± 1.28%'	'74.48% ± 1.24%'	'74.93% ± 1.27%'	'74.17% ± 1.31%'	'74.24% ± 1.28%'	'73.52% ± 1.32%'	'72.97% ± 1.11%'
'RLR'	'72.52% ± 1.46%'	'73.38% ± 1.23%'	'73.62% ± 1.42%'	'73.59% ± 1.37%'	'73.62% ± 1.33%'	'73.41% ± 1.5%'	'73.41% ± 1.42%'
'kNN'	'66.69% ± 1.49%'	'66.34% ± 1.45%'	'66.14% ± 1.46%'	'65.41% ± 1.39%'	'65.52% ± 1.35%'	'66.45% ± 1.41%'	'65.21% ± 1.48%'

Recall	mean recall ± 95% CI						
**Classifier**	**20%**	**30%**	**40%**	**50%**	**60%**	**70%**	**80%**
'RF'	'61.16% ± 2.33%'	'60.14% ± 2.23%'	'59.92% ± 2.32%'	'60.46% ± 2.16%'	'59.9% ± 2.28%'	'59.22% ± 2.27%'	'58.97% ± 2.34%'
'PLS-DA'	'90.26% ± 2.29%'	'89.81% ± 2.34%'	'89.5% ± 2.36%'	'89.76% ± 2.42%'	'90.4% ± 2.32%'	'88.36% ± 2.56%'	'89.48% ± 2.42%'
'SVM'	'69.7% ± 2.2%'	'68.8% ± 2.18%'	'68.26% ± 2.36%'	'67.94% ± 2.29%'	'66.24% ± 2.39%'	'66.07% ± 2.45%'	'65.27% ± 2.31%'
'RLR'	'67.44% ± 2.29%'	'68.23% ± 2.1%'	'68.19% ± 2.16%'	'68.04% ± 2.23%'	'67.98% ± 2.18%'	'68.11% ± 2.29%'	'67.01% ± 2.23%'
'kNN'	'60.42% ± 2.33%'	'61.85% ± 2.28%'	'61.1% ± 2.24%'	'60.32% ± 2.34%'	'59.99% ± 2.26%'	'60.5% ± 2.35%'	'59.93% ± 2.3%'

Precision	mean precision ± 95% CI						
**Classifier**	**20%**	**30%**	**40%**	**50%**	**60%**	**70%**	**80%**
'RF'	'72.37% ± 2.35%'	'72.76% ± 2.08%'	'72.16% ± 1.96%'	'71.35% ± 2.06%'	'70.07% ± 2.04%'	'71.73% ± 2.1%'	'70.1% ± 2.1%'
'PLS-DA'	'94.88% ± 1.59%'	'94.27% ± 1.64%'	'95.24% ± 1.55%'	'95.29% ± 1.48%'	'95.38% ± 1.58%'	'95.68% ± 1.54%'	'95.39% ± 1.57%'
'SVM'	'76.02% ± 1.92%'	'75.73% ± 1.94%'	'76.76% ± 1.92%'	'75.25% ± 1.79%'	'76.66% ± 1.96%'	'75.62% ± 2.06%'	'75.03% ± 1.81%'
'RLR'	'72.83% ± 2.01%'	'73.97% ± 1.83%'	'74.13% ± 2.05%'	'73.91% ± 1.83%'	'74.2% ± 1.94%'	'73.84% ± 2.14%'	'74.35% ± 2.06%'
'kNN'	'66.41% ± 1.98%'	'65.49% ± 1.98%'	'65.43% ± 1.98%'	'64.34% ± 1.82%'	'64.46% ± 1.74%'	'65.75% ± 1.78%'	'64.36% ± 1.96%'

Specificity	mean specificity ± 95% CI						
**Classifier**	**20%**	**30%**	**40%**	**50%**	**60%**	**70%**	**80%**
'RF'	'78.44% ± 2.16%'	'79.06% ± 2.01%'	'78.81% ± 1.93%'	'77.45% ± 2.14%'	'76.45% ± 2.1%'	'78.35% ± 2.12%'	'76.82% ± 2.15%'
'PLS-DA'	'94.49% ± 1.79%'	'93.86% ± 1.86%'	'94.86% ± 1.73%'	'95% ± 1.61%'	'95.03% ± 1.75%'	'95.35% ± 1.74%'	'94.9% ± 1.88%'
'SVM'	'79.52% ± 2.08%'	'79.41% ± 2.1%'	'80.77% ± 2.02%'	'79.57% ± 1.85%'	'81.22% ± 1.95%'	'80% ± 2.08%'	'79.65% ± 1.95%'
'RLR'	'76.84% ± 2.19%'	'77.83% ± 2.05%'	'78.3% ± 1.97%'	'78.35% ± 1.8%'	'78.51% ± 1.93%'	'77.97% ± 2.13%'	'78.97% ± 1.99%'
'kNN'	'72.16% ± 2.27%'	'70.32% ± 2.34%'	'70.54% ± 2.35%'	'69.85% ± 2.21%'	'70.4% ± 2.02%'	'71.64% ± 2.16%'	'69.92% ± 2.36%'

NPV	mean NPV ± 95% CI						
**Classifier**	**20%**	**30%**	**40%**	**50%**	**60%**	**70%**	**80%**
'RF'	'70.11% ± 1.37%'	'69.74% ± 1.23%'	'69.58% ± 1.29%'	'69.37% ± 1.18%'	'68.85% ± 1.34%'	'68.97% ± 1.3%'	'68.49% ± 1.32%'
'PLS-DA'	'92.87% ± 1.59%'	'92.6% ± 1.65%'	'92.39% ± 1.65%'	'92.55% ± 1.65%'	'92.99% ± 1.58%'	'91.59% ± 1.71%'	'92.42% ± 1.67%'
'SVM'	'75.5% ± 1.35%'	'74.92% ± 1.25%'	'74.99% ± 1.36%'	'74.46% ± 1.41%'	'73.88% ± 1.36%'	'73.55% ± 1.39%'	'72.96% ± 1.26%'
'RLR'	'73.38% ± 1.43%'	'74.12% ± 1.29%'	'74.17% ± 1.39%'	'74.16% ± 1.42%'	'74.13% ± 1.35%'	'74.05% ± 1.43%'	'73.62% ± 1.36%'
'kNN'	'67.81% ± 1.41%'	'68.04% ± 1.41%'	'67.63% ± 1.42%'	'67.14% ± 1.38%'	'67.1% ± 1.34%'	'67.78% ± 1.4%'	'66.83% ± 1.42%'

F1	mean F1 ± 95% CI						
**Classifier**	**20%**	**30%**	**40%**	**50%**	**60%**	**70%**	**80%**
'RF'	'70.11% ± 1.37%'	'69.74% ± 1.23%'	'69.58% ± 1.29%'	'69.37% ± 1.18%'	'68.85% ± 1.34%'	'68.97% ± 1.3%'	'68.49% ± 1.32%'
'PLS-DA'	'92.87% ± 1.59%'	'92.6% ± 1.65%'	'92.39% ± 1.65%'	'92.55% ± 1.65%'	'92.99% ± 1.58%'	'91.59% ± 1.71%'	'92.42% ± 1.67%'
'SVM'	'75.5% ± 1.35%'	'74.92% ± 1.25%'	'74.99% ± 1.36%'	'74.46% ± 1.41%'	'73.88% ± 1.36%'	'73.55% ± 1.39%'	'72.96% ± 1.26%'
'RLR'	'73.38% ± 1.43%'	'74.12% ± 1.29%'	'74.17% ± 1.39%'	'74.16% ± 1.42%'	'74.13% ± 1.35%'	'74.05% ± 1.43%'	'73.62% ± 1.36%'
'kNN'	'67.81% ± 1.41%'	'68.04% ± 1.41%'	'67.63% ± 1.42%'	'67.14% ± 1.38%'	'67.1% ± 1.34%'	'67.78% ± 1.4%'	'66.83% ± 1.42%'

Variance

Accuracy	mean accuracy ± 95% CI						
**Classifier**	**20%**	**30%**	**40%**	**50%**	**60%**	**70%**	**80%**
'RF'	'73% ± 1.18%'	'71.07% ± 1.33%'	'70.93% ± 1.38%'	'70.14% ± 1.36%'	'69.28% ± 1.3%'	'69% ± 1.33%'	'69.34% ± 1.23%'
'PLS-DA'	'92.97% ± 1.07%'	'92.34% ± 1.09%'	'93.14% ± 1.08%'	'92.38% ± 1.09%'	'92.62% ± 1.07%'	'92.59% ± 1.26%'	'92.83% ± 1.11%'
'SVM'	'71.52% ± 1.28%'	'72.38% ± 1.32%'	'73.48% ± 1.31%'	'73.14% ± 1.27%'	'72.21% ± 1.3%'	'72.14% ± 1.28%'	'72.28% ± 1.19%'
'RLR'	'71.9% ± 1.44%'	'73.31% ± 1.36%'	'74.17% ± 1.4%'	'72.9% ± 1.51%'	'72.69% ± 1.55%'	'72.48% ± 1.53%'	'72.24% ± 1.51%'
'kNN'	'67.62% ± 1.69%'	'66.93% ± 1.51%'	'67.45% ± 1.53%'	'66.24% ± 1.52%'	'66.21% ± 1.54%'	'66.66% ± 1.56%'	'65.03% ± 1.4%'

Recall	mean recall ± 95% CI						
**Classifier**	**20%**	**30%**	**40%**	**50%**	**60%**	**70%**	**80%**
'RF'	'62.96% ± 2.1%'	'62.01% ± 2.29%'	'60.84% ± 2.35%'	'60.81% ± 2.13%'	'59.92% ± 2.09%'	'60.48% ± 2.17%'	'59.58% ± 2.31%'
'PLS-DA'	'90.91% ± 2.44%'	'90.99% ± 2.44%'	'89.48% ± 2.32%'	'88.33% ± 2.61%'	'90.1% ± 2.5%'	'89.91% ± 2.61%'	'90.31% ± 2.37%'
'SVM'	'62.06% ± 2.41%'	'63.18% ± 2.53%'	'65.8% ± 2.48%'	'65.12% ± 2.42%'	'63.2% ± 2.45%'	'62.92% ± 2.44%'	'63.13% ± 2.38%'
'RLR'	'62.58% ± 2.05%'	'64.63% ± 2.33%'	'66.87% ± 2.21%'	'65% ± 2.23%'	'65.58% ± 2.44%'	'66.47% ± 2.23%'	'65.69% ± 2.29%'
'kNN'	'61.81% ± 2.69%'	'60.46% ± 2.44%'	'61.7% ± 2.23%'	'60.01% ± 2.41%'	'60.09% ± 2.33%'	'60.27% ± 2.36%'	'59.3% ± 2.25%'

Precision	mean precision ± 95% CI					
**Classifier**	**20%**	**30%**	**40%**	**50%**	**60%**	**70%**	**80%**
'RF'	'76.06% ± 1.84%'	'72.95% ± 1.99%'	'73.46% ± 2%'	'72.17% ± 2.09%'	'71.12% ± 2.05%'	'70.33% ± 1.87%'	'71.5% ± 1.97%'
'PLS-DA'	'94.97% ± 1.52%'	'93.93% ± 1.68%'	'96.58% ± 1.4%'	'96.08% ± 1.4%'	'95.06% ± 1.52%'	'95.43% ± 1.63%'	'95.4% ± 1.6%'
'SVM'	'74.27% ± 2.03%'	'75.05% ± 2.03%'	'75.27% ± 1.82%'	'74.96% ± 1.76%'	'74.3% ± 1.84%'	'74.45% ± 1.8%'	'74.62% ± 1.73%'
'RLR'	'74.44% ± 2.1%'	'76.04% ± 2.01%'	'76.1% ± 2%'	'74.83% ± 2.13%'	'73.85% ± 2.08%'	'73.15% ± 2.08%'	'73.31% ± 2.18%'
'kNN'	'67.12% ± 2.07%'	'66.37% ± 1.88%'	'67.03% ± 1.96%'	'65.51% ± 1.96%'	'65.61% ± 1.98%'	'66.24% ± 2.09%'	'64.17% ± 1.86%'

Specificity	mean specificity ± 95% CI						
**Classifier**	**20%**	**30%**	**40%**	**50%**	**60%**	**70%**	**80%**
'RF'	'81.82% ± 1.7%'	'79.09% ± 1.84%'	'79.83% ± 1.92%'	'78.31% ± 2.08%'	'77.48% ± 2%'	'76.49% ± 2.12%'	'77.96% ± 2.04%'
'PLS-DA'	'94.71% ± 1.66%'	'93.59% ± 1.86%'	'96.32% ± 1.58%'	'95.86% ± 1.52%'	'94.87% ± 1.63%'	'94.94% ± 1.95%'	'95.05% ± 1.79%'
'SVM'	'79.77% ± 2.1%'	'80.34% ± 2.03%'	'80.15% ± 1.89%'	'80.1% ± 1.87%'	'80.07% ± 1.86%'	'80.17% ± 1.91%'	'80.25% ± 1.87%'
'RLR'	'80.03% ± 2.08%'	'80.85% ± 2.18%'	'80.5% ± 2.12%'	'79.83% ± 2.13%'	'78.93% ± 2.08%'	'77.67% ± 2.24%'	'77.93% ± 2.25%'
'kNN'	'72.76% ± 2.22%'	'72.65% ± 2%'	'72.48% ± 2.2%'	'71.79% ± 2.09%'	'71.62% ± 2.18%'	'72.28% ± 2.2%'	'70.17% ± 2.16%'

NPV	mean NPV ± 95% CI						
**Classifier**	**20%**	**30%**	**40%**	**50%**	**60%**	**70%**	**80%**
'RF'	'71.96% ± 1.2%'	'70.79% ± 1.36%'	'70.32% ± 1.37%'	'69.78% ± 1.26%'	'69.06% ± 1.24%'	'69.08% ± 1.3%'	'69.2% ± 1.31%'
'PLS-DA'	'93.52% ± 1.61%'	'93.52% ± 1.67%'	'92.34% ± 1.56%'	'91.7% ± 1.69%'	'92.86% ± 1.66%'	'92.79% ± 1.77%'	'92.97% ± 1.61%'
'SVM'	'71.07% ± 1.32%'	'71.94% ± 1.37%'	'73.39% ± 1.44%'	'72.99% ± 1.36%'	'71.88% ± 1.36%'	'71.7% ± 1.37%'	'71.86% ± 1.29%'
'RLR'	'71.12% ± 1.32%'	'72.73% ± 1.39%'	'73.87% ± 1.37%'	'72.51% ± 1.42%'	'72.81% ± 1.55%'	'72.93% ± 1.41%'	'72.58% ± 1.43%'
'kNN'	'69.03% ± 1.73%'	'68.14% ± 1.5%'	'68.58% ± 1.47%'	'67.54% ± 1.46%'	'67.48% ± 1.5%'	'67.82% ± 1.46%'	'66.58% ± 1.35%'

F1	mean F1 ± 95% CI						
**Classifier**	**20%**	**30%**	**40%**	**50%**	**60%**	**70%**	**80%**
'RF'	'68.23% ± 1.54%'	'66.31% ± 1.72%'	'65.76% ± 1.8%'	'65.29% ± 1.67%'	'64.29% ± 1.59%'	'64.28% ± 1.59%'	'64.09% ± 1.56%'
'PLS-DA'	'92% ± 1.36%'	'91.46% ± 1.31%'	'92.11% ± 1.34%'	'91.07% ± 1.5%'	'91.56% ± 1.41%'	'91.58% ± 1.51%'	'91.9% ± 1.34%'
'SVM'	'66.62% ± 1.69%'	'67.61% ± 1.82%'	'69.4% ± 1.77%'	'68.92% ± 1.71%'	'67.52% ± 1.75%'	'67.38% ± 1.72%'	'67.56% ± 1.62%'
'RLR'	'67.37% ± 1.66%'	'69.04% ± 1.67%'	'70.51% ± 1.67%'	'68.9% ± 1.79%'	'68.82% ± 1.92%'	'69.08% ± 1.79%'	'68.61% ± 1.79%'
'kNN'	'63.63% ± 2.06%'	'62.67% ± 1.88%'	'63.67% ± 1.77%'	'62.01% ± 1.92%'	'62.11% ± 1.84%'	'62.47% ± 1.91%'	'60.98% ± 1.69%'

Rsp100_Biomarkers_GLI_85

mRMR

Accuracy — mean accuracy ± 95% CI

Classifier	5	10	20	40	50
'RF'	'83.38% ± 2.04%'	'85.54% ± 1.85%'	'87% ± 1.63%'	'87.92% ± 1.59%'	'87.62% ± 1.56%'
'PLS-DA'	'87.08% ± 2.1%'	'84.92% ± 2.23%'	'83% ± 2.04%'	'83.54% ± 2.09%'	'82.77% ± 1.92%'
'SVM'	'84.23% ± 1.82%'	'85.62% ± 1.91%'	'86.08% ± 1.68%'	'86.31% ± 1.74%'	'87.46% ± 1.6%'
'RLR'	'83.23% ± 1.96%'	'83.46% ± 1.97%'	'83.54% ± 1.96%'	'84.92% ± 1.9%'	'84.31% ± 1.83%'
'kNN'	'83.38% ± 1.81%'	'85.69% ± 1.69%'	'86.92% ± 1.58%'	'88.69% ± 1.69%'	'88.15% ± 1.87%'

Recall — mean recall ± 95% CI

Classifier	5	10	20	40	50
'RF'	'68% ± 4.97%'	'69.08% ± 4.62%'	'70.5% ± 4.47%'	'67.58% ± 4.68%'	'67.42% ± 4.68%'
'PLS-DA'	'99.5% ± 0.69%'	'99.33% ± 0.92%'	'99.5% ± 0.69%'	'99.25% ± 0.84%'	'98.67% ± 1.35%'
'SVM'	'63.67% ± 4.58%'	'67.92% ± 5.15%'	'71.83% ± 4.34%'	'69.33% ± 5.05%'	'68.75% ± 4.78%'
'RLR'	'62% ± 5.03%'	'66.83% ± 5.02%'	'68.92% ± 4.55%'	'66.58% ± 4.81%'	'66.33% ± 4.71%'
'kNN'	'64% ± 4.8%'	'71.42% ± 4.14%'	'75.33% ± 4.09%'	'73.92% ± 4.77%'	'74.5% ± 4.78%'

Precision — mean precision ± 95% CI

Classifier	5	10	20	40	50
'RF'	'72.84% ± 4.53%'	'77.73% ± 4.05%'	'82.1% ± 3.84%'	'87.19% ± 3.61%'	'85.75% ± 3.86%'
'PLS-DA'	'72.31% ± 3.73%'	'69.05% ± 3.76%'	'65.04% ± 3.35%'	'66.58% ± 3.65%'	'64.87% ± 3.26%'
'SVM'	'77.34% ± 4.34%'	'78.81% ± 4.3%'	'78.38% ± 4.14%'	'78.97% ± 4.31%'	'84.18% ± 3.88%'
'RLR'	'75.28% ± 4.77%'	'73.75% ± 4.46%'	'72.99% ± 4.27%'	'77.47% ± 4.48%'	'76.62% ± 4.49%'
'kNN'	'73.43% ± 4.28%'	'77.63% ± 3.94%'	'79.48% ± 3.81%'	'84.3% ± 4.1%'	'82.58% ± 4.25%'

Specificity — mean specificity ± 95% CI

Classifier	5	10	20	40	50
'RF'	'89.09% ± 2.14%'	'91.7% ± 1.64%'	'93.18% ± 1.57%'	'95.51% ± 1.3%'	'95.06% ± 1.32%'
'PLS-DA'	'82.5% ± 2.95%'	'79.54% ± 3.09%'	'76.9% ± 2.88%'	'77.73% ± 2.95%'	'76.88% ± 2.79%'
'SVM'	'91.73% ± 1.81%'	'92.21% ± 1.7%'	'91.39% ± 1.64%'	'92.51% ± 1.47%'	'94.46% ± 1.43%'
'RLR'	'90.93% ± 1.95%'	'89.67% ± 1.97%'	'89.18% ± 2.02%'	'91.78% ± 1.74%'	'91.08% ± 1.84%'
'kNN'	'90.34% ± 1.68%'	'91.16% ± 1.75%'	'91.32% ± 1.7%'	'94.1% ± 1.5%'	'93.29% ± 1.66%'

NPV — mean NPV ± 95% CI

Classifier	5	10	20	40	50
'RF'	'88.93% ± 1.68%'	'89.31% ± 1.59%'	'90.04% ± 1.52%'	'89.29% ± 1.46%'	'89.32% ± 1.48%'
'PLS-DA'	'99.8% ± 0.28%'	'99.82% ± 0.25%'	'99.8% ± 0.28%'	'99.7% ± 0.34%'	'99.53% ± 0.48%'
'SVM'	'87.8% ± 1.48%'	'89.14% ± 1.65%'	'90.28% ± 1.5%'	'89.79% ± 1.62%'	'89.71% ± 1.55%'
'RLR'	'87.3% ± 1.63%'	'88.51% ± 1.69%'	'88.9% ± 1.62%'	'88.6% ± 1.64%'	'88.44% ± 1.6%'
'kNN'	'87.93% ± 1.57%'	'89.99% ± 1.44%'	'91.37% ± 1.4%'	'91.36% ± 1.56%'	'91.28% ± 1.59%'

F1 — mean F1 ± 95% CI

Classifier	5	10	20	40	50

'RF'	'68.14% ± 3.86%'	'70.85% ± 3.75%'	'73.27% ± 3.43%'	'73.97% ± 3.61%'	'73.45% ± 3.46%'
'PLS-DA'	'82.24% ± 2.47%'	'79.79% ± 2.56%'	'77.3% ± 2.29%'	'78.09% ± 2.43%'	'76.7% ± 2.14%'
'SVM'	'67.65% ± 3.67%'	'72.57% ± 3.67%'	'73.31% ± 3.16%'	'73.24% ± 3.5%'	'74.26% ± 3.36%'
'RLR'	'66.74% ± 3.82%'	'68.97% ± 3.63%'	'69.77% ± 3.36%'	'69.72% ± 3.82%'	'68.87% ± 3.57%'
'kNN'	'65.81% ± 3.86%'	'72.07% ± 3.26%'	'74.92% ± 3.11%'	'77.83% ± 3.24%'	'76.72% ± 3.71%'

Relief-F

Accuracy — mean accuracy ± 95% CI

Classifier	5	10	20	40	50
'RF'	'86.85% ± 1.78%'	'89.15% ± 1.65%'	'89.85% ± 1.51%'	'90.08% ± 1.44%'	'89.85% ± 1.64%'
'PLS-DA'	'92.77% ± 1.4%'	'91.62% ± 1.53%'	'88.85% ± 1.73%'	'86.31% ± 1.85%'	'85.85% ± 1.97%'
'SVM'	'85.85% ± 1.71%'	'87.62% ± 1.75%'	'88.77% ± 1.58%'	'90.38% ± 1.43%'	'90.23% ± 1.64%'
'RLR'	'84.69% ± 1.69%'	'88.31% ± 1.73%'	'87.15% ± 1.63%'	'87.38% ± 1.73%'	'87.23% ± 1.68%'
'kNN'	'87.46% ± 1.46%'	'89.23% ± 1.56%'	'89.31% ± 1.63%'	'91.08% ± 1.64%'	'90.69% ± 1.59%'

Recall — mean recall ± 95% CI

Classifier	5	10	20	40	50
'RF'	'73% ± 4.22%'	'73.33% ± 4.51%'	74.5% ± 4.37%'	'74.33% ± 4.18%'	'75.5% ± 4.54%'
'PLS-DA'	'93.58% ± 2.68%'	'98% ± 1.45%'	'100% ± 0%'	'99.08% ± 1.04%'	'98.92% ± 1.06%'
'SVM'	'65.67% ± 5.25%'	'71.58% ± 5.03%'	'73.58% ± 4.34%'	'77.25% ± 4.2%'	'76.08% ± 4.43%'
'RLR'	'63.58% ± 4.93%'	'75.17% ± 4.76%'	'73.92% ± 4.64%'	'74.17% ± 4.8%'	'72% ± 4.77%'
'kNN'	'71.33% ± 4.53%'	'73.58% ± 4.58%'	'70.83% ± 4.63%'	'75.67% ± 4.39%'	'75% ± 4.61%'

Precision — mean precision ± 95% CI

Classifier	5	10	20	40	50
'RF'	'80.52% ± 4%'	'86.32% ± 3.64%'	'87.3% ± 3.26%'	'88.03% ± 3.28%'	'86.45% ± 3.56%'
'PLS-DA'	'86.39% ± 3.25%'	'80.78% ± 3.28%'	'73.92% ± 3.31%'	'70.4% ± 3.44%'	'69.58% ± 3.43%'
'SVM'	'81.94% ± 3.99%'	'81.45% ± 4.03%'	'84.82% ± 3.66%'	'87.65% ± 3.17%'	'87.7% ± 3.46%'
'RLR'	'80.91% ± 3.98%'	'81.75% ± 3.88%'	'79.19% ± 3.78%'	'80.56% ± 3.85%'	'81.08% ± 4.03%'
'kNN'	'82.32% ± 3.6%'	'86.41% ± 3.45%'	'88.42% ± 3.47%'	'90.5% ± 3.3%'	'89.81% ± 3.21%'

Specificity — mean specificity ± 95% CI

Classifier	5	10	20	40	50
'RF'	'91.98% ± 1.81%'	'95.08% ± 1.32%'	'95.48% ± 1.14%'	'95.93% ± 1.08%'	'95.17% ± 1.25%'
'PLS-DA'	'92.42% ± 2.04%'	'89.28% ± 2.17%'	'84.81% ± 2.31%'	'81.53% ± 2.61%'	'81.16% ± 2.75%'
'SVM'	'93.22% ± 1.61%'	'93.52% ± 1.42%'	'94.46% ± 1.35%'	'95.29% ± 1.21%'	'95.49% ± 1.34%'
'RLR'	'92.51% ± 1.74%'	'93.21% ± 1.5%'	'91.94% ± 1.53%'	'92.37% ± 1.58%'	'92.99% ± 1.45%'
'kNN'	'93.4% ± 1.35%'	'95.07% ± 1.29%'	'96.2% ± 1.13%'	'96.83% ± 1.09%'	'96.64% ± 1.05%'

NPV — mean NPV ± 95% CI

Classifier	5	10	20	40	50
'RF'	'90.56% ± 1.48%'	'90.99% ± 1.48%'	'91.54% ± 1.45%'	'91.45% ± 1.39%'	'91.79% ± 1.5%'
'PLS-DA'	'97.93% ± 0.84%'	'99.33% ± 0.48%'	'100% ± 0%'	'99.72% ± 0.32%'	'99.61% ± 0.38%'
'SVM'	'88.81% ± 1.68%'	'90.6% ± 1.64%'	'91.04% ± 1.43%'	'92.33% ± 1.38%'	'91.99% ± 1.49%'
'RLR'	'87.95% ± 1.61%'	'91.64% ± 1.59%'	'91.18% ± 1.47%'	'91.21% ± 1.61%'	'90.5% ± 1.58%'

'kNN'	'90.47% ± 1.47%'	'91.13% ± 1.46%'	'90.29% ± 1.51%'	'91.82% ± 1.47%'	'91.61% ± 1.51%'

F1 mean F1 ± 95% CI

Classifier	5	10	20	40	50
'RF'	'74.36% ± 3.38%'	'77.14% ± 3.63%'	'78.36% ± 3.42%'	'78.84% ± 3.28%'	'78.64% ± 3.67%'
'PLS-DA'	'87.87% ± 2.14%'	'87.21% ± 2.03%'	'83.92% ± 2.23%'	'80.88% ± 2.25%'	'80.19% ± 2.28%'
'SVM'	'70.43% ± 3.74%'	'73.82% ± 3.99%'	'76.71% ± 3.48%'	'80% ± 3.17%'	'79.53% ± 3.52%'
'RLR'	'67.81% ± 3.54%'	'76.12% ± 3.78%'	'74.7% ± 3.44%'	'74.52% ± 3.66%'	'74.36% ± 3.46%'
'kNN'	'73.75% ± 3.41%'	'77.81% ± 3.45%'	'76.6% ± 3.74%'	'80.8% ± 3.63%'	'80.46% ± 3.51%'

Chi-Squared

Accuracy mean accuracy ± 95% CI

Classifier	5	10	20	40	50
'RF'	'74.36% ± 3.38%'	'77.14% ± 3.63%'	'78.36% ± 3.42%'	'78.84% ± 3.28%'	'78.64% ± 3.67%'
'PLS-DA'	'87.87% ± 2.14%'	'87.21% ± 2.03%'	'83.92% ± 2.23%'	'80.88% ± 2.25%'	'80.19% ± 2.28%'
'SVM'	'70.43% ± 3.74%'	'73.82% ± 3.99%'	'76.71% ± 3.48%'	'80% ± 3.17%'	'79.53% ± 3.52%'
'RLR'	'67.81% ± 3.54%'	'76.12% ± 3.78%'	'74.7% ± 3.44%'	'74.52% ± 3.66%'	'74.36% ± 3.46%'
'kNN'	'73.75% ± 3.41%'	'77.81% ± 3.45%'	'76.6% ± 3.74%'	'80.8% ± 3.63%'	'80.46% ± 3.51%'

Recall mean recall ± 95% CI

Classifier	5	10	20	40	50
'RF'	'77.5% ± 4.26%'	'78.75% ± 4.43%'	'78.5% ± 4.22%'	'77.42% ± 4.34%'	'76.42% ± 4.28%'
'PLS-DA'	'99.33% ± 0.92%'	'98.83% ± 1.14%'	'99.17% ± 0.94%'	'98.08% ± 1.55%'	'97.58% ± 1.88%'
'SVM'	'66.58% ± 4.92%'	'70.92% ± 4.77%'	'75.33% ± 4.69%'	'73.67% ± 4.81%'	'73.5% ± 4.52%'
'RLR'	'67.5% ± 5.01%'	'74.17% ± 4.66%'	'76.92% ± 4.36%'	'76.33% ± 4.23%'	'74.42% ± 4.42%'
'kNN'	'71.17% ± 5.1%'	'73.42% ± 4.48%'	'73.58% ± 4.75%'	'75.67% ± 4.64%'	'74.67% ± 4.83%'

Precision mean precision ± 95% CI

Classifier	5	10	20	40	50
'RF'	'80.81% ± 3.66%'	'84.71% ± 3.43%'	'85.99% ± 3.47%'	'87.98% ± 3.18%'	'88.53% ± 3.32%'
'PLS-DA'	'67.82% ± 3.8%'	'66.63% ± 3.35%'	'68.96% ± 3.44%'	'70.22% ± 3.64%'	'70.13% ± 3.37%'
'SVM'	'77.49% ± 4.18%'	'84.37% ± 3.89%'	'85.15% ± 3.72%'	'88.17% ± 3.39%'	'90.15% ± 3.16%'
'RLR'	'73.23% ± 3.87%'	'78.39% ± 4.06%'	'83.03% ± 3.86%'	'82.64% ± 3.88%'	'81.79% ± 3.97%'
'kNN'	'75.55% ± 4.41%'	'81.52% ± 3.99%'	'87% ± 3.48%'	'87.88% ± 3.27%'	'87.72% ± 3.4%'

Specificity mean specificity ± 95% CI

Classifier	5	10	20	40	50
RF'	'91.99% ± 1.67%'	'93.68% ± 1.51%'	'94.31% ± 1.45%'	'95.36% ± 1.25%'	'95.79% ± 1.21%'
'PLS-DA'	'78.58% ± 3.31%'	'78.68% ± 2.82%'	'80.12% ± 2.87%'	'81.19% ± 2.84%'	'81.17% ± 2.88%'
'SVM'	'91.52% ± 1.84%'	'94.39% ± 1.42%'	'94.71% ± 1.28%'	'95.59% ± 1.35%'	'96.63% ± 1.05%'
'RLR'	'89.47% ± 1.88%'	'91.14% ± 1.75%'	'93.39% ± 1.47%'	'92.88% ± 1.61%'	'92.52% ± 1.71%'
'kNN'	'91.09% ± 1.58%'	'92.64% ± 1.7%'	'95.34% ± 1.2%'	'95.47% ± 1.22%'	'95.69% ± 1.22%'

NPV mean NPV ± 95% CI

Classifier	5	10	20	40	50

'RF'	'92.13% ± 1.41%'	'92.58% ± 1.49%'	'92.63% ± 1.43%'	'92.41% ± 1.38%'	'92.19% ± 1.41%'
'PLS-DA'	'99.82% ± 0.25%'	'99.62% ± 0.37%'	'99.71% ± 0.33%'	'99.34% ± 0.54%'	'99.26% ± 0.55%'
'SVM'	'88.71% ± 1.54%'	'90.29% ± 1.58%'	'91.86% ± 1.51%'	'91.37% ± 1.51%'	'91.36% ± 1.43%'
'RLR'	'88.81% ± 1.63%'	'91.07% ± 1.58%'	'92.16% ± 1.45%'	'91.94% ± 1.45%'	'91.3% ± 1.5%'
'kNN'	'90.27% ± 1.62%'	'90.92% ± 1.51%'	'91.25% ± 1.51%'	'91.95% ± 1.44%'	'91.6% ± 1.54%'

F1 mean F1 ± 95% CI

Classifier	5	10	20	40	50
'RF'	'76.87% ± 3.42%'	'79.92% ± 3.24%'	'79.99% ± 3.37%'	'80.97% ± 3.13%'	'80.15% ± 3.41%'
'PLS-DA'	'78.87% ± 2.62%'	'78.07% ± 2.2%'	'79.91% ± 2.28%'	'79.98% ± 2.33%'	'79.74% ± 2.17%'
'SVM'	'69.69% ± 3.66%'	'75.29% ± 3.52%'	'78.65% ± 3.43%'	'78.26% ± 3.57%'	'79.53% ± 3.41%'
'RLR'	'67.58% ± 3.63%'	'74.25% ± 3.24%'	'79.23% ± 2.99%'	'77.77% ± 3%'	'75.53% ± 3.58%'
'kNN'	'72.41% ± 3.69%'	'75.49% ± 3.18%'	'77.97% ± 3.4%'	'79.58% ± 3.43%'	'79.72% ± 3.47%'

Variance

Accuracy mean accuracy ± 95% CI

Classifier	5	10	20	40	50
'RF'	'64.46% ± 2.5%'	'71.92% ± 3.1%'	'81.15% ± 1.92%'	'82.92% ± 1.85%'	'82.08% ± 1.98%'
'PLS-DA'	'85.77% ± 2.16%'	'85.46% ± 1.97%'	'81% ± 2.28%'	'76.08% ± 2.39%'	'79% ± 2.27%'
'SVM'	'71.92% ± 1.18%'	'72.77% ± 2.28%'	'76.46% ± 2.52%'	'86.08% ± 2.18%'	'85.77% ± 2.15%'
'RLR'	'67.15% ± 1.82%'	'70.23% ± 1.99%'	'78.69% ± 2.11%'	'87.08% ± 2.05%'	'87.77% ± 1.88%'
'kNN'	'67.62% ± 2.41%'	'67.23% ± 2.18%'	'78.08% ± 2.25%'	'81.08% ± 2.33%'	'81.38% ± 2.07%'

Recall mean recall ± 95% CI

Classifier	5	10	20	40	50
'RF'	'42.75% ± 5.97%'	'50.92% ± 5.73%'	'61.42% ± 5.05%'	'60.25% ± 5.38%'	'59.33% ± 5.58%'
'PLS-DA'	'95.92% ± 1.94%'	'98.42% ± 1.24%'	'98.42% ± 1.24%'	'99.75% ± 0.49%'	'100% ± 0%'
'SVM'	'0.58% ± 0.81%'	'46.75% ± 5.67%'	'61.58% ± 5.31%'	'78.17% ± 4.22%'	'78% ± 4.65%'
'RLR'	'5.33% ± 2.55%'	'22.58% ± 5.08%'	'54.5% ± 4.86%'	'79.33% ± 4.19%'	'80.75% ± 4.17%'
'kNN'	'37.33% ± 4.7%'	'33.67% ± 4.99%'	'58.25% ± 4.72%'	'73% ± 4.57%'	'73% ± 4.17%'

Precision mean precision ± 95% CI

Classifier	5	10	20	40	50
'RF'	'35.83% ± 4.69%'	'51.4% ± 5.95%'	'70.12% ± 4.68%'	'75.68% ± 4.5%'	'73.12% ± 5.05%'
'PLS-DA'	'72.43% ± 3.84%'	'69.62% ± 3.69%'	'63.49% ± 3.61%'	'56.25% ± 3.05%'	'59.52% ± 3.26%'
'SVM'	'7.14% ± 2.39%'	'52.08% ± 5.51%'	'60.33% ± 5.31%'	'75.67% ± 4.33%'	'74.89% ± 4.27%'
'RLR'	'12.5% ± 4.74%'	'41.79% ± 7.4%'	'68.48% ± 5.17%'	'77.49% ± 4.05%'	'77.47% ± 4.04%'
'kNN'	'43.7% ± 5.84%'	'38.96% ± 5.43%'	'63.18% ± 5.19%'	'65.6% ± 4.41%'	'66.58% ± 4.36%'

Specificity mean specificity ± 95% CI

Classifier	5	10	20	40	50
'RF'	'72.41% ± 2.87%'	'79.79% ± 3.16%'	'88.4% ± 2.06%'	'91.36% ± 1.76%'	'90.47% ± 2%'
'PLS-DA'	'82.08% ± 3.25%'	'80.87% ± 2.78%'	'74.63% ± 3.27%'	'67.28% ± 3.35%'	'71.24% ± 3.13%'
'SVM'	'98.23% ± 1.44%'	'82.06% ± 2.81%'	'81.82% ± 2.92%'	'88.93% ± 2.22%'	'88.64% ± 2.1%'
'RLR'	'89.97% ± 2.38%'	'87.93% ± 2.55%'	'87.74% ± 2.47%'	'89.87% ± 2.1%'	'90.28% ± 1.86%'

'kNN'	'79.06% ± 3.13%'	'79.99% ± 2.55%'	'85.26% ± 2.24%'	'84.21% ± 2.32%'	'84.46% ± 2.19%'

NPV mean NPV ± 95% CI

Classifier	5	10	20	40	50
'RF'	'77.89% ± 2.14%'	'81.5% ± 2.37%'	'86.79% ± 1.68%'	'86.75% ± 1.7%'	'86.39% ± 1.75%'
'PLS-DA'	'98.54% ± 0.69%'	'99.41% ± 0.46%'	'99.41% ± 0.46%'	'99.9% ± 0.2%'	'100% ± 0%'
'SVM'	'72.77% ± 0.81%'	'81.56% ± 1.79%'	'85.89% ± 1.85%'	'91.95% ± 1.55%'	'92.08% ± 1.65%'
'RLR'	'71.88% ± 1.17%'	'75.79% ± 1.52%'	'84.33% ± 1.6%'	'92.49% ± 1.51%'	'93.24% ± 1.45%'
'kNN'	'77.33% ± 1.71%'	'76.79% ± 1.66%'	'85.07% ± 1.62%'	'89.76% ± 1.78%'	'89.75% ± 1.53%'

F1 mean F1 ± 95% CI

Classifier	5	10	20	40	50
'RF'	'46.3% ± 3.07%'	'55.97% ± 3.91%'	'63.87% ± 3.54%'	'64.85% ± 3.84%'	'65.25% ± 3.72%'
'PLS-DA'	'80.13% ± 2.25%'	'79.75% ± 2.38%'	'75.28% ± 2.38%'	'70.68% ± 2.31%'	'73.36% ± 2.41%'
'SVM'	'26.79% ± 0.49%'	'53.62% ± 3.36%'	'60.38% ± 3.76%'	'75.11% ± 3.76%'	'74.78% ± 3.8%'
'RLR'	'36.31% ± 1.88%'	'46.84% ± 2.99%'	'59.79% ± 3.25%'	'76.56% ± 3.59%'	'77.45% ± 3.53%'
'kNN'	'43.66% ± 3.04%'	'43.63% ± 3.07%'	'60.81% ± 3.59%'	'67.91% ± 3.67%'	'68.39% ± 3.31%'

Genetic Algorithm: PopSize = 50, Max NumGen= 35

Accuracy accuracy

Classifier	5	10	20	40	50
'RF'	'92.31%'	'92.31%'	'84.62%'	'92.31%'	'76.92%'
'PLS-DA'	'92.31%'	'92.31%'	'84.62%'	'100%'	'76.92%'
'SVM'	'92.31%'	'84.62%'	'100%'	'84.62%'	'92.31%'
'RLR'	'92.31%'	'92.31%'	'100%'	'84.62%'	'92.31%'
'kNN'	'76.92%'	'92.31%'	'92.31%'	'84.62%'	'92.31%'

Recall recall

Classifier	5	10	20	40	50
'RF'	'75%'	'75%'	'50%'	'75%'	'50%'
'PLS-DA'	'100%'	'100%'	'100%'	'100%'	'100%'
'SVM'	'75%'	'75%'	'100%'	'75%'	'100%'
'RLR'	'75%'	'75%'	'100%'	'100%'	'75%'
'kNN'	'75%'	'100%'	'100%'	'75%'	'100%'

Precision precision

Classifier	5	10	20	40	50
'RF'	'100%'	'100%'	'100%'	'100%'	'66.67%'
'PLS-DA'	'80%'	'80%'	'66.67%'	'100%'	'57.14%'
'SVM'	'100%'	'75%'	'100%'	'75%'	'80%'
'RLR'	'100%'	'100%'	'100%'	'66.67%'	'100%'
'kNN'	'60%'	'80%'	'80%'	'75%'	'80%'

Specificity specificity

Classifier	5	10	20	40	50

'RF'	'100%'	'100%'	'100%'	'100%'	'88.89%'
'PLS-DA'	'88.89%'	'88.89%'	'77.78%'	'100%'	'66.67%'
'SVM'	'100%'	'88.89%'	'100%'	'88.89%'	'88.89%'
'RLR'	'100%'	'100%'	'100%'	'77.78%'	'100%'
'kNN'	'77.78%'	'88.89%'	'88.89%'	'88.89%'	'88.89%'

NPV	NPV				
**Classifier**	**5**	**10**	**20**	**40**	**50**
'RF'	'90%'	'90%'	'81.82%'	'90%'	'80%'
'PLS-DA'	'100%'	'100%'	'100%'	'100%'	'100%'
'SVM'	'90%'	'88.89%'	'100%'	'88.89%'	'100%'
'RLR'	'90%'	'90%'	'100%'	'100%'	'90%'
'kNN'	'87.5%'	'100%'	'100%'	'88.89%'	'100%'

F1	F1				
**Classifier**	**5**	**10**	**20**	**40**	**50**
'RF'	'85.71%'	'85.71%'	'66.67%'	'85.71%'	'57.14%'
'PLS-DA'	'88.89%'	'88.89%'	'80%'	'100%'	'72.73%'
'SVM'	'85.71%'	'75%'	'100%'	'75%'	'88.89%'
'RLR'	'85.71%'	'85.71%'	'100%'	'80%'	'85.71%'
'kNN'	'66.67%'	'88.89%'	'88.89%'	'75%'	'88.89%'

Number of Generations					
**Classifier**	**5**	**10**	**20**	**40**	**50**
'RF'	35	35	35	35	35
'PLS-DA'	35	35	35	35	35
'SVM'	35	35	35	35	35
'RLR'	35	35	35	35	35
'kNN'	35	35	35	35	35

This means that in all cases the algorithm stopped after reaching the max number of generations

Genetic Algorithm: PopSize = 200, Max Num Gen = 150

Accuracy	accuracy				
**Classifier**	**5**	**10**	**20**	**40**	**50**
'RF'	'69.23%'	'76.92%'	'92.31%'	'92.31%'	'84.62%'
'PLS-DA'	'100%'	'92.31%'	'92.31%'	'84.62%'	'92.31%'
'SVM'	'84.62%'	'100%'	'84.62%'	'92.31%'	'100%'
'RLR'	'76.92%'	'84.62%'	'100%'	'92.31%'	'92.31%'
'kNN'	'76.92%'	'100%'	'92.31%'	'100%'	'92.31%'

Recall	recall				
**Classifier**	**5**	**10**	**20**	**40**	**50**
'RF'	'75%'	'25%'	'75%'	'75%'	'50%'
'PLS-DA'	'100%'	'100%'	'100%'	'100%'	'100%'
'SVM'	'75%'	'100%'	'75%'	'75%'	'100%'

'RLR'	'50%'	'100%'	'100%'	'75%'	'75%'
'kNN'	'75%'	'100%'	'100%'	'100%'	'100%'

Precision	precision				
**Classifier**	**5**	**10**	**20**	**40**	**50**
'RF'	'50%'	'100%'	'100%'	'100%'	'100%'
'PLS-DA'	'100%'	'80%'	'80%'	'66.67%'	'80%'
'SVM'	'75%'	'100%'	'75%'	'100%'	'100%'
'RLR'	'66.67%'	'66.67%'	'100%'	'100%'	'100%'
'kNN'	'60%'	'100%'	'80%'	'100%'	'80%'

Specificity	specificity				
**Classifier**	**5**	**10**	**20**	**40**	**50**
'RF'	'66.67%'	'100%'	'100%'	'100%'	'100%'
'PLS-DA'	'100%'	'88.89%'	'88.89%'	'77.78%'	'88.89%'
'SVM'	'88.89%'	'100%'	'88.89%'	'100%'	'100%'
'RLR'	'88.89%'	'77.78%'	'100%'	'100%'	'100%'
'kNN'	'77.78%'	'100%'	'88.89%'	'100%'	'88.89%'

NPV	NPV				
**Classifier**	**5**	**10**	**20**	**40**	**50**
'RF'	'85.71%'	'75%'	'90%'	'90%'	'81.82%'
'PLS-DA'	'100%'	'100%'	'100%'	'100%'	'100%'
'SVM'	'88.89%'	'100%'	'88.89%'	'90%'	'100%'
'RLR'	'80%'	'100%'	'100%'	'90%'	'90%'
'kNN'	'87.5%'	'100%'	'100%'	'100%'	'100%'

F1	F1				
**Classifier**	**5**	**10**	**20**	**40**	**50**
'RF'	'60%'	'40%'	'85.71%'	'85.71%'	'66.67%'
'PLS-DA'	'100%'	'88.89%'	'88.89%'	'80%'	'88.89%'
'SVM'	'75%'	'100%'	'75%'	'85.71%'	'100%'
'RLR'	'57.14%'	'80%'	'100%'	'85.71%'	'85.71%'
'kNN'	'66.67%'	'100%'	'88.89%'	'100%'	'88.89%'

Number of Generations					
**Classifier**	**5**	**10**	**20**	**40**	**50**
'RF'	60	63	72	57	58
'PLS-DA'	63	65	64	65	66
'SVM'	58	56	51	53	53
'RLR'	150	150	150	128	150
'kNN'	51	51	51	51	51

Rsp100_FeaturesSets_GLI_85

mRMR

Accuracy: mean accuracy ± 95% CI

Classifier	20%	30%	40%	50%	60%	70%	80%
'RF'	'89.08% ± 1.81%'	'89% ± 1.65%'	'88.54% ± 1.86%'	'88.31% ± 1.77%'	'88.15% ± 1.75%'	'88.62% ± 1.75%'	'88.77% ± 1.58%'
'PLS-DA'	'86.23% ± 1.96%'	'85.54% ± 1.85%'	'85.46% ± 2.13%'	'86% ± 1.73%'	'86.46% ± 1.68%'	'86.85% ± 1.76%'	'85.46% ± 1.87%'
'SVM'	'91.15% ± 1.61%'	'91.38% ± 1.59%'	'90.77% ± 1.63%'	'91% ± 1.6%'	'91.23% ± 1.52%'	'91.31% ± 1.6%'	'91.31% ± 1.63%'
'RLR'	'86.15% ± 1.67%'	'85.77% ± 1.64%'	'85.23% ± 1.7%'	'85.38% ± 1.57%'	'85% ± 1.75%'	'84.85% ± 1.72%'	'85.15% ± 1.65%'
'kNN'	'90.77% ± 1.48%'	'89% ± 1.62%'	'88.77% ± 1.58%'	'87.31% ± 1.97%'	'87.77% ± 1.67%'	'88.08% ± 1.84%'	'86.54% ± 2.06%'

Recall: mean recall ± 95% CI

Classifier	20%	30%	40%	50%	60%	70%	80%
'RF'	'70.33% ± 5.17%'	'68.83% ± 5.07%'	'66.08% ± 5.47%'	'67.58% ± 5.23%'	'66.08% ± 5.26%'	'68% ± 5.36%'	'66.67% ± 5.16%'
'PLS-DA'	'97.75% ± 1.72%'	'98.5% ± 1.17%'	'98.5% ± 1.17%'	'98% ± 1.34%'	'98% ± 1.34%'	'98% ± 1.34%'	'98.75% ± 1.07%'
'SVM'	'78.17% ± 4.41%'	'78% ± 4.31%'	'76.25% ± 4.78%'	'77.17% ± 4.55%'	'77.67% ± 4.32%'	'76.83% ± 4.47%'	'76.33% ± 4.64%'
'RLR'	'69.17% ± 4.87%'	'67.25% ± 4.93%'	'65.58% ± 5.22%'	'66% ± 4.99%'	'65.67% ± 4.93%'	'65.25% ± 5%'	'64.92% ± 4.84%'
'kNN'	'83.75% ± 3.81%'	'79.92% ± 3.79%'	'79.92% ± 4.21%'	'77.33% ± 4.49%'	'77.5% ± 4.2%'	'77.17% ± 4.57%'	'77.17% ± 4.54%'

Precision: mean precision ± 95% CI

Classifier	20%	30%	40%	50%	60%	70%	80%
'RF'	'89.66% ± 3.82%'	'89.05% ± 4.24%'	'88.72% ± 4.75%'	'89.35% ± 3.68%'	'88.3% ± 4.24%'	'88.94% ± 4.16%'	'90.95% ± 3.39%'
'PLS-DA'	'71.42% ± 3.69%'	'69.51% ± 3.43%'	'70.08% ± 3.65%'	'70.33% ± 3.39%'	'70.89% ± 3.34%'	'71.88% ± 3.56%'	'69.46% ± 3.55%'
'SVM'	'89.05% ± 3.38%'	'89.53% ± 3.43%'	'88.32% ± 3.76%'	'88.78% ± 3.78%'	'88.87% ± 3.7%'	'89.8% ± 3.79%'	'90.27% ± 3.75%'
'RLR'	'80.49% ± 4.1%'	'79.95% ± 4.13%'	'79.36% ± 4.65%'	'79.01% ± 4.34%'	'78.47% ± 4.57%'	'78.85% ± 4.5%'	'80.15% ± 4.15%'
'kNN'	'85.42% ± 3.23%'	'82.97% ± 3.52%'	'82.08% ± 3.37%'	'80.45% ± 4.06%'	'81.51% ± 3.53%'	'82.27% ± 3.79%'	'77.01% ± 3.99%'

Specificity: mean specificity ± 95% CI

Classifier	20%	30%	40%	50%	60%	70%	80%
'RF'	'96.3% ± 1.27%'	'96.68% ± 1.15%'	'97.03% ± 1.07%'	'96.31% ± 1.27%'	'96.66% ± 1.14%'	'96.62% ± 1.19%'	'97.2% ± 1%'
'PLS-DA'	'82.21% ± 2.78%'	'80.83% ± 2.64%'	'80.74% ± 3.03%'	'81.69% ± 2.53%'	'82.34% ± 2.45%'	'82.92% ± 2.53%'	'80.61% ± 2.65%'
'SVM'	'96.09% ± 1.1%'	'96.43% ± 1.07%'	'96.33% ± 0.99%'	'96.31% ± 1.08%'	'96.42% ± 0.98%'	'96.87% ± 1.03%'	'97.08% ± 0.97%'
'RLR'	'92.73% ± 1.58%'	'92.92% ± 1.48%'	'92.82% ± 1.55%'	'92.82% ± 1.44%'	'92.4% ± 1.61%'	'92.38% ± 1.59%'	'92.94% ± 1.55%'

'kNN'	'93.63% ± 1.53%'	'92.66% ± 1.64%'	'92.46% ± 1.58%'	'91.31% ± 1.97%'	'91.9% ± 1.82%'	'92.42% ± 1.72%'	'90.41% ± 1.83%'

NPV	mean NPV ± 95% CI						
**Classifier**	**20%**	**30%**	**40%**	**50%**	**60%**	**70%**	**80%**
'RF'	'90.13% ± 1.7%'	'89.87% ± 1.62%'	'89.02% ± 1.73%'	'89.27% ± 1.68%'	'88.93% ± 1.68%'	'89.45% ± 1.71%'	'89.21% ± 1.59%'
'PLS-DA'	'99.14% ± 0.65%'	'99.4% ± 0.47%'	'99.4% ± 0.47%'	'99.2% ± 0.53%'	'99.2% ± 0.53%'	'99.2% ± 0.53%'	'99.5% ± 0.43%'
'SVM'	'92.56% ± 1.51%'	'92.56% ± 1.46%'	'92.05% ± 1.59%'	'92.28% ± 1.54%'	'92.4% ± 1.45%'	'92.17% ± 1.5%'	'92.04% ± 1.54%'
'RLR'	'89.58% ± 1.64%'	'88.95% ± 1.63%'	'88.51% ± 1.69%'	'88.67% ± 1.62%'	'88.43% ± 1.62%'	'88.26% ± 1.64%'	'88.19% ± 1.58%'
'kNN'	'94.17% ± 1.34%'	'92.66% ± 1.4%'	'92.7% ± 1.51%'	'91.87% ± 1.66%'	'91.91% ± 1.51%'	'91.91% ± 1.69%'	'91.48% ± 1.76%'

F1	mean F1 ± 95% CI						
**Classifier**	**20%**	**30%**	**40%**	**50%**	**60%**	**70%**	**80%**
RF'	'77.93% ± 3.54%'	'77.06% ± 3.44%'	'76.79% ± 3.62%'	'76.27% ± 3.59%'	'76.09% ± 3.49%'	'78.3% ± 3.15%'	'76.33% ± 3.52%'
'PLS-DA'	'80.54% ± 2.39%'	'79.93% ± 2.19%'	'80.15% ± 2.38%'	'80.25% ± 2.08%'	'80.68% ± 2.04%'	'81.22% ± 2.19%'	'79.96% ± 2.28%'
'SVM'	'82.29% ± 3.15%'	'82.6% ± 3.14%'	'81.45% ± 3.27%'	'82.44% ± 3.03%'	'83.04% ± 2.8%'	'82.88% ± 3.03%'	'82.56% ± 3.22%'
'RLR'	'72.5% ± 3.28%'	'71.19% ± 3.42%'	'71.01% ± 3.31%'	'70.72% ± 3.25%'	'69.81% ± 3.55%'	'69.35% ± 3.51%'	'69.04% ± 3.64%'
'kNN'	'82.56% ± 2.83%'	'79.43% ± 3%'	'79.41% ± 2.87%'	'76.96% ± 3.24%'	'76.86% ± 3.08%'	'77.78% ± 3.12%'	'75.98% ± 3.37%'

Relief-F

Accuracy	mean accuracy ± 95% CI						
**Classifier**	**20%**	**30%**	**40%**	**50%**	**60%**	**70%**	**80%**
'RF'	'90% ± 1.6%'	'89.62% ± 1.55%'	'89.46% ± 1.63%'	'89.54% ± 1.6%'	'89% ± 1.74%'	'89.38% ± 1.64%'	'88.54% ± 1.79%'
'PLS-DA'	'88.62% ± 1.84%'	'88.46% ± 1.58%'	'89.23% ± 1.56%'	'88.69% ± 1.65%'	'87.92% ± 1.59%'	'87.38% ± 1.67%'	'86.46% ± 1.72%'
'SVM'	'91.92% ± 1.54%'	'91.77% ± 1.44%'	'91.85% ± 1.47%'	'91.85% ± 1.45%'	'91.77% ± 1.47%'	'91.77% ± 1.53%'	'91.23% ± 1.65%'
'RLR'	'85% ± 1.65%'	'84.62% ± 1.77%'	'85.31% ± 1.65%'	'84.92% ± 1.74%'	'84.92% ± 1.79%'	'84.92% ± 1.75%'	'85% ± 1.8%'
'kNN'	'91.54% ± 1.4%'	'91% ± 1.44%'	'90.54% ± 1.53%'	'90.31% ± 1.6%'	'90.08% ± 1.59%'	'89.69% ± 1.72%'	'88.85% ± 1.76%'

Recall	mean recall ± 95% CI						
**Classifier**	**20%**	**30%**	**40%**	**50%**	**60%**	**70%**	**80%**
'RF'	'71.08% ± 5.21%'	'69.33% ± 5.23%'	'67.67% ± 5.37%'	'67.08% ± 5.38%'	'68.17% ± 5.46%'	'68.67% ± 5.2%'	'64.92% ± 5.44%'
'PLS-DA'	'98.25% ± 1.26%'	'97.5% ± 1.48%'	'98% ± 1.34%'	'98.25% ± 1.26%'	'97.5% ± 1.48%'	'97.5% ± 1.48%'	'97.25% ± 1.54%'
'SVM'	'79.25% ± 4.32%'	'77.75% ± 4.48%'	'77.92% ± 4.4%'	'77.67% ± 4.38%'	'77.92% ± 4.35%'	'77.67% ± 4.38%'	'75.75% ± 4.79%'
'RLR'	'63.92% ± 4.63%'	'62.75% ± 4.9%'	'64.83% ± 4.73%'	'64.17% ± 5.01%'	'64% ± 5.02%'	'64% ± 4.84%'	'64.5% ± 4.88%'

'kNN'	'78.33% ± 4.17%'	'78.92% ± 4.04%'	'79.17% ± 4.24%'	'79.17% ± 4.33%'	'79.33% ± 4.4%'	'79.67% ± 4.19%'	'76.83% ± 4.53%'

Precision	mean precision ± 95% CI						
**Classifier**	**20%**	**30%**	**40%**	**50%**	**60%**	**70%**	**80%**
'RF'	'91.65% ± 3.28%'	'92.18% ± 2.83%'	'93.73% ± 2.58%'	'93.59% ± 3.04%'	'89.91% ± 4.09%'	'90.7% ± 3.71%'	'90.12% ± 4.41%'
'PLS-DA'	'75.3% ± 3.57%'	'74.68% ± 3.29%'	'75.8% ± 3.34%'	'74.73% ± 3.46%'	'73.57% ± 3.31%'	'73.12% ± 3.51%'	'71.67% ± 3.49%'
'SVM'	'90.83% ± 3.24%'	'91.17% ± 3.21%'	'91.33% ± 3.21%'	'91.62% ± 3.19%'	'91.03% ± 3.23%'	'90.62% ± 3.66%'	'90.43% ± 3.69%'
'RLR'	'80.05% ± 4.49%'	'79.05% ± 4.68%'	'80.77% ± 4.46%'	'79.57% ± 4.58%'	'79.65% ± 4.66%'	'79.33% ± 4.71%'	'79.5% ± 4.66%'
'kNN'	'91.23% ± 2.74%'	'89.33% ± 3.07%'	'87.43% ± 3.13%'	'86.05% ± 3.19%'	'85.12% ± 3.26%'	'84.03% ± 3.54%'	'83.01% ± 3.54%'

Specificit y	mean specificity ± 95% CI						
**Classifier**	**20%**	**30%**	**40%**	**50%**	**60%**	**70%**	**80%**
'RF'	'97.3% ± 0.98%'	'97.3% ± 0.99%'	'97.83% ± 0.93%'	'98.12% ± 0.79%'	'96.96% ± 1.25%'	'97.26% ± 0.91%'	'97.59% ± 0.92%'
'PLS-DA'	'85.19% ± 2.63%'	'85.27% ± 2.37%'	'86.17% ± 2.25%'	'85.38% ± 2.34%'	'84.56% ± 2.4%'	'83.79% ± 2.49%'	'82.6% ± 2.61%'
'SVM'	'96.76% ± 1.04%'	'97.08% ± 0.92%'	'97.18% ± 0.92%'	'97.29% ± 0.9%'	'97.07% ± 0.93%'	'97.17% ± 0.92%'	'97.17% ± 0.92%'
'RLR'	'93.14% ± 1.54%'	'93.04% ± 1.56%'	'93.23% ± 1.57%'	'92.91% ± 1.59%'	'93.03% ± 1.59%'	'92.92% ± 1.61%'	'92.93% ± 1.61%'
'kNN'	'96.63% ± 1.05%'	'95.72% ± 1.27%'	'94.96% ± 1.29%'	'94.62% ± 1.27%'	'94.21% ± 1.32%'	'93.69% ± 1.44%'	'93.48% ± 1.43%'

NPV	mean NPV ± 95% CI						
**Classifier**	**20%**	**30%**	**40%**	**50%**	**60%**	**70%**	**80%**
'RF'	'90.57% ± 1.67%'	'90.1% ± 1.62%'	'89.64% ± 1.68%'	'89.43% ± 1.65%'	'89.75% ± 1.7%'	'89.82% ± 1.66%'	'88.71% ± 1.75%'
'PLS-DA'	'99.3% ± 0.5%'	'99% ± 0.59%'	'99.2% ± 0.53%'	'99.3% ± 0.5%'	'99% ± 0.59%'	'99% ± 0.59%'	'98.9% ± 0.62%'
'SVM'	'92.98% ± 1.46%'	'92.6% ± 1.45%'	'92.6% ± 1.46%'	'92.51% ± 1.45%'	'92.56% ± 1.44%'	'92.47% ± 1.47%'	'91.9% ± 1.58%'
'RLR'	'87.79% ± 1.52%'	'87.47% ± 1.61%'	'88.1% ± 1.54%'	'87.95% ± 1.63%'	'87.85% ± 1.65%'	'87.88% ± 1.56%'	'87.98% ± 1.61%'
'kNN'	'92.6% ± 1.44%'	'92.7% ± 1.4%'	'92.79% ± 1.48%'	'92.72% ± 1.51%'	'92.82% ± 1.5%'	'92.72% ± 1.54%'	'91.82% ± 1.54%'

F1	mean F1 ± 95% CI						
**Classifier**	**20%**	**30%**	**40%**	**50%**	**60%**	**70%**	**80%**
'RF'	'79.06% ± 3.47%'	'76.92% ± 3.76%'	'77.4% ± 3.66%'	'78.27% ± 3.4%'	'78.58% ± 3.3%'	'76.78% ± 3.71%'	'76.2% ± 3.6%'
'PLS-DA'	'83.66% ± 2.25%'	'83.02% ± 1.94%'	'84.02% ± 2.03%'	'83.37% ± 2.15%'	'82.27% ± 1.93%'	'81.83% ± 2.07%'	'80.71% ± 2.01%'
'SVM'	'83.71% ± 3.03%'	'82.76% ± 3.16%'	'83.86% ± 2.85%'	'83.82% ± 2.82%'	'83.8% ± 2.83%'	'83.86% ± 2.87%'	'82.12% ± 3.37%'
'RLR'	'69.32% ± 3.37%'	'68.18% ± 3.69%'	'70.04% ± 3.38%'	'68.97% ± 3.67%'	'68.98% ± 3.72%'	'69.17% ± 3.66%'	'69.39% ± 3.69%'
'kNN'	'82.31% ± 2.97%'	'81.71% ± 2.99%'	'80.91% ± 3.15%'	'80.55% ± 3.31%'	'80.83% ± 3.19%'	'80.12% ± 3.35%'	'79.1% ± 3.32%'

Chi-Squared

Accuracy	mean accuracy ± 95% CI						
**Classifier**	**20%**	**30%**	**40%**	**50%**	**60%**	**70%**	**80%**
'RF'	'90.23% ± 1.51%'	'89.69% ± 1.69%'	'88.31% ± 1.61%'	'89% ± 1.74%'	'88.85% ± 1.77%'	'88.92% ± 1.66%'	'87.69% ± 1.87%'
'PLS-DA'	'87.31% ± 1.94%'	'87.62% ± 1.73%'	'86.62% ± 1.72%'	'87.46% ± 1.7%'	'86.38% ± 1.99%'	'86.23% ± 1.87%'	'86.23% ± 1.82%'
'SVM'	'90.92% ± 1.62%'	'91.23% ± 1.67%'	'91.46% ± 1.59%'	'91.77% ± 1.55%'	'91.46% ± 1.62%'	'91.62% ± 1.55%'	'91.69% ± 1.5%'
'RLR'	'85.69% ± 1.59%'	'85.31% ± 1.74%'	'84.62% ± 1.75%'	'85.15% ± 1.78%'	'85.46% ± 1.77%'	'84.77% ± 1.78%'	'84.85% ± 1.82%'
'kNN'	'89.46% ± 1.6%'	'88.62% ± 1.79%'	'87.62% ± 1.88%'	'88.23% ± 1.69%'	'87.69% ± 1.78%'	'87.92% ± 1.96%'	'87.31% ± 1.85%'

Recall	mean recall ± 95% CI						
**Classifier**	**20%**	**30%**	**40%**	**50%**	**60%**	**70%**	**80%**
'RF'	'73.33% ± 4.76%'	'72.42% ± 5.06%'	'66.75% ± 5%'	'67.83% ± 5.25%'	'67.92% ± 5.17%'	'68.08% ± 5.3%'	'65.08% ± 5.23%'
'PLS-DA'	'98.5% ± 1.17%'	'98.25% ± 1.26%'	'98.75% ± 1.07%'	'98.25% ± 1.26%'	'99.25% ± 0.84%'	'98.5% ± 1.17%'	'98.25% ± 1.26%'
'SVM'	'77.75% ± 4.42%'	'77.5% ± 4.57%'	'77.25% ± 4.56%'	'78.25% ± 4.42%'	'77.08% ± 4.69%'	'76.75% ± 4.58%'	'77.17% ± 4.45%'
'RLR'	'67% ± 4.8%'	'64.83% ± 5.02%'	'63.42% ± 4.79%'	'64.08% ± 4.76%'	'65.33% ± 4.86%'	'62.92% ± 4.93%'	'64% ± 4.98%'
'kNN'	'76% ± 4.75%'	'78.25% ± 4.6%'	'76.25% ± 4.29%'	'76.58% ± 4.51%'	'78.08% ± 4.03%'	'79.17% ± 4.38%'	'77.67% ± 4.4%'

Precision	mean precision ± 95% CI						
**Classifier**	**20%**	**30%**	**40%**	**50%**	**60%**	**70%**	**80%**
'RF'	'91.01% ± 2.87%'	'88.1% ± 4.11%'	'89.58% ± 3.55%'	'89.95% ± 3.91%'	'91.03% ± 3.46%'	'89.34% ± 3.9%'	'88.03% ± 4.21%'
'PLS-DA'	'72.6% ± 3.5%'	'72.78% ± 3.36%'	'70.68% ± 3.39%'	'72.45% ± 3.37%'	'70.62% ± 3.49%'	'70.67% ± 3.54%'	'70.71% ± 3.46%'
'SVM'	'88.75% ± 3.48%'	'89.45% ± 3.45%'	'90.62% ± 3.31%'	'90.45% ± 3.38%'	'89.9% ± 3.72%'	'91.31% ± 3.26%'	'91.48% ± 3.22%'
'RLR'	'81.5% ± 3.92%'	'80.17% ± 4.56%'	'79.3% ± 4.59%'	'79.8% ± 4.64%'	'80.07% ± 4.59%'	'78.73% ± 4.66%'	'78.82% ± 4.83%'
'kNN'	'86.25% ± 3.35%'	'82.76% ± 3.64%'	'80.28% ± 3.79%'	'82.46% ± 3.62%'	'79.95% ± 3.66%'	'79.68% ± 3.88%'	'78.39% ± 3.69%'

Specificity	mean specificity ± 95% CI						
**Classifier**	**20%**	**30%**	**40%**	**50%**	**60%**	**70%**	**80%**
'RF'	'96.66% ± 1.13%'	'96.23% ± 1.12%'	'96.54% ± 1.19%'	'97.08% ± 1.04%'	'96.84% ± 1.29%'	'96.76% ± 1.08%'	'96.22% ± 1.31%'
'PLS-DA'	'83.36% ± 2.73%'	'83.88% ± 2.45%'	'82.28% ± 2.44%'	'83.67% ± 2.42%'	'81.69% ± 2.8%'	'81.84% ± 2.67%'	'81.89% ± 2.66%'
'SVM'	'96% ± 1.13%'	'96.53% ± 1.02%'	'96.86% ± 1%'	'96.96% ± 0.94%'	'96.97% ± 0.94%'	'97.29% ± 0.9%'	'97.29% ± 0.9%'
'RLR'	'92.93% ± 1.61%'	'93.22% ± 1.53%'	'92.8% ± 1.59%'	'93.21% ± 1.56%'	'93.23% ± 1.55%'	'93.12% ± 1.49%'	'92.83% ± 1.63%'
'kNN'	'94.53% ± 1.42%'	'92.76% ± 1.7%'	'92.11% ± 1.68%'	'92.86% ± 1.59%'	'91.58% ± 1.73%'	'91.47% ± 1.76%'	'91.24% ± 1.62%'

NPV	mean NPV ± 95% CI

Classifier	20%	30%	40%	50%	60%	70%	80%
'RF'	'91.16% ± 1.48%'	'90.92% ± 1.61%'	'89.14% ± 1.6%'	'89.56% ± 1.67%'	'89.43% ± 1.64%'	'89.7% ± 1.64%'	'88.64% ± 1.7%'
'PLS-DA'	'99.4% ± 0.47%'	'99.3% ± 0.5%'	'99.5% ± 0.43%'	'99.3% ± 0.5%'	'99.7% ± 0.34%'	'99.4% ± 0.47%'	'99.3% ± 0.5%'
'SVM'	'92.37% ± 1.53%'	'92.35% ± 1.57%'	'92.39% ± 1.53%'	'92.65% ± 1.5%'	'92.33% ± 1.55%'	'92.26% ± 1.51%'	'92.32% ± 1.47%'
'RLR'	'88.87% ± 1.57%'	'88.17% ± 1.65%'	'87.6% ± 1.6%'	'87.84% ± 1.57%'	'88.25% ± 1.59%'	'87.54% ± 1.61%'	'87.89% ± 1.62%'
'kNN'	'91.94% ± 1.55%'	'92.24% ± 1.58%'	'91.41% ± 1.63%'	'91.74% ± 1.59%'	'91.96% ± 1.54%'	'92.38% ± 1.68%'	'91.74% ± 1.63%'

F1	mean F1 ± 95% CI						
**Classifier**	**20%**	**30%**	**40%**	**50%**	**60%**	**70%**	**80%**
'RF'	'80.4% ± 3.14%'	'79.94% ± 3.17%'	'75.3% ± 3.49%'	'77.95% ± 3.46%'	'76.63% ± 3.68%'	'76.3% ± 3.78%'	'74.45% ± 3.76%'
'PLS-DA'	'82.01% ± 2.27%'	'82.11% ± 2.12%'	'80.94% ± 2.15%'	'81.88% ± 2.11%'	'81.1% ± 2.31%'	'80.67% ± 2.26%'	'80.62% ± 2.14%'
'SVM'	'81.9% ± 3.15%'	'82.11% ± 3.41%'	'82.28% ± 3.28%'	'83.16% ± 3.19%'	'82.87% ± 3.21%'	'83.02% ± 3.13%'	'83.41% ± 2.97%'
'RLR'	'70.45% ± 3.46%'	'69.79% ± 3.67%'	'68.57% ± 3.54%'	'69.68% ± 3.6%'	'70.41% ± 3.64%'	'68.41% ± 3.78%'	'69% ± 3.8%'
'kNN'	'78.65% ± 3.37%'	'79.22% ± 3.25%'	'76.42% ± 3.45%'	'77.66% ± 3.26%'	'77.23% ± 3.19%'	'78.42% ± 3.21%'	'76.91% ± 3.36%'

Variance

Accuracy	mean accuracy ± 95% CI						
**Classifier**	**20%**	**30%**	**40%**	**50%**	**60%**	**70%**	**80%**
'RF'	'87.69% ± 1.79%'	'87.69% ± 1.82%'	'87.77% ± 1.93%'	'88.08% ± 1.77%'	'89.15% ± 1.87%'	'88.46% ± 1.69%'	'88.54% ± 1.76%'
'PLS-DA'	'85.62% ± 1.72%'	'85.54% ± 1.87%'	'84.54% ± 1.85%'	'85.15% ± 1.85%'	'85.92% ± 1.82%'	'84.77% ± 2.06%'	'84.92% ± 1.89%'
'SVM'	'90.31% ± 1.52%'	'91.31% ± 1.45%'	'90.77% ± 1.52%'	'90.46% ± 1.58%'	'91.08% ± 1.6%'	'91.77% ± 1.5%'	'92% ± 1.48%'
'RLR'	'89.08% ± 1.47%'	'88.85% ± 1.46%'	'87.77% ± 1.62%'	'88% ± 1.64%'	'85.77% ± 1.59%'	'85.85% ± 1.53%'	'85.23% ± 1.61%'
'kNN'	'91.08% ± 1.56%'	'90.08% ± 1.47%'	'88.54% ± 1.75%'	'87.92% ± 1.61%'	'87% ± 1.91%'	'87.08% ± 1.93%'	'86.69% ± 1.75%'

Recall	mean recall ± 95% CI						
**Classifier**	**20%**	**30%**	**40%**	**50%**	**60%**	**70%**	**80%**
'RF'	'68.58% ± 4.98%'	'67% ± 4.85%'	'67.5% ± 5.11%'	'67% ± 5.07%'	'69.5% ± 5.02%'	'66.17% ± 5.2%'	'67.58% ± 5.14%'
'PLS-DA'	'98.75% ± 1.07%'	'98.25% ± 1.26%'	'96.92% ± 1.92%'	'98.25% ± 1.26%'	'99% ± 0.97%'	'99.25% ± 0.84%'	'97.67% ± 1.47%'
'SVM'	'75.42% ± 4.35%'	'77.58% ± 4.04%'	'76.83% ± 4.34%'	'77.17% ± 4.45%'	'79.08% ± 4.28%'	'78.33% ± 4.49%'	'78% ± 4.57%'
'RLR'	'77.42% ± 4.66%'	'76.92% ± 5.09%'	'72.58% ± 5.09%'	'73.92% ± 4.56%'	'67.5% ± 4.39%'	'67.75% ± 4.52%'	'66.08% ± 4.74%'
'kNN'	'88.17% ± 3.47%'	'85.5% ± 3.92%'	'82.25% ± 4.12%'	'80% ± 3.96%'	'80% ± 4.29%'	'78.08% ± 4.7%'	'75% ± 4.66%'

Precision	mean precision ± 95% CI						
**Classifier**	**20%**	**30%**	**40%**	**50%**	**60%**	**70%**	**80%**

'RF'	'84.75% ± 4.23%'	'87.65% ± 3.88%'	'86.6% ± 4.32%'	'87.74% ± 4.27%'	'89.16% ± 4.3%'	'90.65% ± 3.54%'	'89.18% ± 4.04%'
'PLS-DA'	'69.11% ± 3.4%'	'69.67% ± 3.52%'	'68.99% ± 3.7%'	'68.94% ± 3.39%'	'69.53% ± 3.39%'	'68.19% ± 3.48%'	'69.13% ± 3.57%'
'SVM'	'89.19% ± 3.02%'	'90.33% ± 3%'	'89.19% ± 3.1%'	'87.37% ± 3.61%'	'87.5% ± 3.66%'	'90.94% ± 3.27%'	'91.62% ± 3.19%'
'RLR'	'84.94% ± 3.4%'	'84.86% ± 3.3%'	'84.19% ± 3.59%'	'83.52% ± 3.68%'	'79.77% ± 4.09%'	'79.92% ± 3.67%'	'80.07% ± 3.89%'
'kNN'	'82.35% ± 3.24%'	'81.46% ± 3.21%'	'78.82% ± 3.64%'	'78.63% ± 3.38%'	'76.66% ± 3.8%'	'77.82% ± 3.89%'	'77.48% ± 3.61%'

Specificity	mean specificity ± 95% CI						
**Classifier**	**20%**	**30%**	**40%**	**50%**	**60%**	**70%**	**80%**
'RF'	'94.93% ± 1.35%'	'95.57% ± 1.54%'	'95.47% ± 1.43%'	'96.2% ± 1.21%'	'96.63% ± 1.25%'	'96.96% ± 1.11%'	'96.66% ± 1.2%'
'PLS-DA'	'80.87% ± 2.47%'	'81% ± 2.69%'	'80.19% ± 2.73%'	'80.43% ± 2.66%'	'81.21% ± 2.56%'	'79.46% ± 2.87%'	'80.33% ± 2.78%'
'SVM'	'95.99% ± 1.14%'	'96.51% ± 1.07%'	'95.98% ± 1.19%'	'95.59% ± 1.23%'	'95.56% ± 1.23%'	'96.84% ± 1.05%'	'97.29% ± 0.9%'
'RLR'	'93.78% ± 1.56%'	'93.68% ± 1.47%'	'93.63% ± 1.54%'	'93.47% ± 1.52%'	'92.69% ± 1.48%'	'92.83% ± 1.38%'	'92.72% ± 1.52%'
'kNN'	'92.14% ± 1.49%'	'91.78% ± 1.54%'	'90.86% ± 1.69%'	'90.9% ± 1.58%'	'89.76% ± 1.82%'	'90.52% ± 1.77%'	'91.18% ± 1.56%'

NPV	mean NPV ± 95% CI						
**Classifier**	**20%**	**30%**	**40%**	**50%**	**60%**	**70%**	**80%**
'RF'	'89.5% ± 1.62%'	'89.01% ± 1.61%'	'89.16% ± 1.68%'	'89.03% ± 1.65%'	'89.92% ± 1.68%'	'89.02% ± 1.64%'	'89.29% ± 1.68%'
'PLS-DA'	'99.5% ± 0.43%'	'99.3% ± 0.5%'	'98.85% ± 0.71%'	'99.3% ± 0.5%'	'99.6% ± 0.39%'	'99.7% ± 0.34%'	'99.11% ± 0.56%'
'SVM'	'91.62% ± 1.44%'	'92.34% ± 1.38%'	'92.22% ± 1.44%'	'92.22% ± 1.49%'	'92.92% ± 1.45%'	'92.79% ± 1.47%'	'92.74% ± 1.48%'
'RLR'	'92.28% ± 1.55%'	'92.18% ± 1.61%'	'90.82% ± 1.64%'	'91.04% ± 1.53%'	'88.91% ± 1.48%'	'89.01% ± 1.52%'	'88.44% ± 1.59%'
'kNN'	'95.7% ± 1.27%'	'94.91% ± 1.29%'	'93.64% ± 1.38%'	'92.77% ± 1.4%'	'92.62% ± 1.59%'	'92.18% ± 1.65%'	'91.21% ± 1.61%'

F1	mean F1 ± 95% CI						
**Classifier**	**20%**	**30%**	**40%**	**50%**	**60%**	**70%**	**80%**
'RF'	'75.33% ± 3.6%'	'75.11% ± 3.45%'	'75.09% ± 3.8%'	'76.83% ± 3.18%'	'78.77% ± 3.28%'	'75.84% ± 3.61%'	'76.11% ± 3.68%'
'PLS-DA'	'79.82% ± 2.16%'	'79.83% ± 2.23%'	'78.35% ± 2.26%'	'79.39% ± 2.15%'	'80.26% ± 2.21%'	'79.37% ± 2.35%'	'79.07% ± 2.18%'
'SVM'	'80.17% ± 3.24%'	'81.88% ± 3.17%'	'81.26% ± 3.14%'	'81.63% ± 3.05%'	'82.47% ± 3.17%'	'83.74% ± 2.91%'	'83.83% ± 3.01%'
'RLR'	'78.53% ± 3.1%'	'79.58% ± 2.76%'	'76.17% ± 3.32%'	'75.41% ± 3.5%'	'71.13% ± 3.23%'	'70.38% ± 3.41%'	'69.6% ± 3.48%'
'kNN'	'84.08% ± 2.84%'	'82.34% ± 2.77%'	'79.49% ± 3.23%'	'77.58% ± 3.06%'	'77.16% ± 3.16%'	'77.65% ± 3.1%'	'75.32% ± 3.3%'

Rsp100_Biomarkers_CLL_SUB_111

mRMR

Accuracy — mean accuracy ± 95% CI

Classifier	5	10	20	40	50
'RF'	'69.18% ± 1.97%'	'72% ± 1.97%'	'72.41% ± 2.18%'	'72.24% ± 2.05%'	'74.88% ± 2.06%'
'PLS-DA'	'62.12% ± 2.19%'	'64.71% ± 2.21%'	'68.29% ± 2.04%'	'69.59% ± 2.25%'	'71.82% ± 1.98%'
'SVM'	'68.82% ± 2.07%'	'68.94% ± 2.29%'	'67.88% ± 2.27%'	'70.76% ± 2.2%'	'72.35% ± 1.95%'
'RLR'	'68.94% ± 2.21%'	'68.76% ± 2.16%'	'68.94% ± 2.3%'	'70.76% ± 2.23%'	'71.53% ± 2.12%'
'kNN'	'67.47% ± 2.16%'	'67.76% ± 2.18%'	'67% ± 2%'	'66.88% ± 2.3%'	'66% ± 2%'

Recall — mean recall ± 95% CI

Classifier	5	10	20	40	50
'RF'	'75.55% ± 1.81%'	'79.07% ± 1.49%'	'79.42% ± 1.63%'	'79.28% ± 1.54%'	'81.26% ± 1.56%'
'PLS-DA'	'71.7% ± 1.64%'	'73.7% ± 1.63%'	'76.39% ± 1.51%'	'77.35% ± 1.66%'	'79.05% ± 1.47%'
'SVM'	'75.55% ± 1.8%'	'76.39% ± 1.89%'	'76.18% ± 1.65%'	'78.14% ± 1.66%'	'79.35% ± 1.44%'
'RLR'	'75.74% ± 2.02%'	'75.48% ± 1.94%'	'76.18% ± 1.84%'	'77.2% ± 1.97%'	'77.17% ± 1.98%'
'kNN'	'73.95% ± 2.06%'	'76.07% ± 1.59%'	'75.29% ± 1.51%'	'75.31% ± 1.69%'	'74.75% ± 1.44%'

Precision — mean precision ± 95% CI

Classifier	5	10	20	40	50
'RF'	'76.87% ± 1.71%'	'79.83% ± 1.51%'	'80.11% ± 1.63%'	'80.14% ± 1.57%'	'82.18% ± 1.56%'
'PLS-DA'	'62.73% ± 2.21%'	'66.6% ± 2.24%'	'73.7% ± 1.91%'	'75.98% ± 2.07%'	'76.95% ± 1.87%'
'SVM'	'77% ± 1.92%'	'77.68% ± 1.9%'	'77.13% ± 1.77%'	'79.27% ± 1.72%'	'80.45% ± 1.5%'
'RLR'	'75.47% ± 2.19%'	'76.79% ± 1.78%'	'77.84% ± 1.78%'	'79.41% ± 1.69%'	'79.51% ± 1.7%'
'kNN'	'75.58% ± 2%'	'76.92% ± 1.74%'	'76.38% ± 1.6%'	'76.62% ± 1.94%'	'75.99% ± 1.68%'

Specificity — mean specificity ± 95% CI

Classifier	5	10	20	40	50
'RF'	'81.46% ± 1.21%'	'83.05% ± 1.21%'	'83.3% ± 1.35%'	'83.2% ± 1.26%'	'84.81% ± 1.26%'
'PLS-DA'	'80.2% ± 1.23%'	'80.72% ± 1.27%'	'81.45% ± 1.27%'	'81.97% ± 1.37%'	'83.44% ± 1.2%'
'SVM'	'81.21% ± 1.25%'	'81.18% ± 1.4%'	'80.53% ± 1.4%'	'82.26% ± 1.34%'	'83.23% ± 1.2%'
'RLR'	'81.46% ± 1.31%'	'81.18% ± 1.32%'	'81.18% ± 1.42%'	'82.31% ± 1.35%'	'82.71% ± 1.31%'
'kNN'	'80.37% ± 1.32%'	'80.49% ± 1.34%'	'79.95% ± 1.24%'	'79.84% ± 1.43%'	'79.37% ± 1.25%'

NPV — mean NPV ± 95% CI

Classifier	5	10	20	40	50
'RF'	'82.11% ± 1.21%'	'83.7% ± 1.21%'	'83.82% ± 1.35%'	'83.8% ± 1.26%'	'85.47% ± 1.26%'
'PLS-DA'	'80.16% ± 1.23%'	'80.83% ± 1.27%'	'81.92% ± 1.27%'	'82.63% ± 1.37%'	'83.81% ± 1.2%'
'SVM'	'82.23% ± 1.25%'	'82.01% ± 1.4%'	'81.42% ± 1.4%'	'83.11% ± 1.34%'	'84.06% ± 1.2%'
'RLR'	'82.12% ± 1.31%'	'81.94% ± 1.32%'	'81.92% ± 1.42%'	'83.11% ± 1.35%'	'83.39% ± 1.31%'
'kNN'	'81.37% ± 1.32%'	'81.52% ± 1.34%'	'80.99% ± 1.24%'	'81.2% ± 1.43%'	'80.63% ± 1.25%'

F1 — mean F1 ± 95% CI

Classifier	5	10	20	40	50

'RF'	'75.48% ± 1.67%'	'78.61% ± 1.55%'	'79.1% ± 1.64%'	'78.97% ± 1.55%'	'80.94% ± 1.58%'
'PLS-DA'	'62.31% ± 2.08%'	'66.42% ± 2.15%'	'73.34% ± 1.74%'	'75.45% ± 1.93%'	'76.69% ± 1.77%'
'SVM'	'74.96% ± 1.81%'	'75.95% ± 1.86%'	'75.5% ± 1.71%'	'77.7% ± 1.69%'	'78.9% ± 1.5%'
'RLR'	'74.77% ± 2.02%'	'75.34% ± 1.7%'	'75.95% ± 1.78%'	'77.1% ± 1.88%'	'77.43% ± 1.79%'
'kNN'	'73.69% ± 1.86%'	'75.18% ± 1.68%'	'74.51% ± 1.59%'	'74.25% ± 1.81%'	'73.71% ± 1.56%'

Relief-F

Accuracy mean accuracy ± 95% CI

**Classifier**	**5**	**10**	**20**	**40**	**50**
'RF'	'66.35% ± 2.13%'	'68% ± 1.83%'	'71.18% ± 1.78%'	'74.53% ± 1.83%'	'77% ± 1.55%'
'PLS-DA'	'63.94% ± 2.16%'	'68% ± 2.02%'	'71.29% ± 1.97%'	'74.71% ± 1.92%'	'75.06% ± 1.89%'
'SVM'	'67.12% ± 2.11%'	'70% ± 1.93%'	'72.41% ± 1.89%'	'75.53% ± 1.86%'	'76.41% ± 1.79%'
'RLR'	'67.06% ± 1.97%'	'69.76% ± 1.85%'	'72.59% ± 1.85%'	'76.53% ± 1.82%'	'76.71% ± 1.69%'
'kNN'	'65.35% ± 2.19%'	'65.82% ± 1.91%'	'69.06% ± 2.02%'	'73.35% ± 2.11%'	'73.71% ± 2.02%'

Recall mean recall ± 95% CI

**Classifier**	**5**	**10**	**20**	**40**	**50**
'RF'	'74.15% ± 1.74%'	'75.96% ± 1.44%'	'78.32% ± 1.38%'	'80.88% ± 1.48%'	'82.64% ± 1.32%'
'PLS-DA'	'72.94% ± 1.66%'	'76.28% ± 1.48%'	'78.71% ± 1.46%'	'81.1% ± 1.5%'	'81.52% ± 1.37%'
'SVM'	'74.05% ± 1.99%'	'77.29% ± 1.51%'	'79.4% ± 1.45%'	'81.59% ± 1.51%'	'82.27% ± 1.46%'
'RLR'	'75.03% ± 1.62%'	'76.52% ± 1.55%'	'78.59% ± 1.57%'	'81.86% ± 1.61%'	'81.98% ± 1.51%'
'kNN'	'73.92% ± 1.67%'	'74.33% ± 1.53%'	'77.01% ± 1.5%'	'80.05% ± 1.64%'	'80.23% ± 1.65%'

Precision mean precision ± 95% CI

**Classifier**	**5**	**10**	**20**	**40**	**50**
'RF'	'74.2% ± 1.91%'	'75.9% ± 1.62%'	'78.45% ± 1.52%'	'80.61% ± 1.67%'	'82.68% ± 1.36%'
'PLS-DA'	'66.42% ± 2.45%'	'72.52% ± 2.27%'	'75.58% ± 2.11%'	'78.22% ± 2.03%'	'78.77% ± 1.95%'
'SVM'	'75.31% ± 1.85%'	'78.49% ± 1.65%'	'80.28% ± 1.55%'	'82.49% ± 1.47%'	'83.55% ± 1.41%'
'RLR'	'74.41% ± 1.88%'	'78.02% ± 1.62%'	'80.6% ± 1.54%'	'83.58% ± 1.38%'	'83.7% ± 1.28%'
'kNN'	'74.15% ± 1.92%'	'75.3% ± 1.68%'	'77.34% ± 1.71%'	'81.48% ± 1.69%'	'81.65% ± 1.67%'

Specificity mean specificity ± 95% CI

**Classifier**	**5**	**10**	**20**	**40**	**50**
'RF'	'79.84% ± 1.27%'	'80.77% ± 1.11%'	'82.7% ± 1.09%'	'84.78% ± 1.1%'	'86.24% ± 0.94%'
'PLS-DA'	'79.91% ± 1.25%'	'81.44% ± 1.19%'	'83.3% ± 1.13%'	'85.3% ± 1.12%'	'85.47% ± 1.14%'
'SVM'	'80.33% ± 1.27%'	'81.91% ± 1.18%'	'83.35% ± 1.14%'	'85.19% ± 1.14%'	'85.71% ± 1.11%'
'RLR'	'80.32% ± 1.2%'	'81.82% ± 1.12%'	'83.49% ± 1.12%'	'85.82% ± 1.1%'	'85.91% ± 1.04%'
'kNN'	'79.2% ± 1.33%'	'79.39% ± 1.15%'	'81.35% ± 1.22%'	'83.87% ± 1.29%'	'84.09% ± 1.23%'

NPV mean NPV ± 95% CI

**Classifier**	**5**	**10**	**20**	**40**	**50**
'RF'	'80.32% ± 1.27%'	'81.25% ± 1.11%'	'83.35% ± 1.09%'	'85.34% ± 1.1%'	'86.82% ± 0.94%'
'PLS-DA'	'80.04% ± 1.25%'	'81.89% ± 1.19%'	'83.65% ± 1.13%'	'85.64% ± 1.12%'	'85.89% ± 1.14%'
'SVM'	'81.29% ± 1.27%'	'82.82% ± 1.18%'	'84.1% ± 1.14%'	'85.83% ± 1.14%'	'86.55% ± 1.11%'
'RLR'	'80.77% ± 1.2%'	'82.6% ± 1.12%'	'84.41% ± 1.12%'	'86.54% ± 1.1%'	'86.67% ± 1.04%'

'kNN'	'79.95% ± 1.33%'	'80.39% ± 1.15%'	'82.09% ± 1.22%'	'84.9% ± 1.29%'	'85.1% ± 1.23%'

F1	mean F1 ± 95% CI				
**Classifier**	**5**	**10**	**20**	**40**	**50**
'RF'	'73.35% ± 1.86%'	'75.3% ± 1.52%'	77.36% ± 1.43%'	'79.99% ± 1.54%'	'81.93% ± 1.28%'
'PLS-DA'	'67.35% ± 2.17%'	'72.53% ± 2%'	'75.64% ± 1.87%'	'78.21% ± 1.86%'	'78.67% ± 1.73%'
'SVM'	'73.7% ± 1.84%'	'76.79% ± 1.52%'	'78.91% ± 1.5%'	'81.45% ± 1.42%'	'82.13% ± 1.35%'
'RLR'	'73.84% ± 1.72%'	'76.05% ± 1.58%'	'78.3% ± 1.54%'	'81.79% ± 1.53%'	'81.9% ± 1.43%'
'kNN'	'72.93% ± 1.76%'	'73.6% ± 1.52%'	'76.2% ± 1.59%'	'79.63% ± 1.64%'	'79.94% ± 1.57%'

Chi-Squared

Accuracy	mean accuracy ± 95% CI				
**Classifier**	**5**	**10**	**20**	**40**	**50**
'RF'	'65.59% ± 2.08%'	'67.94% ± 2.06%'	'70.53% ± 2.16%'	'73.65% ± 2.18%'	'74.06% ± 2.27%'
'PLS-DA'	'60.71% ± 2.23%'	'63.94% ± 2.15%'	'64.88% ± 2.35%'	'68.41% ± 2.18%'	'67.41% ± 2.06%'
'SVM'	'64.94% ± 2.22%'	'66.76% ± 2.06%'	'67.47% ± 2.06%'	'70.53% ± 2.23%'	'71.59% ± 2%'
'RLR'	'63.35% ± 2.26%'	'64.65% ± 2.11%'	'65.82% ± 2.2%'	'68.76% ± 2.04%'	'69.76% ± 2.12%'
'kNN'	'64.76% ± 2.04%'	'64.18% ± 2.19%'	'67% ± 1.99%'	'67.65% ± 2.22%'	'67.59% ± 2.01%'

Recall	mean recall ± 95% CI				
**Classifier**	**5**	**10**	**20**	**40**	**50**
'RF'	'74.13% ± 1.7%'	'75.93% ± 1.65%'	'77.88% ± 1.69%'	'80.2% ± 1.7%'	'80.54% ± 1.8%'
'PLS-DA'	'70.83% ± 1.64%'	'73.2% ± 1.58%'	'73.83% ± 1.77%'	'76.45% ± 1.63%'	'75.77% ± 1.53%'
'SVM'	'72.51% ± 2%'	'74.46% ± 1.87%'	'75.35% ± 1.78%'	'77.6% ± 1.79%'	'78.64% ± 1.62%'
'RLR'	'71.88% ± 1.94%'	'72.99% ± 1.81%'	'74.25% ± 1.71%'	'76.44% ± 1.62%'	'77.36% ± 1.59%'
'kNN'	'72.76% ± 1.84%'	'72.85% ± 1.91%'	'75.17% ± 1.66%'	'75.51% ± 1.8%'	'75.13% ± 1.88%'

Precision	mean precision ± 95% CI				
**Classifier**	**5**	**10**	**20**	**40**	**50**
'RF'	'73.46% ± 1.79%'	'75.55% ± 1.74%'	'78.29% ± 1.76%'	'80.55% ± 1.7%'	'80.85% ± 1.85%'
'PLS-DA'	'64.18% ± 2.35%'	'67.83% ± 2.25%'	'69.89% ± 2.24%'	'73.5% ± 2.14%'	'71.65% ± 2.09%'
'SVM'	'75.65% ± 2.08%'	'76.9% ± 1.8%'	'76.75% ± 1.77%'	'78.81% ± 1.73%'	'79.69% ± 1.59%'
'RLR'	'72.48% ± 1.98%'	'73.32% ± 1.79%'	'74.92% ± 1.9%'	'77.25% ± 1.65%'	'78.2% ± 1.59%'
'kNN'	'74.19% ± 1.85%'	'73.19% ± 2.04%'	'77.11% ± 1.74%'	'77.4% ± 1.85%'	'77.02% ± 1.83%'

Specificity	mean specificity ± 95% CI				
**Classifier**	**5**	**10**	**20**	**40**	**50**
'RF'	'79.31% ± 1.28%'	'80.74% ± 1.28%'	'82.21% ± 1.34%'	'84.07% ± 1.36%'	'84.32% ± 1.4%'
'PLS-DA'	'78.15% ± 1.28%'	'79.24% ± 1.27%'	'79.5% ± 1.41%'	'81.43% ± 1.31%'	'80.98% ± 1.25%'
'SVM'	'78.81% ± 1.34%'	'79.88% ± 1.27%'	'80.36% ± 1.27%'	'82.1% ± 1.38%'	'82.77% ± 1.24%'
'RLR'	'77.94% ± 1.37%'	'78.73% ± 1.3%'	'79.37% ± 1.35%'	'81.1% ± 1.29%'	'81.68% ± 1.33%'
'kNN'	'78.74% ± 1.24%'	'78.37% ± 1.34%'	'80.06% ± 1.24%'	'80.38% ± 1.4%'	'80.32% ± 1.27%'

NPV	mean NPV ± 95% CI				
**Classifier**	**5**	**10**	**20**	**40**	**50**

'RF'	'79.78% ± 1.28%'	'81.36% ± 1.28%'	'82.91% ± 1.34%'	'84.67% ± 1.36%'	'84.8% ± 1.4%'
'PLS-DA'	'78.52% ± 1.28%'	'79.71% ± 1.27%'	'79.83% ± 1.41%'	'81.85% ± 1.31%'	'81.26% ± 1.25%'
'SVM'	'80.99% ± 1.34%'	'81.47% ± 1.27%'	'81.5% ± 1.27%'	'82.83% ± 1.38%'	'83.54% ± 1.24%'
'RLR'	'78.75% ± 1.37%'	'79.33% ± 1.3%'	'80.09% ± 1.35%'	'81.9% ± 1.29%'	'82.5% ± 1.33%'
'kNN'	'79.92% ± 1.24%'	'79.46% ± 1.34%'	'81.57% ± 1.24%'	'81.87% ± 1.4%'	'81.76% ± 1.27%'

F1	mean F1 ± 95% CI				
**Classifier**	**5**	**10**	**20**	**40**	**50**
'RF'	'73.01% ± 1.69%'	'74.79% ± 1.62%'	'77.23% ± 1.65%'	'79.68% ± 1.66%'	'80.12% ± 1.76%'
'PLS-DA'	'64.37% ± 2.27%'	'68.21% ± 2.04%'	'70.23% ± 2.05%'	'73.57% ± 1.97%'	'72.17% ± 1.87%'
'SVM'	'72.59% ± 1.91%'	'73.68% ± 1.72%'	'74.72% ± 1.67%'	'77.6% ± 1.68%'	'78.38% ± 1.53%'
'RLR'	'71.3% ± 1.9%'	'72.49% ± 1.64%'	'73.74% ± 1.7%'	'75.89% ± 1.56%'	'76.97% ± 1.6%'
'kNN'	'71.92% ± 1.77%'	'71.55% ± 1.93%'	'74.24% ± 1.58%'	'74.79% ± 1.71%'	'74.88% ± 1.64%'

Variance

Accuracy	mean accuracy ± 95% CI				
**Classifier**	**5**	**10**	**20**	**40**	**50**
'RF'	'50.47% ± 1.91%'	'51.88% ± 2.35%'	'59.41% ± 1.8%'	'61.82% ± 2.11%'	'60.65% ± 1.86%'
'PLS-DA'	'36.88% ± 2.09%'	'36.65% ± 1.92%'	'48.35% ± 2.16%'	'50.12% ± 2.21%'	'49.35% ± 2.13%'
'SVM'	'47.29% ± 1.76%'	'48.12% ± 1.75%'	'49.47% ± 1.74%'	'52% ± 2.1%'	'50.18% ± 2%'
'RLR'	'44.29% ± 1.82%'	'46.71% ± 1.74%'	'45.24% ± 2.02%'	'45.06% ± 1.91%'	'48.35% ± 1.77%'
'kNN'	'42.65% ± 2.03%'	'46.53% ± 1.87%'	'59.82% ± 1.93%'	'59.65% ± 2.19%'	'57.29% ± 1.94%'

Recall	mean recall ± 95% CI				
**Classifier**	**5**	**10**	**20**	**40**	**50**
'RF'	'58.83% ± 2.17%'	'58.65% ± 2.56%'	'65.02% ± 2.1%'	'67.09% ± 2.5%'	'65.29% ± 2.38%'
'PLS-DA'	'49.14% ± 2.41%'	'48.71% ± 2.36%'	'56.68% ± 2.55%'	'57.91% ± 2.43%'	'57.66% ± 2.34%'
'SVM'	'52.74% ± 2.54%'	'53.04% ± 2.55%'	'56.05% ± 2.26%'	'61.54% ± 2.17%'	'60.17% ± 2.02%'
'RLR'	'53.98% ± 2.21%'	'52.42% ± 2.32%'	'52.42% ± 2.71%'	'55.45% ± 2.21%'	'57.4% ± 2.07%'
'kNN'	'49.63% ± 2.32%'	'52.6% ± 2.48%'	'66.35% ± 2.3%'	'68.43% ± 2.24%'	'64.71% ± 2.3%'

Precision	mean precision ± 95% CI				
**Classifier**	**5**	**10**	**20**	**40**	**50**
'RF'	'61.23% ± 2%'	'63.14% ± 2.28%'	'69.16% ± 1.81%'	'71.84% ± 1.91%'	'70.34% ± 2.05%'
'PLS-DA'	'38.98% ± 2.45%'	'41.3% ± 2.66%'	'54.47% ± 2.63%'	'58.6% ± 2.65%'	'57.42% ± 2.47%'
'SVM'	'64.08% ± 2.68%'	'65.83% ± 2.66%'	'63.9% ± 2.11%'	'64.76% ± 1.73%'	'63.3% ± 1.82%'
'RLR'	'58.78% ± 2.77%'	'63.25% ± 2.59%'	'59.06% ± 2.7%'	'61.08% ± 2.26%'	'66.39% ± 2.22%'
'kNN'	'54.59% ± 2.16%'	'58.14% ± 2.09%'	'70.01% ± 1.97%'	'69.66% ± 2.15%'	'67.58% ± 1.89%'

Specificity	mean specificity ± 95% CI				
**Classifier**	**5**	**10**	**20**	**40**	**50**
'RF'	'70.35% ± 1.13%'	'71.16% ± 1.39%'	'75.52% ± 1.1%'	'76.95% ± 1.29%'	'76.3% ± 1.13%'
'PLS-DA'	'64.54% ± 1.23%'	'63.96% ± 1.16%'	'69.74% ± 1.32%'	'70.37% ± 1.29%'	'70.01% ± 1.24%'
'SVM'	'68.09% ± 1.06%'	'68.62% ± 1.06%'	'69.47% ± 1.07%'	'71.06% ± 1.26%'	'69.92% ± 1.21%'
'RLR'	'66.53% ± 1.13%'	'67.87% ± 1.12%'	'66.94% ± 1.26%'	'66.83% ± 1.2%'	'68.8% ± 1.12%'
'kNN'	'65.43% ± 1.27%'	'67.71% ± 1.15%'	'75.82% ± 1.2%'	'75.68% ± 1.33%'	'74.13% ± 1.19%'

NPV	mean NPV ± 95% CI				
**Classifier**	**5**	**10**	**20**	**40**	**50**
'RF'	'70.73% ± 1.13%'	'71.43% ± 1.39%'	'76.09% ± 1.1%'	'77.62% ± 1.29%'	'77.1% ± 1.13%'
'PLS-DA'	'65.09% ± 1.23%'	'64.34% ± 1.16%'	'69.93% ± 1.32%'	'70.78% ± 1.29%'	'70.27% ± 1.24%'
'SVM'	'76.33% ± 1.06%'	'78.06% ± 1.06%'	'72.8% ± 1.07%'	'72.06% ± 1.26%'	'71.31% ± 1.21%'
'RLR'	'70.83% ± 1.13%'	'74.96% ± 1.12%'	'69.98% ± 1.26%'	'70.21% ± 1.2%'	'75.97% ± 1.12%'
'kNN'	'65.34% ± 1.27%'	'67.85% ± 1.15%'	'76.61% ± 1.2%'	'76.3% ± 1.33%'	'74.59% ± 1.19%'

F1	mean F1 ± 95% CI				
**Classifier**	**5**	**10**	**20**	**40**	**50**
'RF'	'58.8% ± 1.93%'	'59.62% ± 2.23%'	'66.21% ± 1.73%'	'68.34% ± 2.03%'	'66.82% ± 1.9%'
'PLS-DA'	'43.2% ± 2.17%'	'44.3% ± 2.11%'	'55.3% ± 2.16%'	'57.23% ± 2.24%'	'56.39% ± 2.19%'
'SVM'	'67.32% ± 2.77%'	'68.8% ± 2.75%'	'61.97% ± 2.13%'	'63.11% ± 1.99%'	'62.69% ± 1.89%'
'RLR'	'59.86% ± 2.42%'	'63.85% ± 2.59%'	'58.52% ± 2.59%'	'61.59% ± 2.63%'	'67.93% ± 2.74%'
'kNN'	'52.76% ± 1.9%'	'55.23% ± 1.9%'	'66.77% ± 1.89%'	'68.35% ± 1.96%'	'66.09% ± 1.81%'

Genetic Algorithm: PopSize = 50, Max NumGen= 35

Accuracy	accuracy				
**Classifier**	**5**	**10**	**20**	**40**	**50**
'RF'	'76.47%'	'70.59%'	'76.47%'	'82.35%'	'88.24%'
'PLS-DA'	'70.59%'	'76.47%'	'52.94%'	'64.71%'	'70.59%'
'SVM'	'70.59%'	'76.47%'	'58.82%'	'76.47%'	'76.47%'
'RLR'	'47.06%'	'70.59%'	'47.06%'	'64.71%'	'70.59%'
'kNN'	'47.06%'	'52.94%'	'35.29%'	'70.59%'	'52.94%'

Recall	recall				
**Classifier**	**5**	**10**	**20**	**40**	**50**
'RF'	'83.33%'	'79.17%'	'83.33%'	'87.5%'	'91.67%'
'PLS-DA'	'79.17%'	'83.33%'	'37.5%'	'75%'	'79.17%'
'SVM'	'79.17%'	'83.33%'	'70.83%'	'83.33%'	'83.33%'

'RLR'	'62.5%'	'50%'	'62.5%'	'45.83%'	'79.17%'
'kNN'	'62.5%'	'37.5%'	'25%'	'79.17%'	'37.5%'

Precision	precision				
**Classifier**	**5**	**10**	**20**	**40**	**50**
'RF'	'83.33%'	'81.21%'	'83.33%'	'90.91%'	'91.67%'
'PLS-DA'	'71.21%'	'84.44%'	'37.5%'	'61.31%'	'79.37%'
'SVM'	'79.37%'	'83.33%'	'71.52%'	'84.44%'	'84.44%'
'RLR'	'62.43%'	'70.71%'	'62.43%'	'64.58%'	'79.37%'
'kNN'	'48.15%'	'52.78%'	'35.42%'	'79.37%'	'55%'

Specificity	specificity				
**Classifier**	**5**	**10**	**20**	**40**	**50**
'RF'	'85.19%'	'81.48%'	'85.19%'	'88.89%'	'92.59%'
'PLS-DA'	'83.1%'	'85.19%'	'71.99%'	'79.4%'	'81.48%'
'SVM'	'81.48%'	'85.19%'	'74.07%'	'85.19%'	'85.19%'
'RLR'	'66.67%'	'81.48%'	'66.67%'	'77.78%'	'81.48%'
'kNN'	'68.29%'	'70.37%'	'59.26%'	'81.48%'	'70.37%'

NPV	NPV				
**Classifier**	**5**	**10**	**20**	**40**	**50**
'RF'	'85.19%'	'83.33%'	'85.19%'	'91.67%'	'92.59%'
'PLS-DA'	'84.19%'	'86.15%'	'71.99%'	'78.89%'	'81.67%'
'SVM'	'81.67%'	'85.19%'	'75%'	'86.15%'	'86.15%'
'RLR'	'66.67%'	'83.28%'	'66.67%'	'78.59%'	'81.67%'
'kNN'	'68.18%'	'70.72%'	'58.69%'	'81.67%'	'70.82%'

F1	F1				
**Classifier**	**5**	**10**	**20**	**40**	**50**
'RF'	'83.33%'	'78.41%'	'83.33%'	'87.04%'	'91.67%'
'PLS-DA'	'69.01%'	'83.07%'	'56.25%'	'65.28%'	'79.08%'
'SVM'	'79.08%'	'83.33%'	'69.77%'	'83.07%'	'83.07%'
'RLR'	'62.35%'	'72.22%'	'62.35%'	'66.54%'	'79.08%'
'kNN'	'52.19%'	'54.41%'	'36.4%'	'79.08%'	'53.08%'

Number of Generations					
**Classifier**	**5**	**10**	**20**	**40**	**50**
'RF'	35	35	35	35	35
'PLS-DA'	35	35	35	35	35
'SVM'	35	35	35	35	35
'RLR'	35	35	35	35	35
'kNN'	35	35	35	35	35

This means that in all cases the algorithm stopped after reaching the max number of generations

Genetic Algorithm: PopSize = 200, Max Num Gen = 150

Accuracy	accuracy				
**Classifier**	**5**	**10**	**20**	**40**	**50**
'RF'	'64.71%'	'70.59%'	'82.35%'	'70.59%'	'94.12%'
'PLS-DA'	'64.71%'	'58.82%'	'70.59%'	'70.59%'	'64.71%'
'SVM'	'82.35%'	'76.47%'	'76.47%'	'82.35%'	'82.35%'
'RLR'	'52.94%'	'64.71%'	'41.18%'	'52.94%'	'70.59%'
'kNN'	'35.29%'	'35.29%'	'47.06%'	'58.82%'	'70.59%'

Recall	recall				
**Classifier**	**5**	**10**	**20**	**40**	**50**
'RF'	'75%'	'79.17%'	'87.5%'	'79.17%'	'95.83%'
'PLS-DA'	'75%'	'70.83%'	'79.17%'	'79.17%'	'75%'
'SVM'	'87.5%'	'54.17%'	'83.33%'	'87.5%'	'87.5%'
'RLR'	'66.67%'	'75%'	'29.17%'	'66.67%'	'79.17%'
'kNN'	'54.17%'	'54.17%'	'33.33%'	'41.67%'	'79.17%'

Precision	precision				
**Classifier**	**5**	**10**	**20**	**40**	**50**
'RF'	'75.56%'	'79.37%'	'90.91%'	'81.21%'	'96.3%'
'PLS-DA'	'69.44%'	'56.11%'	'81.21%'	'67.78%'	'58.73%'
'SVM'	'73.61%'	'77.86%'	'84.44%'	'90.91%'	'90.91%'
'RLR'	'66.67%'	'75.56%'	'31.55%'	'66.67%'	'81.21%'
'kNN'	'38.89%'	'53.97%'	'48.33%'	'58.57%'	'79.37%'

Specificity	specificity				
**Classifier**	**5**	**10**	**20**	**40**	**50**
'RF'	'77.78%'	'81.48%'	'88.89%'	'81.48%'	'96.3%'
'PLS-DA'	'79.4%'	'77.31%'	'81.48%'	'84.72%'	'81.02%'
'SVM'	'92.13%'	'85.19%'	'85.19%'	'88.89%'	'88.89%'
'RLR'	'70.37%'	'77.78%'	'66.2%'	'70.37%'	'81.48%'
'kNN'	'60.88%'	'59.26%'	'66.67%'	'74.07%'	'81.48%'

NPV	NPV				
**Classifier**	**5**	**10**	**20**	**40**	**50**
'RF'	'78.35%'	'81.67%'	'91.67%'	'83.33%'	'96.67%'
'PLS-DA'	'81.43%'	'77.66%'	'83.33%'	'84.98%'	'80%'
'SVM'	'90.24%'	'86.61%'	'86.15%'	'91.67%'	'91.67%'
'RLR'	'73.33%'	'78.35%'	'65.93%'	'70.56%'	'83.33%'
'kNN'	'60.98%'	'59.17%'	'65.82%'	'75.18%'	'81.67%'

F1	F1				
**Classifier**	**5**	**10**	**20**	**40**	**50**
'RF'	'74.6%'	'79.08%'	'87.04%'	'78.41%'	'95.82%'
'PLS-DA'	'63.74%'	'55.56%'	'78.41%'	'64.81%'	'61.11%'
'SVM'	'74.4%'	'78.89%'	'83.07%'	'87.04%'	'87.04%'

'RLR'	'61.21%'	'74.6%'	'45.42%'	'66.14%'	'78.41%'
'kNN'	'43.51%'	'53.99%'	'40.43%'	'60%'	'79.08%'

Number of Generations

Classifier	5	10	20	40	50
'RF'	64	65	70	73	94
'PLS-DA'	62	60	87	82	83
'SVM'	64	63	75	69	74
'RLR'	150	150	139	150	115
'kNN'	51	51	51	51	51

Rsp100_FeaturesSets_CLL_SUB_111

mRMR

Accuracy — mean accuracy ± 95% CI

Classifier	20%	30%	40%	50%	60%	70%	80%
'RF'	'79.41% ± 1.81%'	'77.41% ± 1.83%'	'77.12% ± 2.09%'	'76.59% ± 1.95%'	'75.88% ± 2.05%'	'75.41% ± 2.07%'	'76.12% ± 1.98%'
'PLS-DA'	'81.88% ± 1.45%'	'80.47% ± 1.5%'	'80.88% ± 1.39%'	'79.94% ± 1.56%'	'79.59% ± 1.6%'	'79.71% ± 1.63%'	'80.24% ± 1.42%'
'SVM'	'81.53% ± 1.74%'	'80.29% ± 1.67%'	'81.24% ± 1.54%'	'80.12% ± 1.79%'	'78.65% ± 1.79%'	'79% ± 1.76%'	'78.47% ± 1.73%'
'RLR'	'77.88% ± 1.85%'	'77.71% ± 1.66%'	'77.47% ± 1.7%'	'77.71% ± 1.68%'	'77.53% ± 1.7%'	'78.76% ± 1.67%'	'77.18% ± 1.62%'
'kNN'	'64.65% ± 1.71%'	'62.53% ± 2.06%'	'58.76% ± 2%'	'58.35% ± 1.98%'	'58.06% ± 1.92%'	'58.24% ± 1.93%'	'58.82% ± 1.9%'

Recall — mean recall ± 95% CI

Classifier	20%	30%	40%	50%	60%	70%	80%
'RF'	'84.82% ± 1.34%'	'83.09% ± 1.5%'	'82.74% ± 1.73%'	'81.94% ± 1.78%'	'81.86% ± 1.68%'	'81.36% ± 1.67%'	'81.86% ± 1.74%'
'PLS-DA'	'86.56% ± 1.08%'	'85.54% ± 1.11%'	'85.86% ± 1.04%'	'85.19% ± 1.16%'	'84.95% ± 1.18%'	'85.02% ± 1.2%'	'85.39% ± 1.05%'
'SVM'	'86.21% ± 1.39%'	'85.17% ± 1.38%'	'85.78% ± 1.37%'	'84.95% ± 1.48%'	'83.76% ± 1.59%'	'84.01% ± 1.57%'	'83.59% ± 1.53%'
'RLR'	'81.55% ± 2.08%'	'81.06% ± 1.94%'	'81.44% ± 1.8%'	'81.35% ± 1.84%'	'81.02% ± 1.88%'	'82.88% ± 1.58%'	'80.67% ± 1.81%'
'kNN'	'71.98% ± 1.84%'	'69.56% ± 2.14%'	'66.43% ± 2.12%'	'64.8% ± 2.29%'	'65.26% ± 2.23%'	'65.39% ± 2.09%'	'65.48% ± 2.18%'

Precision — mean precision ± 95% CI

Classifier	20%	30%	40%	50%	60%	70%	80%
'RF'	'86.19% ± 1.35%'	'84.5% ± 1.43%'	'84.34% ± 1.6%'	'83.82% ± 1.51%'	'83.51% ± 1.57%'	'83.13% ± 1.56%'	'83.56% ± 1.59%'
'PLS-DA'	'85.85% ± 1.41%'	'85.18% ± 1.33%'	'84.52% ± 1.46%'	'83.89% ± 1.61%'	'84% ± 1.6%'	'83.93% ± 1.55%'	'84.24% ± 1.42%'
'SVM'	'87.29% ± 1.27%'	'86.44% ± 1.25%'	'87.23% ± 1.19%'	'86.48% ± 1.34%'	'85.4% ± 1.38%'	'85.68% ± 1.36%'	'85.14% ± 1.35%'
'RLR'	'84.62% ± 1.56%'	'84.45% ± 1.39%'	'84.46% ± 1.4%'	'84.59% ± 1.38%'	'84.55% ± 1.42%'	'85.78% ± 1.27%'	'84.45% ± 1.3%'
'kNN'	'77.35% ± 1.8%'	'74.91% ± 2.11%'	'71.17% ± 2.25%'	'71.03% ± 2.25%'	'70.35% ± 2.14%'	'70.23% ± 2.27%'	'71.37% ± 1.86%'

Specificity — mean specificity ± 95% CI

Classifier	20%	30%	40%	50%	60%	70%	80%
'RF'	'87.55% ± 1.11%'	'86.32% ± 1.13%'	'86.12% ± 1.3%'	'85.84% ± 1.19%'	'85.4% ± 1.28%'	'85.1% ± 1.27%'	'85.51% ± 1.23%'
'PLS-DA'	'89.28% ± 0.87%'	'88.38% ± 0.92%'	'88.82% ± 0.83%'	'88.27% ± 0.92%'	'87.97% ± 0.95%'	'88.05% ± 0.98%'	'88.36% ± 0.87%'
'SVM'	'88.81% ± 1.06%'	'88.06% ± 1.01%'	'88.66% ± 0.94%'	'87.98% ± 1.08%'	'87.09% ± 1.08%'	'87.31% ± 1.06%'	'87% ± 1.04%'
'RLR'	'86.56% ± 1.14%'	'86.49% ± 1.02%'	'86.34% ± 1.04%'	'86.51% ± 1.03%'	'86.39% ± 1.06%'	'87.15% ± 1.03%'	'86.2% ± 1%'
'kNN'	'78.55% ± 1.05%'	'77.23% ± 1.28%'	'75% ± 1.23%'	'74.72% ± 1.23%'	'74.6% ± 1.16%'	'74.71% ± 1.18%'	'75.08% ± 1.14%'

NPV	mean NPV ± 95% CI						
**Classifier**	**20%**	**30%**	**40%**	**50%**	**60%**	**70%**	**80%**
'RF'	'88.54% ± 1.11%'	'87.26% ± 1.13%'	'87.14% ± 1.3%'	'86.78% ± 1.19%'	'86.46% ± 1.28%'	'86.13% ± 1.27%'	'86.56% ± 1.23%'
'PLS-DA'	'89.75% ± 0.87%'	'88.94% ± 0.92%'	'89.3% ± 0.83%'	'88.84% ± 0.92%'	'88.55% ± 0.95%'	'88.61% ± 0.98%'	'88.85% ± 0.87%'
'SVM'	'89.5% ± 1.06%'	'88.87% ± 1.01%'	'89.5% ± 0.94%'	'88.89% ± 1.08%'	'88.07% ± 1.08%'	'88.3% ± 1.06%'	'87.92% ± 1.04%'
'RLR'	'87.52% ± 1.14%'	'87.41% ± 1.02%'	'87.33% ± 1.04%'	'87.53% ± 1.03%'	'87.52% ± 1.06%'	'88.27% ± 1.03%'	'87.38% ± 1%'
'kNN'	'81.69% ± 1.05%'	'79.91% ± 1.28%'	'77.44% ± 1.23%'	'76.94% ± 1.23%'	'77.08% ± 1.16%'	'76.75% ± 1.18%'	'77.37% ± 1.14%'

F1	mean F1 ± 95% CI						
**Classifier**	**20%**	**30%**	**40%**	**50%**	**60%**	**70%**	**80%**
'RF'	'84.42% ± 1.37%'	'82.9% ± 1.4%'	'82.46% ± 1.64%'	'81.87% ± 1.62%'	'81.55% ± 1.59%'	'81.16% ± 1.59%'	'81.78% ± 1.54%'
'PLS-DA'	'85.25% ± 1.29%'	'84.42% ± 1.23%'	'83.95% ± 1.31%'	'83.19% ± 1.46%'	'83.27% ± 1.43%'	'83.25% ± 1.41%'	'83.67% ± 1.25%'
'SVM'	'85.98% ± 1.39%'	'85.08% ± 1.28%'	'85.71% ± 1.22%'	'84.86% ± 1.39%'	'83.76% ± 1.41%'	'84.02% ± 1.39%'	'83.64% ± 1.36%'
'RLR'	'82.23% ± 1.65%'	'81.86% ± 1.49%'	'81.9% ± 1.47%'	'81.99% ± 1.45%'	'81.88% ± 1.41%'	'83.08% ± 1.42%'	'81.4% ± 1.43%'
'kNN'	'70.76% ± 1.62%'	'69.07% ± 1.86%'	'66.13% ± 1.91%'	'65.42% ± 1.87%'	'65.73% ± 1.9%'	'66.03% ± 1.62%'	'65.31% ± 1.79%'

Relief-F

Accuracy	mean accuracy ± 95% CI						
**Classifier**	**20%**	**30%**	**40%**	**50%**	**60%**	**70%**	**80%**
'RF'	'78.41% ± 1.94%'	'78.06% ± 1.75%'	'77.47% ± 1.85%'	'77.18% ± 1.77%'	'76.53% ± 1.82%'	'75.29% ± 1.84%'	'75.71% ± 2.02%'
'PLS-DA'	'81.59% ± 1.64%'	'80.65% ± 1.75%'	'80.76% ± 1.75%'	'79.82% ± 1.78%'	'79.12% ± 1.69%'	'79.06% ± 1.83%'	'78.88% ± 1.72%'
'SVM'	'81.94% ± 1.89%'	'80.59% ± 1.77%'	'80.29% ± 1.88%'	'80.18% ± 1.82%'	'79.88% ± 1.77%'	'79.41% ± 1.78%'	'78.71% ± 1.74%'
'RLR'	'76.35% ± 1.73%'	'76.53% ± 1.75%'	'77.53% ± 1.9%'	'78.12% ± 1.73%'	'78% ± 1.7%'	'77.82% ± 1.69%'	'77.65% ± 1.7%'
'kNN'	'70.24% ± 2.1%'	'67% ± 2.13%'	'66.47% ± 2.25%'	'64.06% ± 2.19%'	'63.53% ± 2.33%'	'62.47% ± 2.04%'	'62.18% ± 2.13%'

Recall	mean recall ± 95% CI						
**Classifier**	**20%**	**30%**	**40%**	**50%**	**60%**	**70%**	**80%**
'RF'	'83.68% ± 1.64%'	'83.41% ± 1.48%'	'83.05% ± 1.52%'	'82.67% ± 1.63%'	'82.17% ± 1.6%'	'81.26% ± 1.65%'	'81.2% ± 1.77%'
'PLS-DA'	'86.39% ± 1.22%'	'85.7% ± 1.29%'	'85.76% ± 1.3%'	'85.08% ± 1.33%'	'84.52% ± 1.27%'	'84.51% ± 1.37%'	'84.4% ± 1.28%'
'SVM'	'86.41% ± 1.56%'	'85.27% ± 1.51%'	'85.08% ± 1.55%'	'84.96% ± 1.51%'	'84.66% ± 1.57%'	'84.3% ± 1.57%'	'83.78% ± 1.54%'
'RLR'	'79.62% ± 1.82%'	'80.03% ± 1.73%'	'81.02% ± 1.84%'	'81.76% ± 1.71%'	'81.33% ± 1.87%'	'81.6% ± 1.77%'	'81.04% ± 1.96%'
'kNN'	'76.95% ± 1.9%'	'73.79% ± 2.11%'	'72.73% ± 2.29%'	'71.15% ± 2.34%'	'69.57% ± 2.65%'	'69.96% ± 2.22%'	'69.91% ± 2.17%'

Precision	mean precision ± 95% CI						
**Classifier**	**20%**	**30%**	**40%**	**50%**	**60%**	**70%**	**80%**

'RF'	'85.29% ± 1.51%'	'85.12% ± 1.35%'	'84.48% ± 1.43%'	'84.48% ± 1.45%'	'84.12% ± 1.41%'	'83.07% ± 1.49%'	'83.3% ± 1.55%'
'PLS-DA'	'84.62% ± 1.56%'	'83.17% ± 1.72%'	'83.85% ± 1.69%'	'83.55% ± 1.75%'	'83.16% ± 1.68%'	'83.11% ± 1.74%'	'83.19% ± 1.65%'
'SVM'	'87.44% ± 1.39%'	'86.41% ± 1.35%'	'86.28% ± 1.39%'	'86.37% ± 1.35%'	'86.2% ± 1.35%'	'85.9% ± 1.36%'	'85.38% ± 1.34%'
'RLR'	'83.93% ± 1.33%'	'83.91% ± 1.34%'	'84.72% ± 1.46%'	'85.33% ± 1.27%'	'85.14% ± 1.3%'	'84.94% ± 1.3%'	'84.77% ± 1.39%'
'kNN'	'79.98% ± 1.7%'	'77.43% ± 1.78%'	'76.69% ± 2.06%'	'75.33% ± 1.99%'	'74.46% ± 2.12%'	'74.19% ± 1.92%'	'73.99% ± 1.89%'

Specificity	mean specificity ± 95% CI						
**Classifier**	**20%**	**30%**	**40%**	**50%**	**60%**	**70%**	**80%**
'RF'	'86.91% ± 1.19%'	'86.71% ± 1.07%'	'86.33% ± 1.14%'	'86.21% ± 1.08%'	'85.79% ± 1.13%'	'85.01% ± 1.14%'	'85.31% ± 1.24%'
'PLS-DA'	'89.28% ± 0.99%'	'88.82% ± 1.05%'	'88.84% ± 1.03%'	'88.16% ± 1.05%'	'87.71% ± 1%'	'87.74% ± 1.08%'	'87.57% ± 1.04%'
'SVM'	'89.06% ± 1.15%'	'88.24% ± 1.08%'	'88.08% ± 1.14%'	'87.99% ± 1.11%'	'87.83% ± 1.08%'	'87.54% ± 1.08%'	'87.13% ± 1.05%'
'RLR'	'85.65% ± 1.07%'	'85.79% ± 1.08%'	'86.37% ± 1.17%'	'86.73% ± 1.07%'	'86.64% ± 1.06%'	'86.55% ± 1.05%'	'86.43% ± 1.04%'
'kNN'	'82% ± 1.27%'	'80% ± 1.31%'	'79.72% ± 1.38%'	'78.28% ± 1.35%'	'77.96% ± 1.43%'	'77.3% ± 1.25%'	'77.12% ± 1.3%'

NPV	mean NPV ± 95% CI						
**Classifier**	**20%**	**30%**	**40%**	**50%**	**60%**	**70%**	**80%**
'RF'	'87.93% ± 1.19%'	'87.78% ± 1.07%'	'87.27% ± 1.14%'	'87.35% ± 1.08%'	'86.96% ± 1.13%'	'86.17% ± 1.14%'	'86.37% ± 1.24%'
'PLS-DA'	'89.63% ± 0.99%'	'89.18% ± 1.05%'	'89.25% ± 1.03%'	'88.63% ± 1.05%'	'88.3% ± 1%'	'88.39% ± 1.08%'	'88.14% ± 1.04%'
'SVM'	'89.71% ± 1.15%'	'88.89% ± 1.08%'	'88.77% ± 1.14%'	'88.83% ± 1.11%'	'88.75% ± 1.08%'	'88.5% ± 1.08%'	'88.09% ± 1.05%'
'RLR'	'86.78% ± 1.07%'	'86.81% ± 1.08%'	'87.44% ± 1.17%'	'87.9% ± 1.07%'	'87.8% ± 1.06%'	'87.6% ± 1.05%'	'87.62% ± 1.04%'
'kNN'	'83.62% ± 1.27%'	'81.67% ± 1.31%'	'81.25% ± 1.38%'	'80.25% ± 1.35%'	'79.81% ± 1.43%'	'79.36% ± 1.25%'	'79.08% ± 1.3%'

F1	mean F1 ± 95% CI						
**Classifier**	**20%**	**30%**	**40%**	**50%**	**60%**	**70%**	**80%**
'RF'	'83.5% ± 1.52%'	'83.21% ± 1.37%'	'82.89% ± 1.42%'	'82.55% ± 1.41%'	'81.9% ± 1.49%'	'81.1% ± 1.44%'	'81.29% ± 1.6%'
'PLS-DA'	'84.34% ± 1.47%'	'83.27% ± 1.52%'	'83.41% ± 1.65%'	'83.08% ± 1.62%'	'82.53% ± 1.56%'	'82.37% ± 1.66%'	'82.54% ± 1.5%'
'SVM'	'86.32% ± 1.49%'	'85.26% ± 1.39%'	'85.02% ± 1.47%'	'84.88% ± 1.43%'	'84.71% ± 1.39%'	'84.35% ± 1.39%'	'83.81% ± 1.37%'
'RLR'	'80.34% ± 1.56%'	'80.65% ± 1.53%'	'81.53% ± 1.64%'	'82.18% ± 1.51%'	'81.98% ± 1.52%'	'82% ± 1.51%'	'81.78% ± 1.53%'
'kNN'	'76.48% ± 1.8%'	'73.46% ± 1.91%'	'73.06% ± 1.99%'	'71.1% ± 2.02%'	'69.94% ± 2.32%'	'69.81% ± 1.95%'	'69.4% ± 1.97%'

Chi-Squared

Accuracy	mean accuracy ± 95% CI						
**Classifier**	**20%**	**30%**	**40%**	**50%**	**60%**	**70%**	**80%**

'RF'	'78.35% ± 1.69%'	'78.47% ± 1.92%'	'77.71% ± 2.01%'	'78.53% ± 1.94%'	'76.82% ± 1.89%'	'76.88% ± 2%'	'75.24% ± 1.98%'
'PLS-DA'	'79.24% ± 1.69%'	'80.41% ± 1.59%'	'80.35% ± 1.55%'	'79.47% ± 1.6%'	'80% ± 1.5%'	'79.76% ± 1.62%'	'79.76% ± 1.65%'
'SVM'	'79.76% ± 1.68%'	'79.71% ± 1.67%'	'80.29% ± 1.73%'	'80.65% ± 1.6%'	'79.59% ± 1.78%'	'79.71% ± 1.81%'	'78.94% ± 1.73%'
'RLR'	'77.59% ± 1.86%'	'76.71% ± 1.72%'	'76.65% ± 1.72%'	'78% ± 1.68%'	'78.29% ± 1.72%'	'78.35% ± 1.67%'	'78% ± 1.72%'
'kNN'	'61.94% ± 1.99%'	'61.12% ± 2.01%'	'59.71% ± 1.85%'	'58.94% ± 1.79%'	'58.47% ± 1.76%'	'58.65% ± 1.68%'	'58.06% ± 1.81%'

Recall	mean recall ± 95% CI						
**Classifier**	**20%**	**30%**	**40%**	**50%**	**60%**	**70%**	**80%**
'RF'	'84% ± 1.24%'	'83.63% ± 1.64%'	'83.1% ± 1.79%'	'83.89% ± 1.49%'	'82.49% ± 1.62%'	'82.41% ± 1.84%'	'81.22% ± 1.77%'
'PLS-DA'	'84.6% ± 1.27%'	'85.48% ± 1.19%'	'85.44% ± 1.16%'	'84.8% ± 1.18%'	'85.23% ± 1.1%'	'85.03% ± 1.2%'	'85.05% ± 1.21%'
'SVM'	'84.71% ± 1.42%'	'84.5% ± 1.54%'	'84.95% ± 1.61%'	'85.23% ± 1.47%'	'84.43% ± 1.62%'	'84.51% ± 1.65%'	'83.96% ± 1.56%'
'RLR'	'81.52% ± 2.01%'	'81.11% ± 1.7%'	'80.19% ± 1.98%'	'81.85% ± 1.81%'	'81.84% ± 1.84%'	'81.7% ± 1.92%'	'81.63% ± 1.83%'
'kNN'	'68.37% ± 2.17%'	'68.33% ± 2.08%'	'66.15% ± 2.18%'	'65.11% ± 2.37%'	'64.71% ± 2.18%'	'65.04% ± 2.19%'	'64.6% ± 2.13%'

Precision	mean precision ± 95% CI						
**Classifier**	**20%**	**30%**	**40%**	**50%**	**60%**	**70%**	**80%**
'RF'	'85.23% ± 1.24%'	'85.29% ± 1.5%'	'84.76% ± 1.58%'	'85.28% ± 1.44%'	'84.23% ± 1.48%'	'83.98% ± 1.65%'	'82.97% ± 1.61%'
'PLS-DA'	'83.5% ± 1.62%'	'84.21% ± 1.6%'	'83.28% ± 1.62%'	'83.64% ± 1.58%'	'83.78% ± 1.59%'	'83.32% ± 1.73%'	'83.87% ± 1.59%'
'SVM'	'86.07% ± 1.32%'	'85.82% ± 1.31%'	'86.37% ± 1.36%'	'86.71% ± 1.23%'	'85.93% ± 1.38%'	'86.09% ± 1.42%'	'85.62% ± 1.36%'
'RLR'	'84.44% ± 1.52%'	'84.09% ± 1.42%'	'84% ± 1.38%'	'85.26% ± 1.24%'	'85.43% ± 1.27%'	'85.22% ± 1.36%'	'85.3% ± 1.25%'
'kNN'	'73.55% ± 2.1%'	'73.36% ± 2.01%'	'71.87% ± 2.2%'	'71.07% ± 2.12%'	'71.43% ± 1.9%'	'70.98% ± 2.09%'	'70.88% ± 1.87%'

Specificity	mean specificity ± 95% CI						
**Classifier**	**20%**	**30%**	**40%**	**50%**	**60%**	**70%**	**80%**
'RF'	'86.87% ± 1.05%'	'86.99% ± 1.17%'	'86.51% ± 1.24%'	'87% ± 1.19%'	'85.98% ± 1.15%'	'86% ± 1.22%'	'85.01% ± 1.22%'
'PLS-DA'	'87.71% ± 1.03%'	'88.44% ± 0.95%'	'88.58% ± 0.93%'	'87.9% ± 0.98%'	'88.25% ± 0.91%'	'88.14% ± 0.97%'	'88.11% ± 0.99%'
'SVM'	'87.75% ± 1.04%'	'87.71% ± 1.02%'	'88.07% ± 1.06%'	'88.29% ± 0.98%'	'87.64% ± 1.08%'	'87.71% ± 1.1%'	'87.27% ± 1.05%'
'RLR'	'86.41% ± 1.14%'	'85.88% ± 1.05%'	'85.84% ± 1.06%'	'86.66% ± 1.04%'	'86.83% ± 1.06%'	'86.87% ± 1.03%'	'86.64% ± 1.06%'
'kNN'	'76.9% ± 1.23%'	'76.43% ± 1.25%'	'75.56% ± 1.15%'	'75.11% ± 1.1%'	'74.88% ± 1.08%'	'74.95% ± 1.05%'	'74.64% ± 1.11%'

NPV	mean NPV ± 95% CI						
**Classifier**	**20%**	**30%**	**40%**	**50%**	**60%**	**70%**	**80%**
'RF'	'87.78% ± 1.05%'	'87.94% ± 1.17%'	'87.57% ± 1.24%'	'87.86% ± 1.19%'	'87.06% ± 1.15%'	'87.02% ± 1.22%'	'86.1% ± 1.22%'

'PLS-DA'	'88.26% ± 1.03%'	'88.88% ± 0.95%'	'88.97% ± 0.93%'	'88.47% ± 0.98%'	'88.66% ± 0.91%'	'88.65% ± 0.97%'	'88.69% ± 0.99%'
'SVM'	'88.55% ± 1.04%'	'88.48% ± 1.02%'	'88.91% ± 1.06%'	'89.16% ± 0.98%'	'88.52% ± 1.08%'	'88.65% ± 1.1%'	'88.26% ± 1.05%'
'RLR'	'87.41% ± 1.14%'	'87.02% ± 1.05%'	'87.03% ± 1.06%'	'87.93% ± 1.04%'	'88.08% ± 1.06%'	'88.05% ± 1.03%'	'87.95% ± 1.06%'
'kNN'	'78.7% ± 1.23%'	'78.77% ± 1.25%'	'77.62% ± 1.15%'	'77.09% ± 1.1%'	'77.15% ± 1.08%'	'77.06% ± 1.05%'	'76.81% ± 1.11%'

F1	mean F1 ± 95% CI						
**Classifier**	**20%**	**30%**	**40%**	**50%**	**60%**	**70%**	**80%**
'RF'	'83.6% ± 1.29%'	'83.49% ± 1.51%'	'82.96% ± 1.58%'	'83.63% ± 1.5%'	'82.24% ± 1.51%'	'82.27% ± 1.63%'	'81.05% ± 1.56%'
'PLS-DA'	'82.89% ± 1.49%'	'83.86% ± 1.42%'	'83.08% ± 1.43%'	'82.88% ± 1.46%'	'83.32% ± 1.4%'	'82.88% ± 1.5%'	'83.19% ± 1.44%'
'SVM'	'84.67% ± 1.28%'	'84.63% ± 1.29%'	'85.03% ± 1.36%'	'85.33% ± 1.23%'	'84.47% ± 1.41%'	'84.55% ± 1.44%'	'83.99% ± 1.35%'
'RLR'	'82.12% ± 1.62%'	'81.3% ± 1.51%'	'80.95% ± 1.61%'	'82.25% ± 1.57%'	'82.37% ± 1.58%'	'82.58% ± 1.44%'	'82.13% ± 1.6%'
'kNN'	'68.69% ± 1.78%'	'68.26% ± 1.79%'	'66.57% ± 1.67%'	'65.45% ± 1.84%'	'64.64% ± 1.75%'	'64.86% ± 1.74%'	'65.1% ± 1.68%'

Variance

Accuracy	mean accuracy ± 95% CI						
**Classifier**	**20%**	**30%**	**40%**	**50%**	**60%**	**70%**	**80%**
'RF'	'78.18% ± 2.02%'	'77.71% ± 1.83%'	'76.88% ± 2.1%'	'77.06% ± 1.78%'	'76.65% ± 1.8%'	'76.24% ± 1.9%'	'75.35% ± 2.11%'
'PLS-DA'	'77.06% ± 1.72%'	'76.71% ± 1.78%'	'76.88% ± 1.66%'	'77.53% ± 1.57%'	'78.71% ± 1.57%'	'78.65% ± 1.47%'	'78.82% ± 1.55%'
'SVM'	'80.65% ± 1.55%'	'78.71% ± 1.65%'	'78.12% ± 1.63%'	'79.29% ± 1.47%'	'78.12% ± 1.57%'	'77.94% ± 1.7%'	'78.12% ± 1.7%'
'RLR'	'76.18% ± 1.81%'	'77.18% ± 1.9%'	'77.88% ± 1.8%'	'76.94% ± 1.65%'	'76.53% ± 1.51%'	'76.12% ± 1.59%'	'76% ± 1.75%'
'kNN'	'61% ± 1.9%'	'60.06% ± 2.04%'	'60.35% ± 1.57%'	'60.41% ± 1.82%'	'58.06% ± 1.68%'	'57.12% ± 1.79%'	'57.94% ± 1.82%'

Recall	mean recall ± 95% CI						
**Classifier**	**20%**	**30%**	**40%**	**50%**	**60%**	**70%**	**80%**
'RF'	'83.4% ± 1.68%'	'83.28% ± 1.49%'	'82.82% ± 1.6%'	'82.95% ± 1.36%'	'82.51% ± 1.45%'	'82.35% ± 1.41%'	'81.66% ± 1.59%'
'PLS-DA'	'82.95% ± 1.31%'	'82.72% ± 1.31%'	'82.87% ± 1.23%'	'83.39% ± 1.17%'	'84.26% ± 1.16%'	'84.18% ± 1.1%'	'84.36% ± 1.14%'
'SVM'	'85.68% ± 1.16%'	'84.23% ± 1.23%'	'83.68% ± 1.26%'	'84.49% ± 1.18%'	'83.58% ± 1.23%'	'83.33% ± 1.42%'	'83.38% ± 1.51%'
'RLR'	'81.7% ± 1.55%'	'81.74% ± 1.82%'	'81.58% ± 1.86%'	'80.83% ± 1.67%'	'80.25% ± 1.49%'	'80.77% ± 1.62%'	'79.82% ± 1.83%'
'kNN'	'69.55% ± 1.94%'	'68.7% ± 2.07%'	'68.29% ± 1.86%'	'67.82% ± 2.12%'	'65.8% ± 2.05%'	'64.85% ± 2.25%'	'65.71% ± 2.32%'

Precision	mean precision ± 95% CI						
**Classifier**	**20%**	**30%**	**40%**	**50%**	**60%**	**70%**	**80%**
'RF'	'85.06% ± 1.5%'	'84.67% ± 1.41%'	'83.96% ± 1.66%'	'84.3% ± 1.38%'	'83.92% ± 1.35%'	'83.67% ± 1.54%'	'83.31% ± 1.61%'
'PLS-DA'	'79.9% ± 1.79%'	'80.62% ± 1.78%'	'81.41% ± 1.71%'	'81.63% ± 1.66%'	'82.98% ± 1.48%'	'82.82% ± 1.45%'	'83.09% ± 1.59%'

'SVM'	'86.43% ± 1.19%'	'85.02% ± 1.24%'	'84.84% ± 1.23%'	'85.81% ± 1.07%'	'85.02% ± 1.21%'	'84.87% ± 1.29%'	'84.97% ± 1.3%'
'RLR'	'83.45% ± 1.41%'	'84.26% ± 1.57%'	'84.92% ± 1.42%'	'84.42% ± 1.31%'	'84.11% ± 1.17%'	'83.67% ± 1.26%'	'83.51% ± 1.43%'
'kNN'	'71.81% ± 1.6%'	'71.21% ± 1.89%'	'71.71% ± 1.53%'	'72.37% ± 1.81%'	'69.76% ± 1.83%'	'68.9% ± 1.97%'	'68.96% ± 2.04%'

Specificity	mean specificity ± 95% CI						
**Classifier**	**20%**	**30%**	**40%**	**50%**	**60%**	**70%**	**80%**
'RF'	'86.77% ± 1.24%'	'86.49% ± 1.13%'	'86.03% ± 1.29%'	'86.13% ± 1.1%'	'85.86% ± 1.12%'	'85.63% ± 1.17%'	'85.07% ± 1.3%'
'PLS-DA'	'86.73% ± 1.03%'	'86.32% ± 1.09%'	'86.4% ± 0.99%'	'86.83% ± 0.95%'	'87.43% ± 0.97%'	'87.42% ± 0.9%'	'87.61% ± 0.92%'
'SVM'	'88.28% ± 0.96%'	'87.1% ± 1.02%'	'86.74% ± 1.01%'	'87.48% ± 0.91%'	'86.76% ± 0.97%'	'86.69% ± 1.03%'	'86.79% ± 1.03%'
'RLR'	'85.52% ± 1.12%'	'86.15% ± 1.16%'	'86.61% ± 1.11%'	'86.06% ± 1.03%'	'85.81% ± 0.93%'	'85.53% ± 0.98%'	'85.45% ± 1.09%'
'kNN'	'76.39% ± 1.16%'	'75.81% ± 1.24%'	'75.98% ± 0.96%'	'76.04% ± 1.1%'	'74.61% ± 1.02%'	'74.01% ± 1.11%'	'74.53% ± 1.14%'

NPV	mean NPV ± 95% CI						
**Classifier**	**20%**	**30%**	**40%**	**50%**	**60%**	**70%**	**80%**
'RF'	'87.71% ± 1.24%'	'87.39% ± 1.13%'	'86.84% ± 1.29%'	'87.06% ± 1.1%'	'86.77% ± 1.12%'	'86.64% ± 1.17%'	'86.24% ± 1.3%'
'PLS-DA'	'87.06% ± 1.03%'	'86.77% ± 1.09%'	'86.92% ± 0.99%'	'87.45% ± 0.95%'	'88% ± 0.97%'	'87.98% ± 0.9%'	'88.34% ± 0.92%'
'SVM'	'88.83% ± 0.96%'	'87.7% ± 1.02%'	'87.51% ± 1.01%'	'88.27% ± 0.91%'	'87.65% ± 0.97%'	'87.63% ± 1.03%'	'87.75% ± 1.03%'
'RLR'	'86.38% ± 1.12%'	'87.18% ± 1.16%'	'87.62% ± 1.11%'	'87.21% ± 1.03%'	'86.93% ± 0.93%'	'86.61% ± 0.98%'	'86.52% ± 1.09%'
'kNN'	'77.16% ± 1.16%'	'76.79% ± 1.24%'	'77.18% ± 0.96%'	'77.89% ± 1.1%'	'76.25% ± 1.02%'	'75.61% ± 1.11%'	'75.87% ± 1.14%'

F1	mean F1 ± 95% CI						
**Classifier**	**20%**	**30%**	**40%**	**50%**	**60%**	**70%**	**80%**
'RF'	'83.31% ± 1.59%'	'83.13% ± 1.38%'	'82.66% ± 1.52%'	'82.57% ± 1.37%'	'82.13% ± 1.44%'	'81.83% ± 1.49%'	'81.12% ± 1.62%'
'PLS-DA'	'79.81% ± 1.62%'	'80.34% ± 1.57%'	'80.82% ± 1.55%'	'81.05% ± 1.47%'	'82.38% ± 1.34%'	'82.2% ± 1.29%'	'82.12% ± 1.49%'
'SVM'	'85.48% ± 1.16%'	'83.98% ± 1.25%'	'83.4% ± 1.26%'	'84.24% ± 1.17%'	'83.31% ± 1.22%'	'83.14% ± 1.36%'	'83.36% ± 1.35%'
'RLR'	'81.57% ± 1.49%'	'82.02% ± 1.55%'	'82.17% ± 1.54%'	'81.36% ± 1.36%'	'80.76% ± 1.26%'	'81.14% ± 1.33%'	'80.45% ± 1.5%'
'kNN'	'69.3% ± 1.72%'	'68.44% ± 1.81%'	'68.27% ± 1.5%'	'67.86% ± 1.78%'	'65.96% ± 1.65%'	'65.35% ± 1.87%'	'66.15% ± 1.82%'
